# Topological mixing and irreversibility in animal chromosome evolution

**DOI:** 10.1126/sciadv.adz5561

**Published:** 2026-08-19

**Authors:** Darrin T. Schultz, Arno Blümel, Dalila Destanović, Fatih Sarigol, Oleg Simakov

**Affiliations:** ^1^ Department of Neuroscience and Developmental Biology, University of Vienna, Vienna 1010, Austria.; ^2^ Department of Ecology and Evolutionary Biology, Yale University, New Haven, CT 06520, USA.

## Abstract

Animal chromosome homology can persist over hundreds of millions of years, despite fusions and translocations. The frequency, pace, and impact of these changes remain unclear. We develop a multiscale manifold representation of pan-animal genome homology to compare 5821 chromosome-scale genomes across 19 phyla and 4454 species. This “evolutionary genome topology” approach simultaneously captures chromosomal and subchromosomal organization. We find that while all 406 pairwise fusions of 29 ancestral animal linkage groups have been sampled by metazoan genome diversity, the full combinatorial potential within chromosomes remains far from explored. Our approach shows that irreversible genomic changes, caused in particular by chromosomal consolidation, dissociation, and fusion-with-mixing, place clades in distinct regions of genome architecture space. Progressive accumulation of these mixed states across genomic scales contributes to the diverging paths of animal genome evolution and has a long-lasting impact on a broad range of genes, including key developmental loci.

## INTRODUCTION

Animal chromosomes have remained remarkably conserved since the last common animal ancestor (*1*–*3*) that existed over 700 million years ago (*4*) and even retain some conservation with chromosomes of the closest protist relatives of animals (*2*, *3*). These portions of chromosomes that are conserved over time across many species are called ancestral linkage groups (ALGs) (*5*) or chromosomal elements (*6*) and are the relicts of ancient chromosomes. Comparisons across anciently diverged animal phyla indicate that the ancestor of Myriazoa [all animals except ctenophores (*3*)] had chromosomes that were a complement of at least 29 ALGs (*2*). As conserved representatives of ancient chromosomes, ALGs are a useful tool that enable the analysis of how chromosomes changed over hundreds of millions of years, especially when direct sequence comparisons are impossible because of divergence.

Many animal chromosomes can be explained by three additive combinations of complete 29 myriazoan ALGs, collectively referred to as “chromosomal algebra” (*2*): Robertsonian or end-to-end fusion, centric insertion, and fusion-with-mixing (FWM). In FWM, two chromosomes or ancestral elements fuse and subsequently intermix via intrachromosomal translocations, such as inversions. This process produces thoroughly interleaved gene orders that are statistically irreversible, and, therefore, shared FWM patterns provide strong phylogenetic characters (*2*, *3*). However, the prevalence of these additive chromosomal ALG–preserving changes involving conserved chromosomal identities across all animal phyla, their long-term (macro-)evolutionary effects, and how they change possible evolutionary trajectories remain poorly understood.

Inferring these changes in chromosome evolution relies on detectable chromosomal homology, which is eroded over time through dispersal (*2*, *7*). Dispersal is the process of orthologous genes moving from one source chromosome to another through interchromosomal translocations. Interchromosomal translocations are considered to be rare events as a portion of gametes produced after translocation are unbalanced and selected against (*8*). Therefore, organisms that reproduce sexually or have not passed through substantial population bottlenecks are expected to retain their ancestral chromosomal complement, while asexually reproducing organisms may avoid this selection and fix translocations because of drift. Quantification of chromosomal translocations across different animal clades has been hampered by the lack of chromosome-scale assemblies. The only metazoan baseline estimate for gene dispersal out of chromosomes is 1% per 40 million years (*2*). However, whether and how this rate is different among clades or ALGs of different sizes, as well as the functional implications of dispersal or its absence, remain unknown.

On top of these descriptions involving combinations of whole chromosomes or at least chromosomal arms, several transitions in animal karyotypes have been reported that do not fit clearly within the framework of the three additive ALG-preserving chromosomal changes. These ALG-disrupting chromosomal changes include fissions and/or large interchromosomal translocations that break chromosomal element homology. In particular, there are animal clades in which the ancestral animal chromosomal elements have partly or wholly dispersed or rearranged beyond recognizability. Clades with these rearrangements include the genome reshuffling in cephalopods (*9*), the derived karyotype of ctenophores (*3*), the “Merian elements” of lepidopterans (*10*), and the “Nigon elements” of nematodes (*11*). Clades with rearrangements beyond recognition include the “Muller elements” of drosophilids (*6*, *12*) and clitellate annelids (*13*–*15*). Whether and how these transitions can still be explained through extensive combination of complete ancestral chromosomal elements or whether breakage of chromosomes is required to achieve these states remains to be investigated. In either case, these departures from ancestral animal chromosomal elements, some of which may occur rapidly on short evolutionary branches, can result in what we would recognize as new sets of clade-specific chromosomal elements. Whether and how these rapid chromosomal changes are followed by ALG-preserving “stasis” retaining ancestral chromosomal identities or changes that break intact chromosomal units remain unclear (*9*, *16*).

The long-term impact of these processes on the evolution of functions such as gene regulation remains unclear. No global functional enrichment in any chromosomal element has been reported (*2*, *3*). While this suggests that chromosome-level changes could reflect a random process, chromosomal FWM may have a fundamental and barely studied outcome: By both expanding the chromosomal coordinate system and enabling new combinations of genes to enter proximity, it may drive gene regulatory evolution and phenotypic innovations, as was proposed for cephalopod genomes (*17*).

There are known regulatory constraints on local genome structure, including coexpression (*18*) and coregulation (*19*), and it has been suggested that genome-wide changes in chromosomal composition may help drive (*20*) or strengthen (*21*) novel genomic interactions. The functional importance of the conservation and change in the animal genome architecture among different animal clades remains a nascent research topic because of the limited gene regulatory information for many of these clades. However, it is clear that ancient local gene linkages can be conserved as functional regulatory units (*19*, *22*) and that chromosomal changes can (*17*) and do (*23*) coincide with the evolution of novel gene colocalizations and potential regulatory linkages and phenotypic novelties (*23*–*25*). Despite the importance of new genomic configurations and regulatory interactions as an element of the evolution of novelty, there is not a comprehensive understanding of the dynamics of new regulatory interactions arising in animal evolution.

**Fig. 1.**
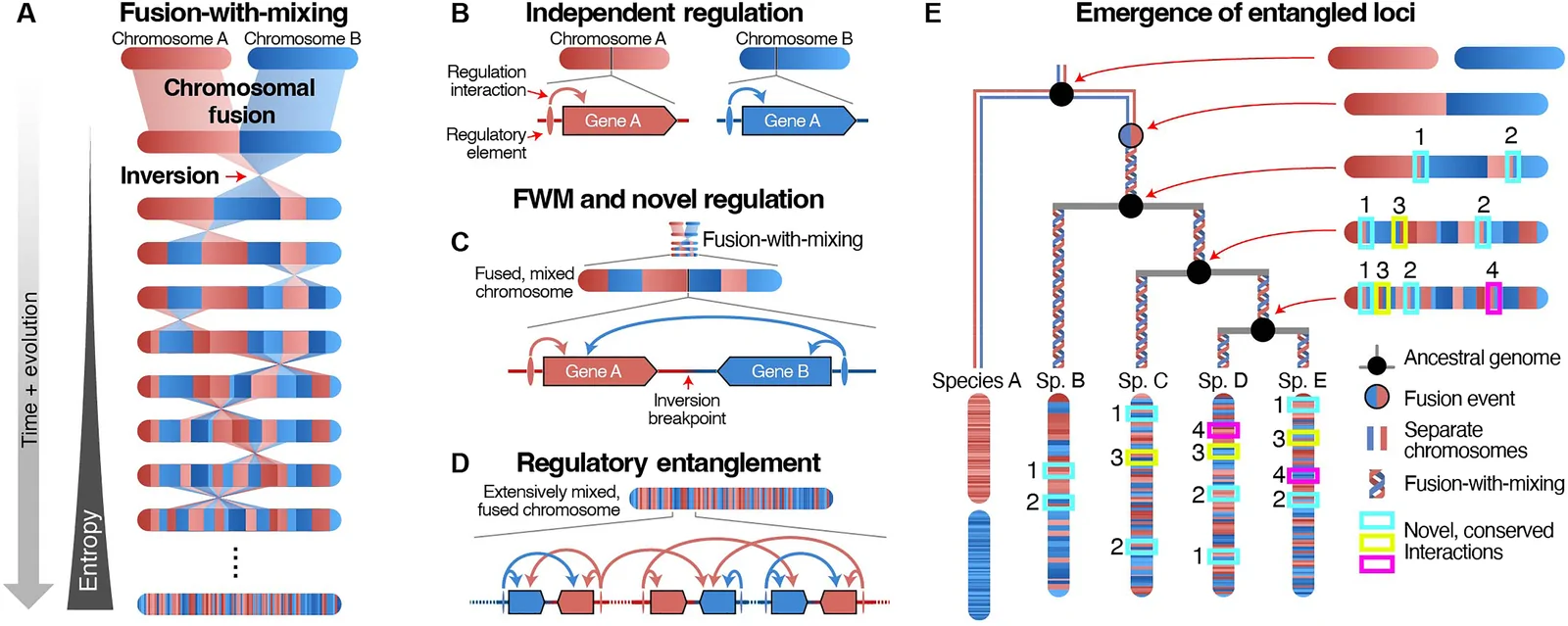
FWM at chromosomal and subchromosomal levels. (**A**) FWM leads to progressive mixing of chromosomal regions over evolutionary time. Red and blue bars represent two ancestral chromosomes before (top) and after (second row) fusion; cross-links illustrate postfusion inversions that progressively interleave the segments; the left wedge indicates increasing mixing entropy through time. (**B**) In separate chromosomes, regulatory elements (ovals) act on nearby genes within their chromosome (arrows). (**C**) Through FWM, loci from different ancestral chromosomes can come into proximity with each other’s regulatory elements. (**D**) Over time, novel regulatory interactions can arise between previously separate loci and become constrained through mixing (red and blue arrows), potentially preventing reversal to the ancestral unmixed state (the mixed state is entangled). This regulatory entanglement model is one possible explanation for the stable locus pairs we observe; neutral persistence and other selective pressures may also contribute. (**E**) Emergence of entangled loci on a phylogeny. Black circles, ancestral nodes; red-blue split, prefusion state; half circle, fusion event; helical icons, postfusion mixing. Colored boxes mark four novel red-blue regulatory interactions (1 to 4) arising after inversions; earlier interactions (cyan, 1 and 2) are retained in more lineages (species B to E) than later ones (yellow, 3; magenta, 4).

Answering these questions has only become possible in recent years. Over the past decade, advances in long-read sequencing, Hi-C–based scaffolding, and the coordination of large biodiversity consortia have accelerated sampling across Metazoa from 45 species with chromosome-scale assemblies in 2015 to thousands today, moving the field beyond a model-centric era and into broad phylogenetic coverage and data inundation (*26*, *27*). This order-of-magnitude growth now allows us to address the questions posed above: What are the dynamics of chromosome evolution across animal diversity, and when do effectively irreversible mixing events coincide with or eventually lead to the emergence of new genomic neighborhoods and macroevolutionary shifts in diversity? Determining the timescale and relationships among large chromosomal changes and the evolution of new genomic configurations and potential regulatory linkages is key to understanding the evolutionary potential of animal chromosomes and the emergence of novelties (Fig. 1). There is a great need for more tools and analytical frameworks to assess chromosome-scale changes at the tree-of-life scale and across large divergences of time and sequence similarity.

Using a broad sampling of 5821 metazoan chromosome-scale genomes, we address these questions and contribute comparative tools by identifying general trends of multiscale chromosomal changes. We quantify the timescale of large chromosomal changes and characterize their macroevolutionary impact onto the emergence of novel gene neighborhoods and potential regulatory interactions. We develop a set of approaches we call “evolutionary genome topology” (EGT), which uses manifold-based methods to compare genomes across scales, from local gene order to whole-chromosome composition, within a single framework. By integrating information across genomic scales that existing methods treat separately, EGT can identify clade-specific genomic configurations, such as novel gene colocalizations that arise through chromosomal mixing and can persist for hundreds of millions of years. Our results using the EGT approach show that each clade can be characterized by its own set of “mixed” chromosomal and subchromosomal characters. This approach augments FWM, which is a process at the chromosomal level, and reveals a similar process of mixing and subsequent stable association of postmixing locus pairs at the subchromosomal level. After FWM brings genes and their regulatory elements from different ancestral regions into proximity, resulting in locally mixed states, some newly neighboring loci may develop functional interactions that are maintained by selection, preserving their colocalization across the clade and, over time, blurring the boundaries between the originally distinct genomic regions or erasing the boundaries altogether (*28*). We call this process “entanglement.” Our findings suggest that animal genome evolution is a stepwise process of these largely irreversible changes and the simultaneous erosion and accumulation of mixed states at different levels of genomic organization. These findings furthermore will help guide further functional testing of these novel putatively regulatory states.

## RESULTS

### Ancestral chromosomal homology retention and completeness of metazoan genomes

To analyze chromosome evolution across animals, we compiled a curated, chromosome-scale dataset from all National Center for Biotechnology Information (NCBI)–indexed genomes comprising 5821 assemblies from 4454 species, representing the state of the art in genomics sequencing and assembly datasets (see the “Constructing a genome database” section and table S1). Genomes under embargo from publication were not included in this dataset (Materials and Methods). The database spans 59% of recognized animal phyla (19 of 32), 60% of classes (62 of 104), and 46% of orders (294 of 644) and includes all nonbilaterian phyla except Placozoa (Materials and Methods). To capture intraspecific structural variation, we retain multiple assemblies per species and, where available, phased haplotypes from the same individual.

To study the history of chromosome evolution across these animal species, we needed an effective way to trace changes among chromosomes, many of which had been diverging for hundreds of millions of years. Because whole-genome alignments yield very fragmented or incomplete orthologies at such deep divergences (*29*) and because de novo gene-based orthology is often limited by missing or inaccurate gene predictions, we used 2361 gene families conserved across animals as orthologous markers. Each gene family was present in 1 of the 29 ALGs in the ancestral myriazoan genome (*2*, *3*, *7*). Following convention, we refer to these as the bilaterian-cnidarian-sponge (BCnS) ALGs (*2*). Across the 5821 assemblies (4454 species), we inferred orthology to BCnS ALGs (see the “Identifying BCnS ALGs” section) and estimated the BCnS ALG persistence, fusions, and fissions at the species level (table S2) and clade level for all taxonomic nodes in our dataset (see the “Inferring ALG evolutionary history” Methods section and table S3).

This approach allowed us to characterize the presence and combinations of the 29 BCnS ALGs. All of the 406 (29 choose 2) possible combinations of BCnS ALG fusion pairs were present at least once as either fused (●) or fused-and-mixed (⊗) in this complete dataset, suggesting that all possible combinations have been explored by evolution. Within individual animal clades, the patterns of chromosomal changes varied. For example, the cnidarians are known for their well-preserved BCnS ALG chromosomal complement (*2*). For a set of 109 chromosome-scale cnidarian genomes (*n* = 96 species), all of the 29 BCnS ALGs were detected in at least one species, totaling 218 of 406 possible ALG fusion combinations, meaning that 188 ALG fusion combinations did not appear in any of the 96 cnidarian species. Of the 218 observed ALG fusion combinations, 97 (44% of 218) were species specific. These estimates of fusion represent a lower limit, as these counts can be affected by species with poorly assembled chromosomes or chromosomes that have rearranged to the point where certain ALGs are dispersed across chromosomes. Contrary to cnidarians, in nematodes, a clade known for its genome contraction and a rearrangement-rich history (*11*), 27 of 29 ALGs were detected in at least one of 139 genomes from 86 species. In addition, the ALG composition varied strongly between nematodes. The median number was 19 ALGs per nematode species, compared to a median of 29 in cnidarians. Similarly, in strong contrast to cnidarians, seven nematode species completely lacked recognizable ALGs. Accounting for the two undetectable BCnS ALGs in nematodes, 145 of total 351 possible fusions were present, 43 of which were species specific (30% of 145), comprising a similar proportion as in cnidarians. Of these 145 fusions in nematodes, 72 were fusions observed in at least one cnidarian genome, which is expected given the low karyotype number in nematodes and the enhanced retention of larger ALGs across animals (see fig. S1 and the “Discussion” section).

### The timescale of chromosomal element homology dispersal

**Fig. 2.**
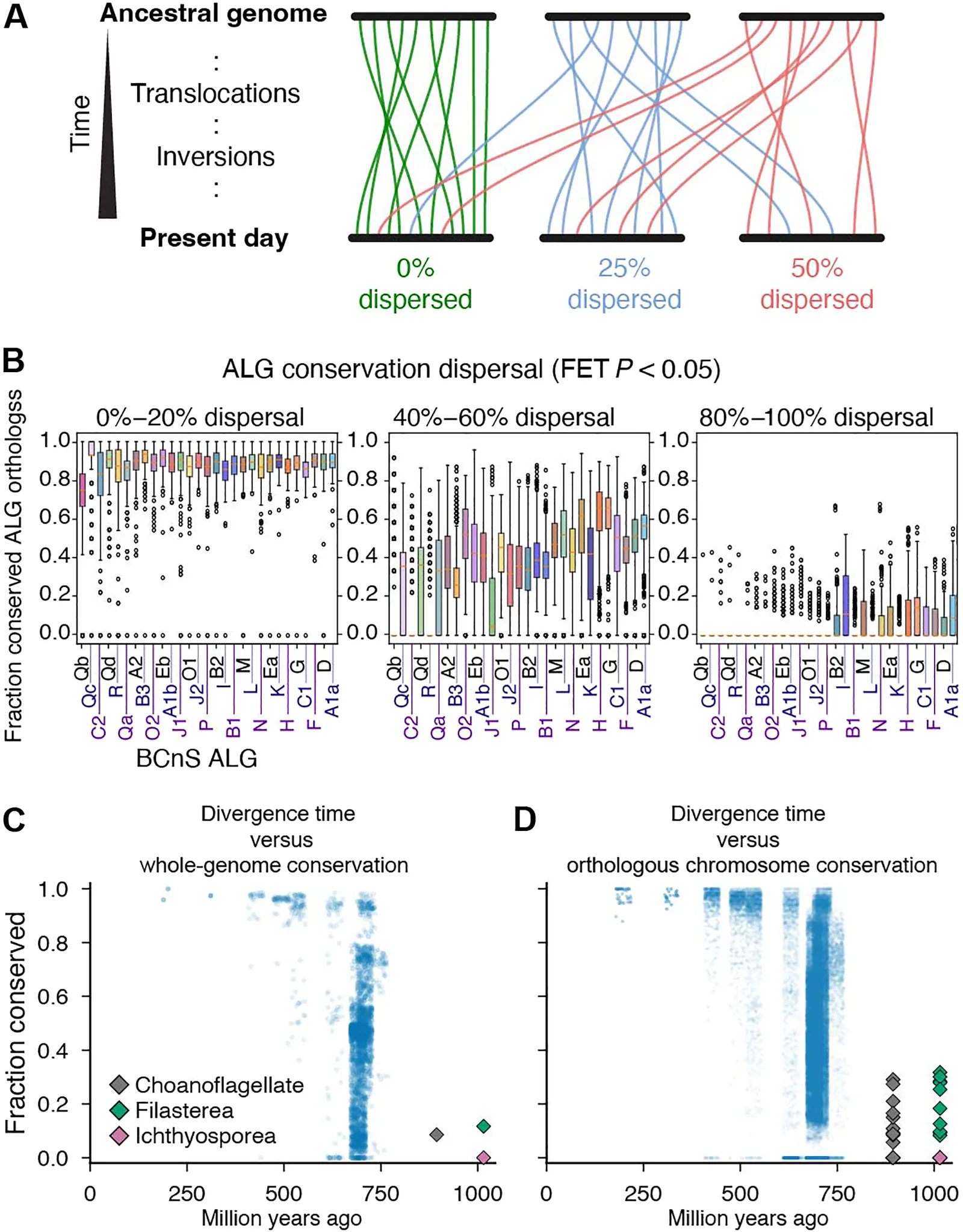
Time dependence of BCnS ALG dispersal. (**A**) Dispersal is the process of loci leaving their chromosome of origin through translocations. Inversions over time cause loci that were previously on separate chromosomes to mix. (**B**) Gene dispersal in animal genomes (*n* = 4348 species and genomes) varies greatly depending on the evolutionary history of the species since the BCnS ancestor. Some species retain nearly 100% of BCnS ALGs as recognizable on one or more chromosomes, while some species retain no recognizable BCnS ALGs (*P* ≤ 0.05, Bonferroni-corrected one-sided Fisher’s exact test, FET). Genomes are binned by the total percent of BCnS ALG dispersal across the entire genome and colored by BCnS ALG. Individual BCnS ALG’s dispersal values for each genome are plotted for the box plots. Box centerlines are the median values, box limits span from the first quartile to the third quartile, fliers span to 1.5 times the interquartile range (Q3 to Q1), and outliers are plotted individually. (**C**) We compared the chromosome conservation of the whole *P. maximus* genome to the genomes of other animals (*n* = 4348 species). The *y* axis shows the total fraction of orthologs conserved in the genome. The decay of homology is a time-dependent process, with a rate of slowed change between present day and 600 million years ago. (**D**) The same data, but the conservation values of individual *Pecten* chromosomes relative to the other species are shown (*n* = 4348 species).

We next characterized dispersal, a process in which interchromosomal translocations cause genes to move from one ALG to other chromosomes. We investigated these properties by computing the animal genome gene dispersal (*n* = 5821 genomes) relative to the BCnS ALGs (see the “Dispersal characterization” section). The average gene dispersal relative to the BCnS ALG complement is as low as 0.2% for chromosomally highly preserved genomes such as the scallop *Mimachlamys varia* (Fig. 2B) to between 53 and 73% for genomes in the nematode genus *Caenorhabditis*, reflecting partial BCnS ALG dispersal, and 100% for species such as *Drosophila* or *Anopheles* whose genomes are highly rearranged and the BCnS ALGs are no longer detectable (Fig. 2 and table S4).

The total dispersal scales with evolutionary time and the lineage in question. For example, a comparison of the genomes from two closely related bivalve species exhibits very little dispersal. Comparing genomes of animal or unicellular species with a common ancestor at the Choanozoa, Filozoa, or Holozoa nodes (Fig. 2, C and D, red boxes) reveals that, on the other hand, very few chromosomal elements are conserved (almost complete dispersal).

We find that the number of dispersed orthologs is independent of the chromosomal element size (Fig. 2 and fig. S2). This has several important implications. First, it enables predicting the life span of a given chromosomal homology. For example, because larger chromosomes have more positions available for homology detection (not necessarily protein-coding genes), their homology can remain detectable longer than that of the smaller chromosomes. The smallest chromosome of the scallop *Pecten maximus* [18 BCnS orthogroups in 32.5 million base pairs (Mb), BCnS ALG C2], for example, is unrecognizable in some species in our dataset that diverged over 550 million years ago, while the homology with larger *Pecten* chromosomes remains detectable (Fig. 2). For these reasons, while even the smaller chromosomes are still likely to persist within animals, without sensitive analyses (*3*), the same chromosomal homologies are completely undetectable in animal-unicell comparisons. The second implication is that for smaller chromosomes, homology detection either will rely on a shorter evolutionary distance or, with larger evolutionary distance, will rely more on them having fused with other chromosomes. One example is the bilaterian linkage group Q element (*2*, *7*), which became colocalized on the same chromosome in the ancestor of chordates from four very small elements (Qa, Qb, Qc, and Qd) that are also found colocalized in various combinations in other animal clades. Under this model, smaller ancestral elements are predictably undetectable in certain clades, a prominent example being BCnS ALG R, which is not present in chordate genomes (*2*).

We found that per-clade dispersal differences are not driven by homology detection failure. To test this, we computed for each of the 1037 NCBI-annotated species the fraction of the 2361 BCnS gene families (*2*) recovered by reciprocal best-hit hidden Markov model (HMM) search against its protein predictions. Median recovery is 97.4% and exceeds 90% in every clade we compared (fig. S2). Within Vertebrata (*n* = 606), recovery and synteny conservation are uncorrelated (Spearman ρ = −0.08). Nematoda is the only clade with a strong recovery-conservation correlation (ρ = 0.79, *n* = 21), consistent with concurrent rapid sequence evolution, gene loss, and karyotype reduction. Together, these data clarify the time decay of BCnS chromosomal element homology and highlight the process of dispersal that happens along deeply conserved chromosomes.

### Changes in rates of chromosomal element evolution

**Fig. 3.**
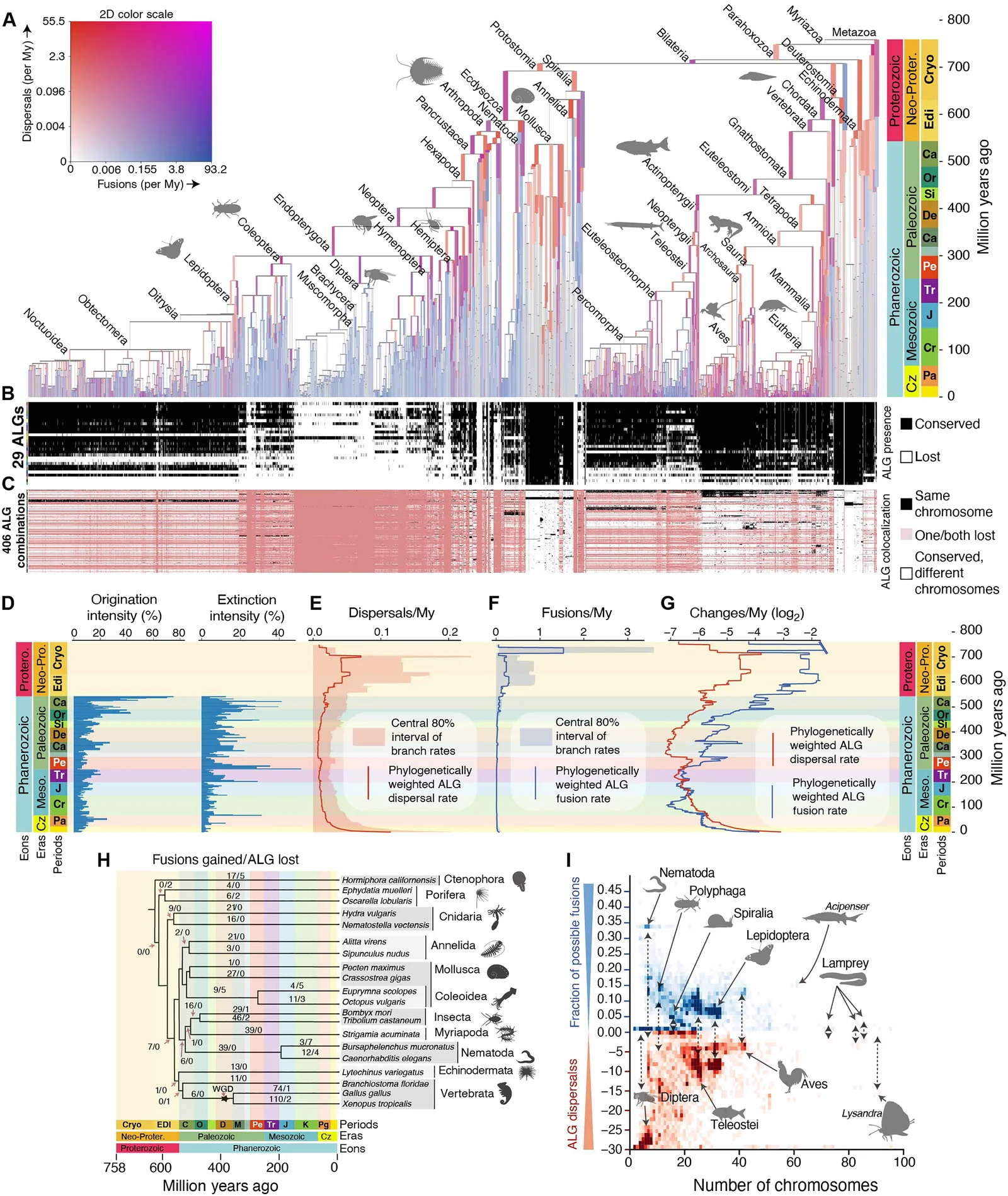
Global patterns of chromosomal fusions and fissions across metazoans. (**A**) Chromosome evolutionary history along the phylogenetic tree of 4402 species. BCnS ALG losses through dispersal are red, and BCnS ALG fusions are blue. My, million years. (**B**) ALG presence (pixels) across the species (columns). Black, ALG presence on one or more chromosomes; white, ALG was not identified. (**C**) Same as (B), showing the presence or absence of two ALGs paired on a chromosome in that species’ genome. Black, both ALGs are present on the same chromosome; white, ALG pair is not present on the same chromosome; pink, lack of detectability because of a lost BCnS ALG homology. (**D**) Species origination and extinction intensity from (*31*). (**E**) A plot of the rate of ALG dispersals per million years. The light red range (*x* axis) shows the central 80% interval of measured dispersal rates on all branches at that time point, while the solid red line is the phylogenetically weighted average rate of dispersal. (**F**) Same as (E), showing ALG fusions per million years. (**G**) Data from (E) and (F) plotted in logarithmic scale and without intervals. (**H**) Shared changes at major evolutionary transitions. For key internal nodes, boxes report counts of fusions gained/ALG dispersals inferred on that branch and retained by sampled descendants. Horizontal position gives the node age (million years); background colors indicate geologic periods. (**I**) A heatmap of the relationship of chromosome number to the lineage-specific number of chromosome fusions and losses through dispersal (*n* = 5674 genomes). Each cell is the total count of all genomes in that bin. Top (blue): For each genome, the fraction of detectable ALG pairs that are on the same chromosome (“fraction of possible fusions”) plotted against its chromosome count. Bottom (red): The fraction of ALG homologies lost versus chromosome count.

Slow chromosomal evolution is a marked feature in many animal clades (*3*, *7*, *17*) and is characterized by the presence and preservation of the BCnS ALGs, each on a single or a small number of chromosomes. However, it is unclear how much the fusion and dispersal rates can vary throughout evolution. We developed a phylogeny-aware method to profile fusion, fission, and dispersal of ALGs (Fig. 3, A and B, and table S5). Our analysis confirmed that fusion rates were consistent within clades for any given BCnS ALG combination, without a clear preference for smaller ALGs. However, fusion or translocation rates varied across clades, ranging from 0.8825 to 1.07 fusions per million years in less genomically derived clades, such as spiralians or sponges (table S6 and fig. S1).

Our results also show that chromosome evolution has not occurred at a steady rate; fusion and dispersal vary in timing and intensity (Fig. 3, table S7, and fig. S3). The highest BCnS ALG rearrangement rates occurred along the branches leading to major extant clades (Fig. 3A). Notably, across all animals, the average fusion rate increased during the end-Ediacaran 550 million years ago, peaking at ∼0.25 fusions per million years in the Ediacaran (Fig. 3F), before declining to ∼0.01 fusions per million years by the Jurassic Period (Fig. 3, E and F, and table S8). For most of the Phanerozoic, homology loss through dispersal rates were approximately half or less that of fusion rates (Fig. 3E), with peaks in the Proterozoic (0.05 losses through dispersal per million years) and after the Cretaceous. We note, however, that the few sampled branches before the Cambrian make the measurement of changes on these deep branches of the animal tree challenging (Fig. 3, D to F). Furthermore, average dispersal rates across animals appear higher in the most recent branches of the animal tree, from 100 million years ago to present, because of the effect of incorporating highly rearranged genomes in the measurement (Fig. 3D).

These cases of increased rates of chromosome rearrangements can result in observed saltatory changes along an evolutionary branch, in which the genomes of the extant species are highly rearranged relative to typical animal chromosomes with intact BCnS ALGs, and these changes are restricted to particular epochs. In some clades, for example the Arthropoda, after an initial high rate of fusions and dispersals at the beginning of the clade diversification during the Cambrian ∼580 million years ago (*30*) (0.25 fusions per million years), these changes slowed to a rate similar to that of other animals by present day (fig. S3, A and F, and table S9). In other clades, such as mosquitoes or clitellate annelids, the changes appear to happen so rapidly that we did not observe remnants of transitionary genomic states in closely related species and therefore cannot estimate rates of change in those branches using BCnS ALGs.

With genome-derived chromosomal evolutionary rates in hand, we tested for correlations between chromosomal change rates and species origination or extinction (*31*) (see Materials and Methods and tables S9 to S11). Signals were clade specific. In one of the most species-rich groups in our dataset, the protostomes (*n* = 3394 genomes), chromosomal fusion rates weakly correlated with species extinction rates (one-tailed Spearman’s rank correlation, *r*_s_ = 0.56, *P* < 1 × 10^−45^, *n* = 542 million-year bins) and more weakly with origination (*r*_s_ = 0.39, *P* < 1 × 10^−19^, *n* = 542). However, in vertebrates (*n* = 2007 genomes), fusion rates were weakly correlated with extinction (fusions, *r*_s_ = 0.49, *P* < 1 × 10^−33^), and dispersals were weakly anticorrelated with extinction (dispersals, *r*_s_ = −0.47, *P* < 1 × 10^−31^; losses through dispersal, *r*_s_ = −0.25, *P* < 1 × 10^−7^; *n* = 542) (fig. S3, E to L, and tables S10 and S11). Our data also point to potential evidence of long-term patterns, such as period fluctuations of chromosomal change rates (see Materials and Methods, Supplementary Text, figs. S3 and S4, and table S12); however, more precise node dating and a robust phylogeny will be required for future investigation.

### Consolidation and dissociation of chromosomal elements

The balancing act between dynamic rates of chromosomal fusions and translocations has had a substantial role in shaping distinct genomic organizations across animals (*2*, *32*, *33*). Using our pan-metazoan dataset, we examined whether the observed chromosomal evolutionary patterns could be categorized according to their evolutionary mechanisms (see the “Inferring ALG evolutionary history” Methods section). Given that the ancestral myriazoan (*3*) genome comprised 20 to 30 chromosomes (*2*), we hypothesized that extant species with fewer than 20 chromosomes would exhibit different evolutionary patterns than those with more than 30. Our analysis identifies two mechanistically distinct modes of karyotype evolution, consolidation and dissociation, that cause deviations from the ancestral myriazoan karyotype, alongside a mode of karyotypic stability (Fig. 3I).

The predominant pattern we can observe in animal genomes with few chromosomes is that ALGs remain recognizable after fusions. This consolidation mode of chromosome evolution occurs when fusion rates exceed fission or translocation rates, which likewise must remain low for ALG preservation. Nematodes exemplify this pattern. Despite their rapid sequence evolution (*34*, *35*) and previous suggestions of having a new chromosomal set (*36*), nematode genomes (*n* = 139 genomes, 86 species) can still be described as combinations of retained ALGs. BCnS ALGs are largely retained as complete units, with only one shared BCnS homology loss through dispersal (ALG C2) across the clade (Fig. 3, B and D, and table S3). Similarly, oyster chromosomes (Ostreidae, *n* = 17 genomes, 8 species) are the result of 52 chromosomal fusions, yet all species retain the full set of 29 BCnS ALGs intact on single chromosomes. Importantly, karyotype reduction through fusion leaves genomes more susceptible to subsequent dispersal, resulting in the observed mode of evolution often associated with “faster evolving” genomes.

Genomes that have substantially increased their chromosome counts since the ancestral myriazoan karyotype could have done so through two dissociative processes. The first dissociation pattern involves chromosomal fissions or translocations that split BCnS ALGs across many chromosomes yet show weak or no ALG dispersal. The second dissociation pattern, whole-genome duplication, expands karyotype number and often results in secondary gene loss on duplicated homeologous chromosomes, as seen in vertebrates (*7*, *37*, *38*). Despite distinct mechanisms, both dissociation processes produce similar syntenic patterns, stemming from the increase in karyotype number that can undergo additional fusions (*9*). However, the nature of fissions and translocations in the first pattern prevents these genomes from being represented as a combination of complete BCnS ALGs, necessitating a new ALG notation. Chromosomal fissions may result from holocentricity (*39*), centric fissions, or possibly chromothripsis (*40*), i.e., chromosomal restructuring in a single generation (*41*). Clades exemplifying this pattern include coleoid cephalopods (*17*) (Fig. 3D) and clitellate annelids (*13*). Vertebrates, by contrast, can fully be represented by whole-genome duplications and BCnS ALG combinations (*2*, *7*).

Our analysis also supports a conservative mode of animal chromosome evolution, characterized by an overall conservation of ancestral chromosomal element identities, with few interchromosomal rearrangements or fusions, more closely resembling the myriazoan (BCnS) karyotype (*2*, *3*). We stress that while species with genomes in this category have very few interchromosomal rearrangements, they still exhibit extensive intrachromosomal rearrangements that change the locus order and orientation within chromosomes over time.

Together, these results point to three predominant modes of chromosome evolution in animal genomes since divergence from the ancestral myriazoan genome. Consolidation and dissociation are inherently distinct processes, shaped by different timings and degrees of fusion, fission, and dispersal, and likely reflect different meiotic constraints and population dynamics. Lineages that undergo consolidation or dissociation depart from the ancestral myriazoan chromosomal complement in ways that irrevocably change the substrate for subsequent chromosomal evolutionary events. Last, it is also possible that over longer evolutionary times one mode of chromosomal evolution could follow after another.

### Macroevolutionary impact of chromosomal fusion processes

Our results reveal distinct evolutionary paths from the ancestral BCnS ALG karyotype to clade-specific chromosomal element compositions. However, the impact, if any, of the ever-changing chromosomal substrate on novel gene regulation is unknown. To explore this, we investigated the impact of FWM and, more generally, macroevolutionary accumulation of chromosomal inversions on the emergence of local subchromosomal and potentially regulatory linkages (*19*, *21*, *42*, *43*).

First, to characterize mixing timescales, we simulated inversion dynamics using a beads-on-a-string genome model (see the “Simulation of FWM” section). We define τ_s_ as the number of inversion cycles needed to reach a fully mixed, high-entropy state (table S13 and fig. S5) and compared τ_s_ across chromosome sizes. Smaller chromosomes (100-gene fusion products) reached τ_s_ quickly after ∼50 inversion cycles, while larger chromosomes (1000 genes) required ∼1000 cycles. Direct measurements from cephalopod genomes (*n* = 4 species; see the “Inversion breakpoint inference” section) indicate rates of 4.29 to 29.2 inversions per million years (*44*), suggesting that a 1000-gene chromosome would take 34.2 to 233.1 million years to reach a highly entropic state. These results suggest that achieving an effectively irreversible mixed state after chromosome fusion is size dependent and can occur relatively quickly on a macroevolutionary timescale, especially for smaller chromosomes.

As a proxy for local regulatory linkages, we measured the extent of novel neighborhood exploration using our chromosome inversion model (see the “Simulation of FWM” section and fig. S5). We define τ_50_ as the number of inversion cycles required to explore 50% of all possible neighborhoods, defined as direct neighbors or genes within windows of 2, 5, or 10 genes. These neighborhoods can reflect structural and regulatory compartments of animal genomes such as topologically associating domains (TADs), in which genes may be coregulated (*45*, *46*). Similar to τ_s_, τ_50_ increases with chromosome size but over much longer timescales. Reaching 50% of possible direct neighbor interactions requires ∼1700 inversion cycles for a 100-gene chromosome (25 × τ_s_) and ∼173,000 cycles for a 1000-gene chromosome (130 × τ_s_, *n* = 10 trials; fig. S5), orders of magnitude longer than to achieve the initial entropically mixed state. Direct measurements of the mixing value *m* (*3*) in ancient FWM events in the scallop *P. maximus* confirm that entropic saturation through inversions has not yet been reached (table S14). These results suggest that for an average metazoan chromosome that has been conserved over 600 or more million years, as in *Pecten*, most potential interactions remain unexplored.

Persistent topological properties can substantially reduce τ_50_ by maintaining distal gene interactions. Accounting for these neighborhoods lowers τ_50_ substantially, and treating all genes as neighbors within five-gene neighborhoods reduces τ_50_ by 93.5% (*n* = 10 trials). This suggests that stable interactive domains (e.g., TADs) may enable faster evolutionary exploration of new genomic configurations, as genomes with TAD-scale interactions will explore combinations faster than genomes dominated by cis-interactions with immediate neighbors. Consistent with this, our results on genome rearrangements (see the “Inversion breakpoint inference” section) and previous work (*47*–*49*) highlight that inversion breakpoints tend to happen at insulator regions, i.e., TAD boundaries, consistent with possible selective constraint to maintain genes within the same compartments. We therefore expect that nonbilaterian animal genomes, which appear to lack insulated compartments because of the lack of CCCTC-binding factor (CTCF) or similar insulator factors (*50*, *51*), would explore their potential for genomic innovation more slowly than genomes capable of establishing and maintaining more distal regulatory interactions, as in bilaterians. At the same time, shorter regulatory distances may enable faster reshuffling of chromosomes since inversions do not break existing regulatory linkages (*28*).

The long-term nature of continuous emergence of novel local neighborhoods on old or newly formed chromosomal elements describes an evolutionary process on the macroevolutionary timescale (Fig. 1). However, whether and how local neighborhoods can be considered an irreversible FWM-like process and the probabilities of a new subchromosomal FWM event given the macrosyntenic background remain unexplored. Existing regulatory interactions and the genomic regions that they span play a central role in the evolution of these loci, as is the case for the Hox clusters (*21*) and many other micro-syntenic clusters (*19*, *43*, *52*). As most of these clusters harbor functionally unrelated genes, their colocalization therefore suggests a complex regulatory landscape that potentially arose through mixing of existing individual regulatory interactions (*19*, *22*), preventing separation into their original states.

Different genomic compositions, such as repetitiveness or the chromosome size and composition, affect how much genomic novelty can still be evolutionarily explored within each chromosome. The larger chromosomes are more likely to still harbor unexplored neighborhoods even after hundreds of millions of years after chromosomal element fusion, whereas small chromosomal elements are more likely to exhaust their combinatorial potential. To quantify how much modern genomes can be represented by both chromosomal and subchromosomal mixed states, we next describe a topological approach to this problem.

### Topologically mixed states that define distinct animal genome evolutionary trajectories

Comparative genomics analyses typically examine one genomic scale at a time. However, simultaneously studying FWM and other chromosomal changes requires integrating all genomic scales and features within a single framework (*33*)—from single genes, microsyntenies, and small structural changes to whole chromosome composition and organization (fig. S6). Each level evolves under distinct meiotic, gene regulatory, or functional constraints.

To address this, we implemented an EGT approach, which allows us to view genomes and FWM processes on chromosomes as topological operations (Supplementary Text). In this work, we quantify EGT by calculating pairwise distances between BCnS orthologs. More generally, this approach can be extended to any homologous features in genomes, such as genes, conserved noncoding elements, or repeat families and their insertions (see the “Evolutionary topology methods” section). This allows us to implement two topological approaches as a part of the EGT framework. The first approach compares the topology of many genomes simultaneously, which we term “multigenome topology” (MGT), and allows the analyses of evolutionary trajectories across species, haplotypes, or cell structures. In the MGT implementation for this manuscript, each sample is a single genome or haplotype. While plotting, each sample is a single point. Each sample is encoded as a vector of interlocus base pair distances of BCnS ALG orthologs, representing every pairwise locus distance. The second approach, which we call “multilocus topology” (MLT), allows the simultaneous analysis of the topology evolution of loci within and between clades of genomes. In the MLT analyses, the interaction values for pairs of homologous loci are phylogenetically averaged, while the MGT uses distance measures from individual genomes (see the “Evolutionary topology methods” section). MLT enables comparison of changes across a clade, such as colocalizations of loci, larger chromosomal changes (*2*), and detection of new or lost gene linkages. In the MLT implementation for this manuscript, each sample is an orthologous locus shared across species, and, when visualized, each locus’ averaged value across the clade is represented by a single point.

Dimensionality reduction and projection methods, such as uniform manifold approximation and projection (UMAP) (*53*) or t-distributed stochastic neighbor embedding (t-SNE) (*54*), are then used to visualize the interaction matrix of whole-chromosome differences between genomes or the within-genome topology of single or multiple genomes (see the “Evolutionary topology methods” section). Eigenvector and singular value decomposition approaches are the key concepts for FWM generalization across genomic scales of organization, as they can be used as a measure of mixing (Supplementary Text). Raw orthology information and positional data can be stored and processed with graph database approaches (see the “Graph database integration” section and fig. S6) for the downstream dimensionality and manifold projection approaches.

**Fig. 4.**
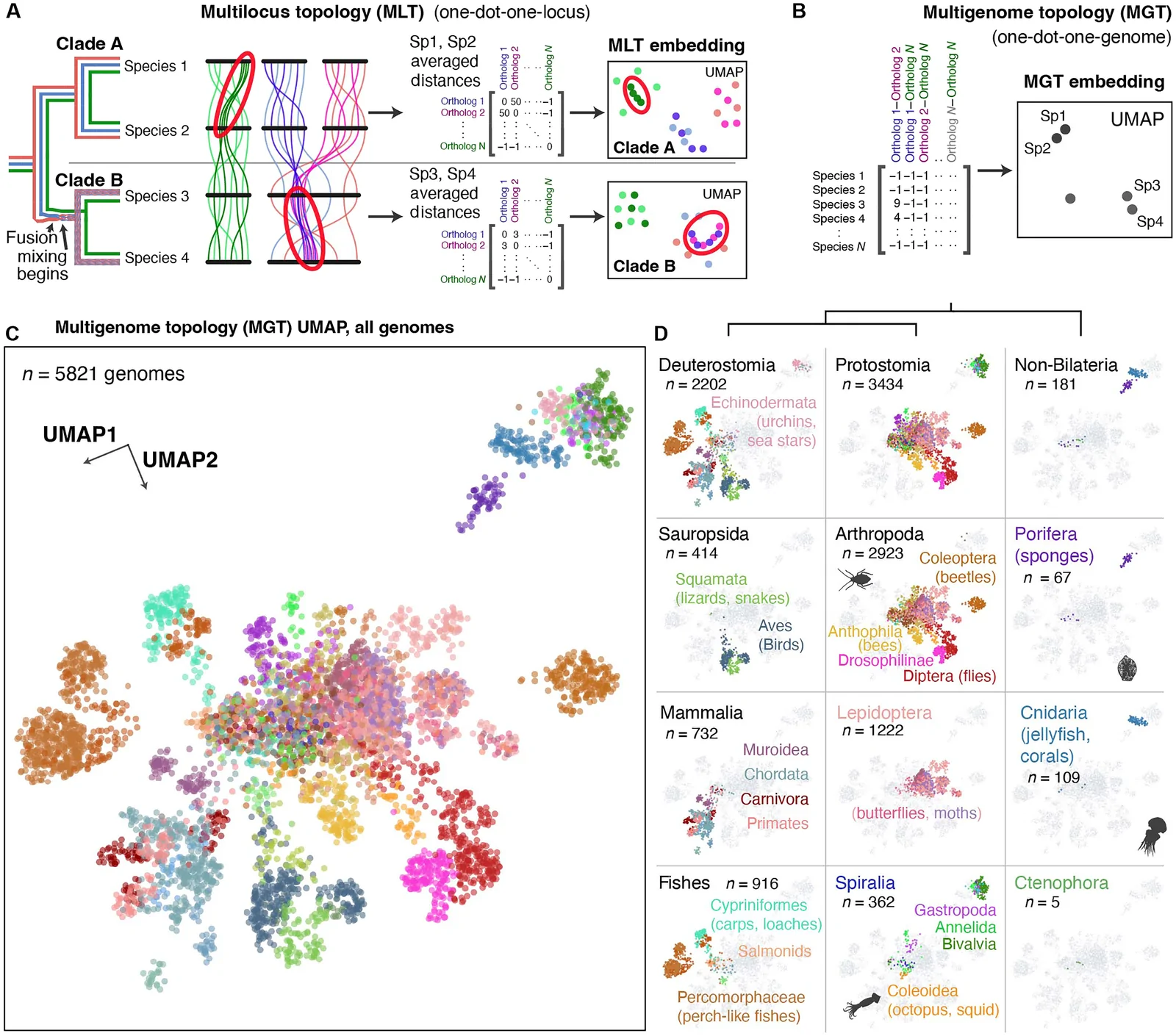
FWM representation on topological spaces. (**A**) MLT: Mixing dynamics and gene colocalization can be approximated by averaged pairwise distances between orthogroups in each genome for a given clade to produce manifold projects for each clade. This distance matrix is then parsed through a manifold reduction algorithm such as UMAP to visualize orthologous regions that are more prone to mixing. Specifically, FWM of chromosomes will result in their orthogroups to be inseparable on the UMAP. Similar observations can be made for mixed subchromosomal regions (red oval in clade A UMAP). (**B**) MGT: Orthogroup pair distances across species can further be used to identify species with similar colocalization distributions. (**C**) A MGT projection of 5821 chromosome-scale animal genomes (*n* = 4454 species), in which each dot represents one genome. We use the genes from the previously defined BCnS ALGs as anchors in the genomes of all chromosome-scale genomes, showing distinct colocalizations that are often clade specific. (**D**) The 12 smaller plots depict the same UMAP; however, each plot only contains colored dots for genomes from the labeled clade. Gray dots in these small plots are from genomes from species outside the labeled clade.

The MGT manifold projection on the pan-metazoan dataset (Fig. 4C and figs. S7 and S8) reflects species grouping by phylogenetic signal. Coloring clades within animals, such as classes or orders, reveals that genomes of closely related species cluster after dimensionality reduction. Observing clades with many species, such as Chordata or Lepidoptera, reveals that there are subclusters in UMAP space that reflect their phylogenetic relationships (Fig. 4C and file S1). We next performed three tests to verify that phylogenetic clustering in MGT space was stable regardless of phylogenetic sampling, dimensionality reduction parameters, and representation of missing locus pairs.

The MGT analyses were robust to variable phylogenetic sampling. To test whether heavy sampling of chordates (*n* = 2147) and arthropods (*n* = 2923) in our dataset biased MGT clustering, we implemented a taxonomic resampling scheme that caps species per rank while maximizing taxonomic diversity (Materials and Methods). Across resampling from different taxonomic ranks, i.e., genus to phylum levels, genomes still clustered phylogenetically—even with limited sampling of 10 genomes per phylum (fig. S8). It may therefore be preferable to include all genomes in the MGT analysis, using taxonomic resampling in cases such as plotting genomes from a single clade. We next assessed sensitivity to dimensionality reduction parameters (*53*). Sweeping UMAP neighbors (≤250) and minimum distance (≤1.0) showed that MGT’s phylogenetic clustering is stable regardless of parameter choice (fig. S7). Smaller neighbor values tended to sharpen clustering at lower taxonomic ranks. Last, we evaluated how encoding noncolocalized pairs affects MGT. Interlocus distances exist only for genes on the same chromosome (fig. S9A); therefore, when genes are on different chromosomes, entries are missing and must be filled with a sentinel distance for dimensionality reduction (fig. S9B). To prevent absence from being mistaken for a real distance, the sentinel should exceed any true interlocus distance, so we used 1 trillion base pairs, approximately one order of magnitude longer than the longest real chromosome in the dataset. Results were similar for sentinel values down to 100 Mb. Below that threshold, absences start entering each sample’s nearest-neighbor graph and erode phylogenetic resolution (fig. S9C). This shows that both the real base pair distances and a consistently “far” encoding of absence drive MGT’s phylogenetic clustering.

**Fig. 5.**
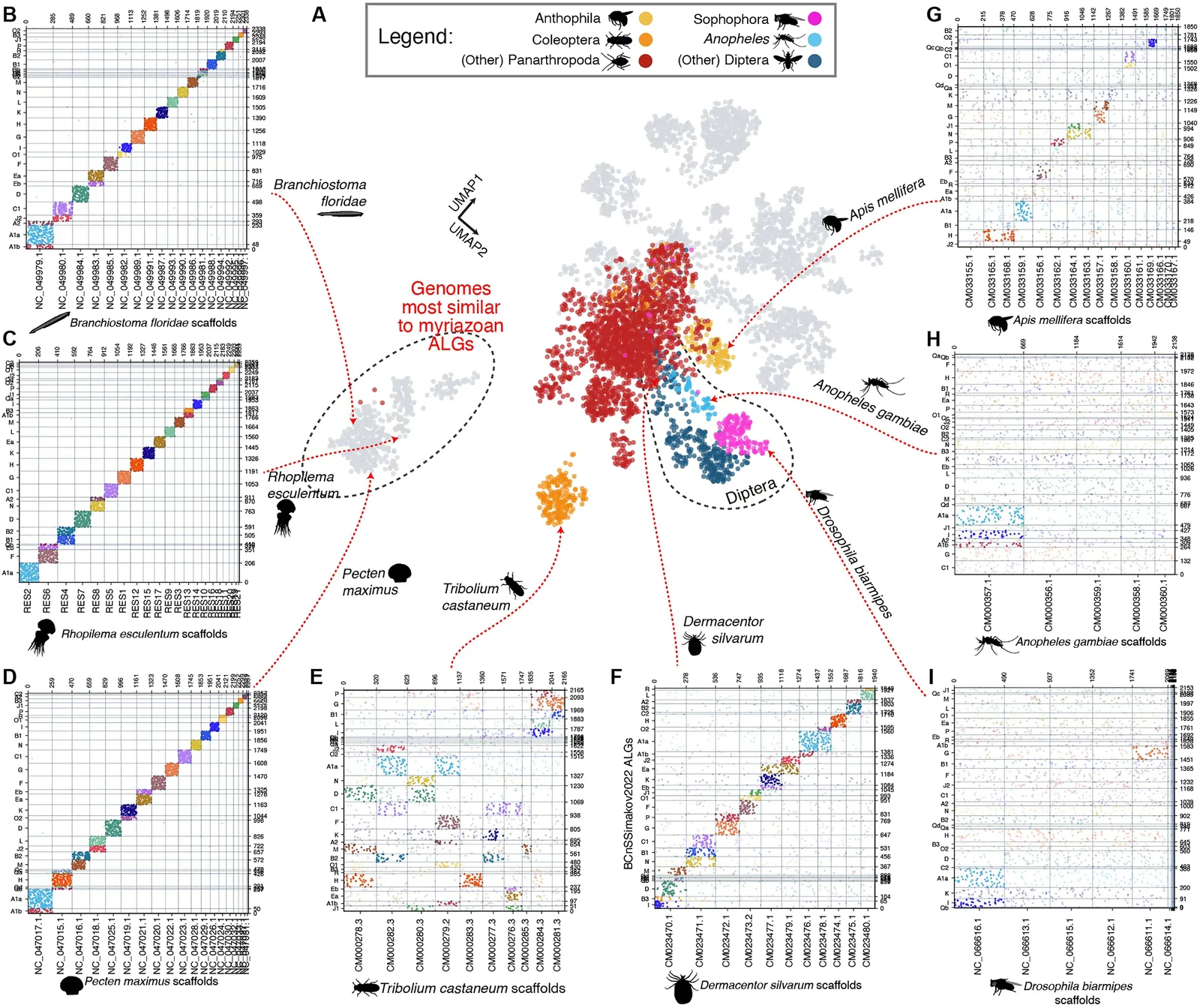
MGT reflects genome evolutionary changes. (**A**) UMAP from (Fig. 4C) (*n* = 5821 genomes), with species within Arthropoda highlighted. The genomes with the fewest major chromosomal rearrangements changes since the ancestral myriazoan genome are circled and labeled. Genomes outside of this circle have varying degrees of major chromosomal rearrangements from the ancestral BCnS ALGs. A clade with highly derived genomes, the Diptera, is also circled. Most chromosomes within the cluster of the least derived genomes contain single ALGs, i.e., (**B**) *Branchiostoma floridae*, (**C**) *Rhopilema esculentum*, and (**D**) *P. maximus*. (**E**) From the other cluster in the UMAP mass, there are both genomes with significant conservation of BCnS ALGs (*P* < 0.05, one-sided Bonferroni-corrected Fisher’s exact test), such as in beetles (Coleoptera, *Tribolium castaneum*) and (**F**) the tick *Dermacentor silvarum.* Well-represented clades with lineage-specific genomic changes form clusters and lobes, such as (**G**) bees (Anthophila, *Apis mellifera*) and Diptera. The MGT also demonstrates how genomes with similar highly reduced karyotypes can be discerned, as seen in (**H**) mosquitoes (*Anopheles gambiae*) and (**I**) drosophilid flies (Sophophora, *Drosophila biarmipes*).

MGT analysis reveals a potential “start” of the evolutionary trajectory as a cluster of some representatives from mollusks, annelids, sponges, cephalochordates (i.e., amphioxus), and cnidarians (Figs. 4C and 5, A to D, and table S1)—clades that were previously identified as having few interchromosomal rearrangements and from which the BCnS ALGs were inferred (*2*). Cnidaria and Porifera form their own subclusters within this larger cluster. The Porifera genomes form a trajectory projecting to the cluster containing all other animal genomes. The separation of Cnidaria from the rest of the BCnS ALG–like genomes, as well as the trajectory of Porifera genomes extending toward all other animal genomes, is likely because of the shared, lineage-specific chromosomal fusions present in both of these clades.

Patterns in the MGT embedding can coincide with variation in genomic features, but interpreting these associations requires accounting for phylogenetic structure. Mapping genome size distribution (fig. S10, genome_size_log10) reveals an apparent genome-size gradient across the MGT UMAP, largely explained by clade membership. In particular, the region enriched for larger genomes is dominated by deuterostomes, whereas other large-genome lineages (for example, cephalopods) occupy UMAP regions otherwise composed mainly of species with smaller genomes. Thus, genome size does not act as an independent driver of global placement in the embedding.

MGT analysis allows us to trace evolutionary trajectories in many other metazoan clades. Several radiating trajectories can be assigned to phylogenetic groups (color-coded), such as Chordata, with further phylogenetically branching subprojections extending into the UMAP space (Fig. 4D); Diptera, with mosquitoes forming a bridge toward seemingly more-derived genomes of drosophilid flies; Hymenoptera; *Schistosoma* species; and several families of Lepidoptera, to name a few (Fig. 4C). The genomes of many species in these clades have undergone moderate interchromosomal rearrangements since the myriazoan ancestors, such as the chordates, beetles, teleost fish, dragonflies, felids, and hexapods. Conversely, some of the clades, such as Diptera, *Schistosoma* species, and Nematoda, lie toward the end of these trajectories but have undergone extensive rearrangements since the myriazoan ancestor. These distinct projections are linked by a central hub of species with moderate levels of chromosome rearrangements (Fig. 5E), reflecting that their genomes are less derived in terms of BCnS ALG composition and mixing. For example, the genomes of a tick (*Dermacentor silvarum*), a beetle (*Tribolium castaneum*), mosquitoes, and fruit flies lie on a continuum of genomes with varying amounts of rearrangements (Fig. 5H).

We next tested whether convergence, or homoplasy, in our EGT implementation could produce a “long-branch attraction” analog in MGT space. If extensive rearrangement led to a generic end point, then distantly related lineages with highly scrambled genomes (for example, clitellate annelids and mosquitoes) should collapse into the same region of the embedding. Instead, across substantially different UMAP parameterizations (e.g., *n* = 20, *m* = 0.75 and *n* = 150, *m* = 0.75; fig. S11), derived clades remain largely nonoverlapping and occupy distinct regions. Some clades become adjacent under higher-neighbor settings, but they do not merge into a single convergent cluster, and these adjacencies are not stable across parameter choices. Consistent with this, mosquitoes, drosophilid flies, nematodes, clitellate annelids, ctenophores, and cephalopods—despite all being extensively rearranged (*3*, *9*–*15*)—do not consistently neighbor one another across neighborhood sizes or distance metrics. These results show that radical genome rearrangements do not converge on similar whole-genome topologies, reflecting different impacts of dissociation and consolidation processes. Additionally, there is still meaningful information in comparisons between highly rearranged genomes and related species without extensive rearrangements. For example, although *Drosophila* has four derived pairs of chromosomes and *Caenorhabditis* has five (consolidation), their chromosomal compositions produce distinct positioning on the MGT, reflecting clade-specific rearrangement history.

**Fig. 6.**
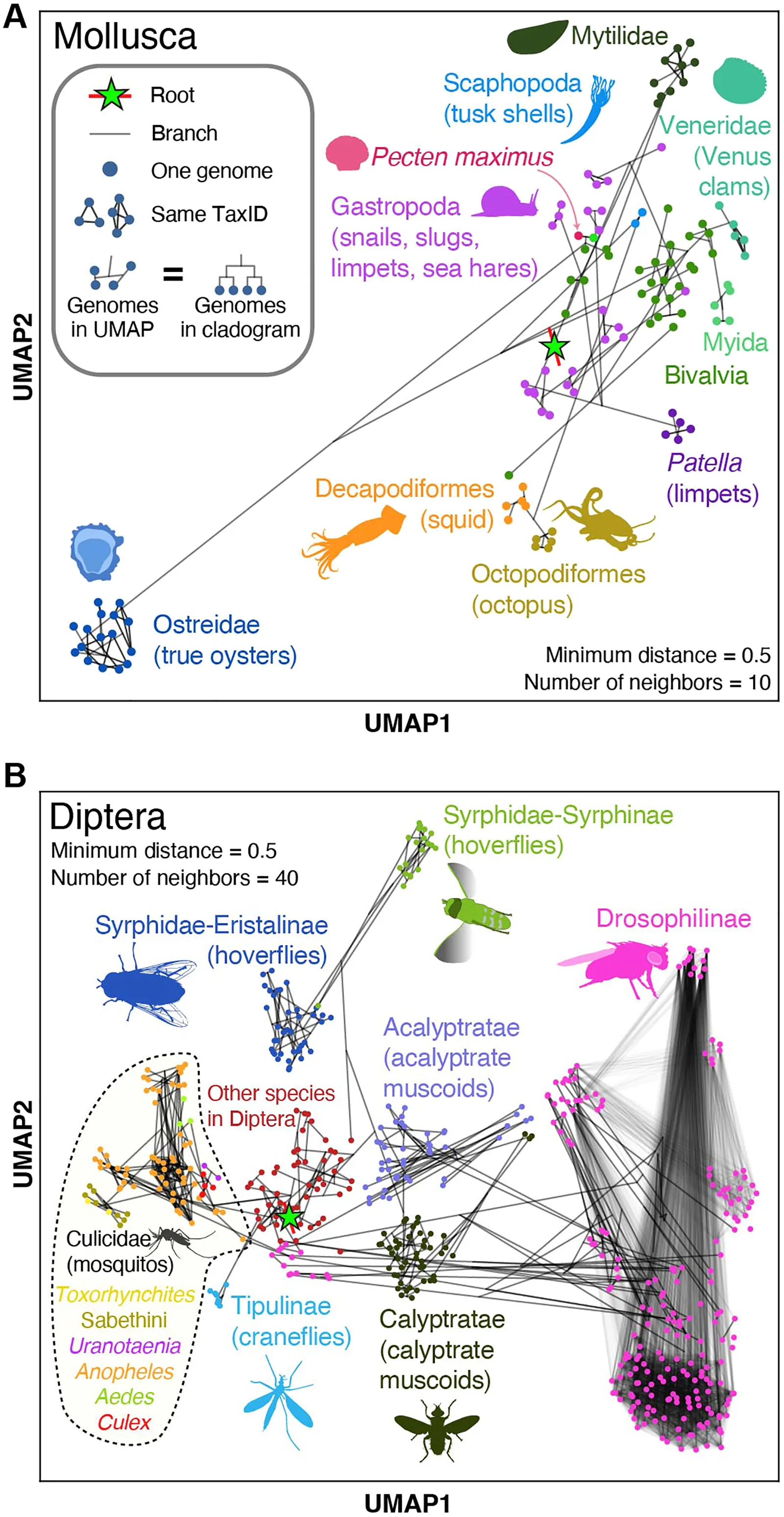
MGT reflects genome phylogenetic changes. Both panels are UMAP embeddings of clades within the all-animal dataset. Individual genomes (dots) are colored according to phylogeny, and a cladogram is drawn, such that branches between genomes and ancestral nodes are gray lines. (**A**) A re-embedding of mollusk genomes (*n* = 108 genomes, 80 species) captures major phylogenetic transitions. Lineage-specific divergences from the ancestral spiralian genome, such as the karyotype reductions in oysters and limpets, can be seen as individual clusters of closely related genomes. Furthermore, lineage-specific changes in squids and octopus since the coleoid cephalopod ancestor are also captured by this approach. (**B**) Re-embedding of Diptera genomes (*n* = 509 genomes subsetted to 275 species), circled in Fig. 5. Major dipteran clades colocalize in UMAP space, capturing lineage-specific genomic changes.

The different trajectories within even closely related species are exemplified by applying the MGT approach to individual animal clades (Fig. 6). Closely related species, even after tens of millions of years of divergence, occupy close positions in the UMAP space (Fig. 6A). True oysters (*n* = 17 genomes, 8 species), which have undergone consolidation to a reduced karyotype of 1*n* = 10, form a cluster distant from the other mollusk genomes (*n* = 245 genomes, 205 species) and other clades with distinct paths to karyotype reduction (limpets: *n* = 8 genomes, 8 species, karyotype of 1*n* = 9; scaphopods: *n* = 2 genomes, 2 species, karyotype of 1*n* = 9), each forming a separate cluster from the others. Similarly, the major clades in Diptera (*n* = 509 genomes, 415 species) form clusters in UMAP space (Fig. 6B). Therefore, since the MGT approach simultaneously incorporates both chromosomal and subchromosomal localization data, it has the potential to be applied to resolve outstanding phylogenetic questions through analysis of gene localization across clades, extending the previous chromosomal-scale FWM approaches (*3*, *55*).

Our MGT findings suggest that once chromosomes accumulate substantial rearrangement and mixing, those changes are essentially permanent: Lineages cannot easily switch to the architectural configuration of another clade nor revert to their ancestral organization. This accumulating and difficult-to-reverse mixing, at both whole-chromosome and subchromosomal scales, may contribute to the distinct evolutionary paths followed by different clades over deep time.

### Within-chromosomal mixing and putatively entangled states

**Fig. 7.**
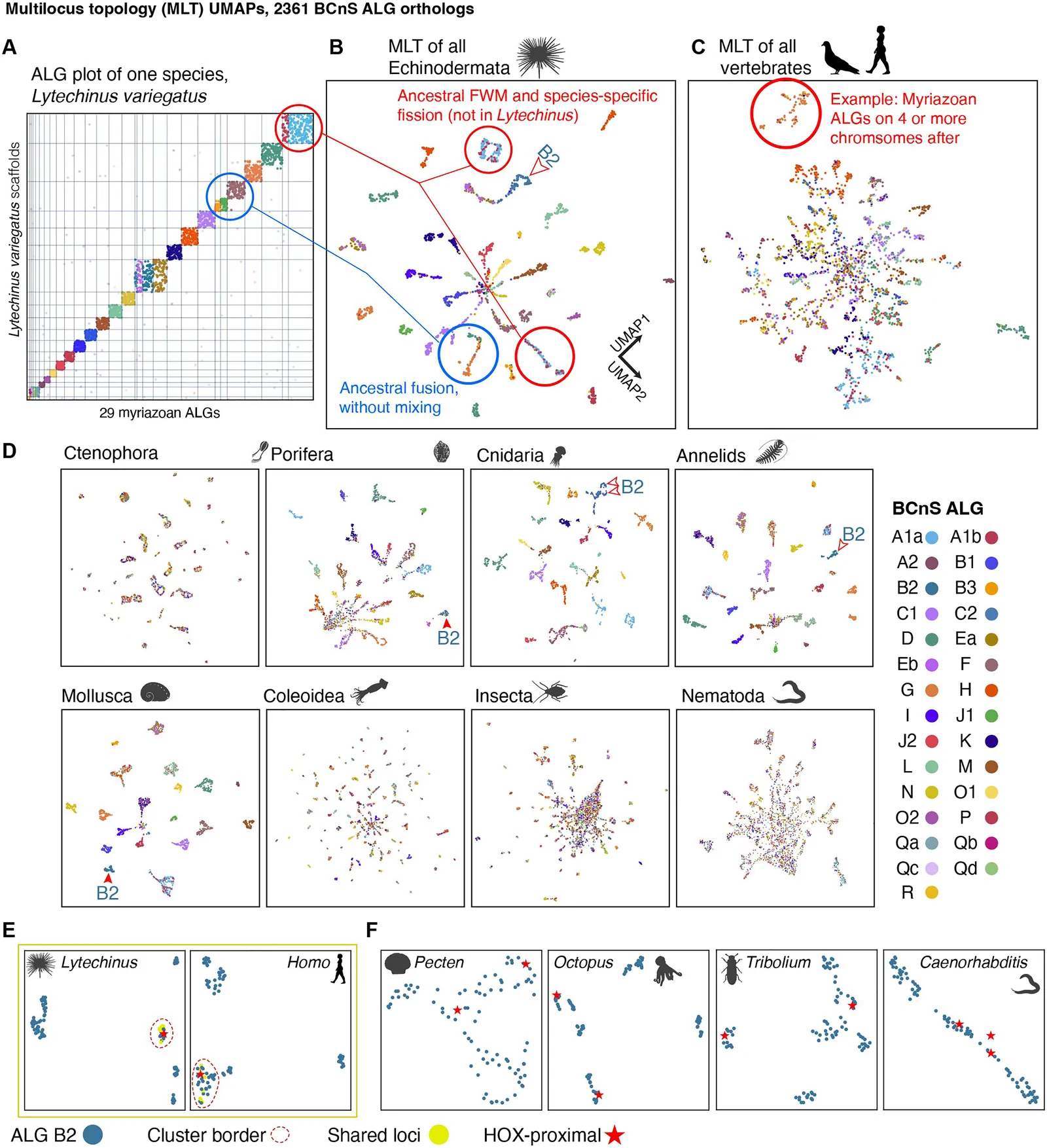
Evolutionary topology of chromosomal elements revealed by MLT. (**A**) An Oxford dot plot of *Lytechinus variegatus* sea urchin chromosomes (*y* axis) plotted against animal ALG gene families (*x* axis) (*2*). Gene families in significant syntenic relationships are solid-opacity dots (Fisher’s exact test, *P* ≤ 0.05). (**B**) A UMAP projection of MLT distances for echinoderm genomes. Each point is one orthologous protein present across echinoderms (*n* = 2361 BCnS ALG orthologs). The red circles show the effect of FWM and subsequent species-specific evolution. The blue circles show the effect of a chromosome fusion event that has not undergone subsequent mixing, and the separation of these ALGs into lobes in the MLT plot shows that this chromosome configuration is conserved across echinoderms. (**C**) MLT plots reveal dramatic chromosome restructuring events. The MLT approach on vertebrate genomes shows that the ALGs are predictably distributed in four or more clusters, reflecting the two rounds of whole-genome duplication in vertebrates since the animal ancestor. (**D**) Plots of additional animal clades show which clades have stable karyotypes and which have diverse chromosomal rearrangements. (**E**) Subchromosomal topology around the Hox cluster for BCnS ALG B2. Blue points are ALG B2 orthologs. Red stars mark Hox-proximal genes. Dashed ovals outline cluster borders, and yellow points indicate loci shared between the two species panels shown (the urchin *Lytechinus* and human, respectively). These local embeddings identify a consistent set of comixed B2 orthologs around Hox across deuterostomes. (**F**) Chromosome-level neighborhoods around the Hox cluster in representative species from other clades: *P. maximus* genes in the context of Mollusca genomes, *O. vulgaris* genes in the context of Coleoidea genomes, *T. castaneum* genes in the context of Coleoptera genomes, and *Caenorhabditis elegans* genes in the context of rhabditid nematode genomes.

To assess the degree and stability of subchromosomal mixing, we applied the MLT approach across multiple genomes within specific clades (Fig. 7). Using phylogenetically weighted locus pair distance matrices (see the “Evolutionary topology methods” section), we computed subchromosomal, whole-genome (Fig. 7B, C, D), and individual ALG UMAPs (Fig. 7E, F). Homologous chromosomes formed distinct regions, appearing as separate entities in the manifold projection, while local feature clusters (conserved linkages) emerged within each chromosome.

The MLT manifold representation also recovers known FWM events, such as the A2 ⊗ N and A1b ⊗ B3 in cnidarians (Fig. 7D, Cnidaria), compared to clades without these fusions, such as noncephalopod mollusks (Fig. 7D, Mollusca). It also allows for assessing the degree of FWM. For example, the cnidarian A2 ⊗ N ancestral FWM appears highly mixed (Fig. 7D, Cnidaria), while the poor mixing between B2 and Ea ⊗ Eb in echinoderms (*n* = 21 genomes, 18 species) is evident (Fig. 7B, Echinodermata). Thus, the MLT manifold representation directly reflects τ_s_ measures and the mixing properties of a given locus. To augment the utility of the manifold representation in identifying clusters of colocalized genes, we sought to quantify locus pairs with clade-specific configurations.

Using the full dataset of animal genomes, we identified clade-defining locus pairs whose subchromosomal interlocus distances are diagnostic for a clade. This locus pair detection is positional: The same stable patterns can arise from regulatory constraint or other selection pressure, or remain through neutral persistence.

For each clade, we score three variables for locus pairs: (a) unique to the clade—the loci share a chromosome only within that clade and are on separate chromosomes in other species; (b) stable within the clade—pairs with low interlocus distance variance retained despite extensive inter- and intrachromosomal changes, while outside the clade the distance varies widely or the loci occasionally fall on different chromosomes; and (c) stable and close within the clade—a subset of stable pairs with small interlocus distance. The stable-and-close clade-specific pairs are microsyntenic and likely reflect cis-constraints and can be identified from both chromosome- and nonchromosome-scale genomes (*19*, *43*, *52*).

**Fig. 8.**
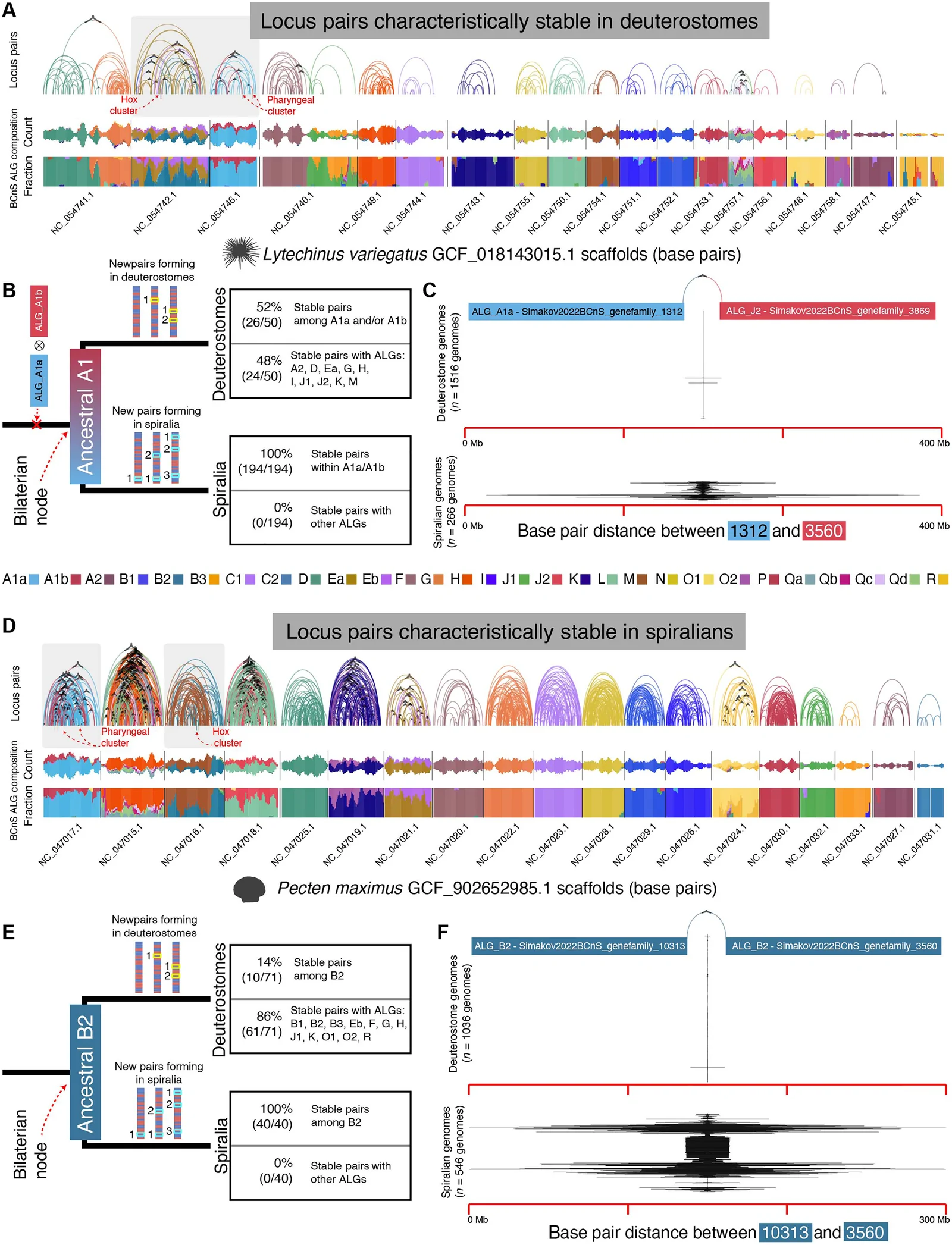
EGT captures genome-defining features. (**A**) The chromosomes of the urchin *L. variegatus*. The bottom row is the fraction of BCnS ALG gene identities along the chromosome. The middle row is the absolute count of BCnS ALG genes along the chromosomes. The colorful arcs on the top row are the pairs of BCnS ALG that are characteristically stable in deuterostomes. Arcs with a black peak on top are stable pairs in which the two loci are from different BCnS ALGs. (**B**) After ALG A1a and A1b fused onto one chromosome before the ancestor of the Bilateria, the evolutionary history of the ALGs progressed differently in the deuterostomes versus spiralians. (**C**) The distance between one locus pair that is characteristically stable in the deuterostomes. The loci are tightly linked in deuterostome genomes but vary in genomic distance widely in nondeuterostome genomes. (**D**) Locus pairs that are characteristically stable in spiralians, plotted in the context of the scallop *P. maximus*. (**E**) There are more inter-ALG stable pairs that have formed in deuterostomes with BCnS ALG B2, the ALG associated with the Hox cluster, versus the spiralians. (**F**) New characteristically stable pairs can form in lineage-specific ways, even from loci that were present on the same chromosome in the ancestral organism. This is a B2-B2 pair that became characteristically stable in deuterostomes but is highly variable in spiralians.

Each clade harbors numerous stable and/or close pairs unique to that clade. In Spiralia, for example, there are 2398 stable pairs and 2471 especially close pairs (tables S15 and S16). Using conservative thresholds (one-sided μ-2σ of distance variance and presence in ≥50% of species; Materials and Methods), the most stable pairs tend also to be close, and these novel associations are rarely lost once formed (Fig. 8, C and F). We find that 690 stable pairs are shared in deuterostomes and 185 are shared in protostomes, with no overlap between the two sets. Within Spiralia, 1743 of the 2398 stable pairs are retained in mollusks, and 610 are retained in annelids, with only 497 shared between mollusks and annelids. Ancient clade-specific pairs are rare (Bilateria, Parahoxozoa, etc.), reflecting dispersal and convergent colocalizations across metazoan evolution. These clade-specific stable and/or close locus pairs, regardless of how they have persisted, contain phylogenetic signal in that they hierarchically group clades by chromosome structural similarity (Figs. 1 and 4 to 6). This pattern suggests that subchromosomal reorganization, rather than large-scale karyotype change, plays a key role in shaping clade-specific chromosomal architecture, with persistent novel subchromosomal linkages preserved against the backdrop of highly derived mixed chromosomes (*m* ≈ 1 from the ancestral BCnS ALGs) (Fig. 1).

Gene Ontology (GO) term analyses of clade-specific stable and close pairs revealed enrichment for biologically coherent categories that often track clade biology (see Materials and Methods, Supplementary Text, fig. S12, and table S17), suggesting that the maintained linkages are not random in aggregate. The contributing pairs include both genes that are ancestrally colocated on the same chromosome and genes that ancestrally lay on different chromosomes and have become colocalized through rearrangement. Many clade-specific gene pairs are pervasive in clades but absent outside the clade. As one example, the genes for eukaryotic translation initiation factor 2 subunit 1 and V-type proton adenosine triphosphatase subunit D, on different chromosomes outside vertebrates, are colocalized head-to-head less than 5 thousand base pairs (kb) apart in 90.4% of vertebrate species sampled (*n* = 1910 of 2113) and, in humans, are coregulated by nuclear respiratory factor 1 transcription factor (TF) binding the shared intergenic region (fig. S12 and table S17).

These clade-specific stable pairs span a wide range of interlocus distances. Within Vertebrata, stable pairs are distributed from a small minority that are tightly colocated (≤10 kb head-to-head) to the bulk that are separated by hundreds of kilobases to multiple megabases, with a modal distance near 1 Mb (fig. S13). While the closest locus pairs can resemble bidirectional promoter or genomic regulatory block–type microsyntenies (*19*), most of the stable pairs are long-range colocalizations, indicating that the underlying signal is not simply the recovery of close gene pair microsynteny. Consistent with this, most of these associations do not show signs of coregulation or coexpression and so suggest independent regulation (table S17). These patterns leave open the possibility of multiple distal, positionally mixed regulatory contacts (e.g., enhancer-promoter), imposing constraints that enable long-term maintenance, as documented across animals with differing contributions to regulation (*17*, *56*–*59*), a process we term “entanglement” (*28*). Similar to chromosomal FWM, entanglement can lead to the loss of the ancestrally unmixed, separate state homology information.

Entanglement can facilitate co-option or evolution of new regulatory elements through contacts after chromosomal rearrangements (*28*). For example, among the long-range examples (e.g., in vertebrates, MAP3K20/HAT1 at 780 kb apart or SLC25A26/TMF1 at 2.5 Mb apart), some TFs bind both anchor promoters in pooled human or *Drosophila* chromatin immunoprecipitation sequencing (ChIP-seq) (figs. S13 and S14), leaving open the possibility of evolving coregulation that facilitates further constraint within these loci and preventing their “disentanglement” into what ancestrally might have been separate states. Further functional validation of these loci is required to test these predictions.

Our stable locus pair approach also reveals emergent patterns in stable pair formation that reflect different chromosomal evolutionary histories. For example, BCnS ALG B2 that contains the Hox cluster and BCnS ALG A1 that contains the pharyngeal cluster (*60*) and many homeobox genes (*7*) show contrasting patterns between deuterostomes and spiralians (Fig. 8, B, C, E, and F). In spiralians, both chromosomes exclusively formed novel within-chromosomal stable pairs (BCnS ALGs B2 or A1; Fig. 8, B, E, and F), with A1 forming nearly five times as many novel, spiralian-specific, stable pairs. In contrast, deuterostome chromosomes that carry B2 or A1 also harbor stable pairs involving genes from other ALGs, meaning genes that were not ancestrally on the same chromosome but became colocalized through fusion-and-mixing. The B2-bearing chromosome (which contains the Hox cluster) is especially enriched for these inter-ALG stable pairs: 43 of 50 stable B2 pairs are inter-ALG compared with 307 of 556 among other deuterostome stable pairs (Fisher’s exact test, odds ratio = 4.98, *P* = 8.3 × 10^−6^), suggesting that it has accumulated many novel gene neighbors through postfusion rearrangement.

These differences highlight distinct levels of neighborhood exploration and the possible evolution of novel constraints. In echinoderms, the Hox topological group comprises 17 orthogroups, 7 shared with vertebrates (Fig. 7E). This includes one ALG B2 gene family [gene family 6215 (*2*)] stably linked to a gene resulting from the ALG R dispersal in the chordate lineage [gene family 3739 (*2*)]. Although not known to be functionally related to the Hox cluster, these genes may have become “entangled” into its MLT neighborhood since the chordate ancestor, likely reflecting constraints on neighboring loci or simple persistence of an undisrupted configuration. In contrast, nondeuterostome invertebrates exhibit fragmented MLT neighborhoods around Hox cluster genes, which have been distributed across chromosomes in clades such as mollusks, coleopterans, and nematodes, often with very different sets of newly associated genes (Fig. 7F). Over 500 million years, distinct MLT neighborhoods have evolved around Hox clusters between vertebrates and invertebrates, with prior work attributing the divergence in Hox cluster composition to differences in regulatory architecture (*21*, *43*, *61*).

In summary, our results, currently based on the distances between ortholog groups conserved across animals (BCnS ALGs), describe genome evolution as an iterative process of chromosomal and subchromosomal mixings that progressively and irreversibly erase ancestral states. At the same time, these rearrangements lead to novel, stable intrachromosomal linkages (“entanglements”) that contribute to clade-specific genome architectures and may acquire regulatory significance. Whether the presence of these pairs is driven by selection or they are a result of neutral persistence amidst other rearrangements remains an open question and will likely be species or clade specific, although the clade specificity and long-term stability of many pairs suggest that at least some eventually become functionally constrained.

Because the BCnS ALG set comprises 2361 highly conserved orthogroups, this analysis samples only a subset of ∼25% of genes in a typical bilaterian genome. We note that for a human genome (3.2 billion base pairs), this is one marker per ∼1.5 Mb, and a density of one marker per ∼50 kb for a genome size similar to *Drosophila* (100 Mb). As a result, our inferences emphasize deeply conserved loci and do not consider clade-restricted gene families. Future analyses may apply these approaches with clade-specific loci and other marker types in addition to highly conserved protein-coding genes.

## DISCUSSION

Our analysis of thousands of animal chromosome–scale genomes reveals a multitude of clade-specific, stable subchromosomal configurations. We propose that the irreversibility of chromosomal mixing and the persistence of these mixed genomic states are the main contributing factors to observed genomic macroevolutionary trends (*62*, *63*). Our findings suggest that large-scale karyotypic changes can reshape genomic context over macroevolutionary time, thereby altering the set of regulatory interactions that can potentially arise as chromosomes consolidate, dissociate, and rearrange within chromosomes.

We quantify the history and timescale of chromosomal and subchromosomal evolution across animals and establish baseline rates of chromosomal change across animal history as a foundation for future comparisons to patterns of speciation. Topological approaches in macroevolution (*63*), particularly the EGT framework described here, provide a platform for understanding the evolution of mixing, loci linkage, and irreversibility across genomic scales. These methods, combined with the addition of regulatory data and chromosome-scale genomes from diverse animal lineages (*26*, *27*, *64*, *65*), will further clarify how present-day genome configurations are consequences of evolution’s past and how they constrain and shape possible future evolutionary and genome architecture trajectories.

Furthermore, our findings identify clade-specific locus-pair configurations that persist over deep time, arguing that at least a subset of genome architecture states are constrained once established. While exact mechanisms for this maintenance are unclear, the mechanical consequences of chromosomal mixing and the resulting potential regulatory entanglement emerge as a likely scenario. Testing how functional novelty can arise in these states over larger evolutionary time spans in diverse metazoan model organisms will be critical for further evaluation of whether and how genome architecture translates into regulatory and phenotypic evolution.

## MATERIALS AND METHODS

### Constructing a genome database

We sought to analyze a comprehensive dataset of all chromosome-scale animal genomes available through NCBI. We included multiple assemblies per species where available (including alternate haplotypes) to strengthen ancestral inference, to corroborate results against different assembly and annotation types, to capture meaningful intraspecific structural variation, and to increase robustness to assembly-specific errors. The scripts to build the database are available in the chrombase and genbargo GitHub repositories written for this manuscript (see the “Data, code, and materials availability” section). This software requires Snakemake v.9.9.0+ (*66*), ETE4 v.4.3.0+ (*67*), NCBI Datasets v.18.6.0+ (*68*), Fastapy v.1.0.3+ (*69*), miniprot v.0.18+ (*70*), Pandas v.2.1.4+ (*71*, *72*), and odp v.0.3.3+ (*3*). All downstream chromosome evolution, tree, MGT/MLT, clade-defining locus pairs, chromosomal change time-domain analyses, and GO analyses are now packaged as subcommands of the egt software, written for this manuscript, and presented as a reproducible pipeline in the schultz-et-al-2026 repository (see the “Data, code, and materials availability” section).

To build the chromosome-scale genome database, the Snakemake workflow chrombase_scrape_genomes_NCBI.snakefile was used to query NCBI datasets for all historical and contemporary assemblies and the latest annotations. To retain only high-quality records, we kept genomes annotated as chromosome scale on NCBI and removed deprecated entries. Annotated genomes were selected first, and to avoid duplicates, unannotated GenBank versions of those assemblies were discarded. Unannotated human genome assemblies were also removed. Nonchromosome-scale assemblies were also cataloged by the pipeline but excluded from downstream analysis. We excluded assemblies currently under embargo, following the embargo policies of major projects and institutes (Vertebrate Genomes Project, the Bat1K project, the European Reference Genome Atlas, the Genome 10K project, the Bird 10K project, the Human Pangenome Reference Consortium, the Telomere-to-Telomere Consortium, the Leibniz Institute for Zoo and Wildlife Research, the DNA Zoo, and the Wellcome Sanger Institute). Embargo dates were calculated within the workflow using filter_assemblies.py from the genbargo tool commit e420471. The list of assemblies used here, with assembly metadata and embargo information, is available in table S1.

The database of annotated genomes was built with the Snakemake workflow chrombase_build_db_annotated_chr.snakefile. For each assembly, only chromosome-scale scaffolds were downloaded by parsing NCBI Datasets output and omitting scaffolds marked as Mitochondrion, unplaced-scaffold, alt-scaffold, unlocalized-scaffold, or Un. Genome annotations and protein FASTA files were downloaded with NCBI Datasets, and annotations were converted to .chrom files with the NCBIgff2chrom.py helper in chrombase. Taxonomic information from the NCBI Taxonomy database (*73*, *74*) was recorded for each assembly using ETE4 v.4.3.0+ (*67*). The workflow then generated a config.yaml file with file paths and assembly metadata for the downstream reciprocal best-hit (RBH) and egt analyses. Chromosome-scale but unannotated assemblies were processed with chrombase_build_db_unannotated_chr.snakefile, which mapped the 2361 BCnS protein families to chromosomes with miniprot and converted the best-scoring mapping for each BCnS ALG ortholog to .chrom format.

Phyla, classes, and orders were counted using NCBI classification. We used a list of 32 phyla consistent with recent literature (*75*): Annelida, Arthropoda, Brachiopoda, Bryozoa, Chaetognatha, Chordata, Cnidaria, Ctenophora, Cycliophora, Echinodermata, Entoprocta, Gastrotricha, Gnathostomulida, Hemichordata, Kinorhyncha, Loricifera, Micrognathozoa, Mollusca, Nematoda, Nematomorpha, Nemertea, Onychophora, Orthonectida, Phoronida, Placozoa, Platyhelminthes, Porifera, Priapulida, Rotifera, Sipuncula, Tardigrada, and Xenacoelomorpha. Phyla from Ruggiero *et al.* (*75*) excluded from the above list are Acanthocephala (*76*) and Rhombozoa (*77*). Recognized classes and orders were collected using the application programming interface of the Global Biodiversity Information Facility.

The full list of genomes used in this study, as well as the percent of genomes contributed by submitter, is in table S1. We recognize the substantial investments made by genome sequencing consortia and individual laboratories in producing the chromosome-scale genomes analyzed in this study. Below, we briefly note the contributors of 0.75% or more of the total genomes to our dataset based on NCBI “Assembly Submitter”: The Wellcome Sanger Institute (previously known as “The Sanger Centre” and “Wellcome Trust Sanger Institute”; 2655 genomes or 45.62% of genomes in the dataset), Genoscope CEA (117, 2.0%), the Vertebrate Genomes Project (108, 1.86%), Northwest A&F University (83 genomes, 1.43%), the DNA Zoo (52 genomes, 0.89%), and the US Department of Agriculture (45, 0.77%). Additional institutions contributing more than 20 genomes include Zhejiang University; China Agricultural University; Centro Nacional de Análisis Genómico; Institute of Oceanology, Chinese Academy of Sciences; Institut de Biologia Evolutiva Consell Superior d’Investigacions Científiques-Universitat Pompeu Fabra (CSIC-UPF); University of Cambridge; Nanjing Agricultural University; European Molecular Biology Laboratory’s European Bioinformatics Institute (EMBL-EBI), Wellcome Trust Genome Campus, Cambridge; Chinese Academy of Agricultural Sciences; The Max Planck Institute of Molecular Cell Biology and Genetics; Bat1K Project; Southwest University; GenoFish; Ocean University of China; Princeton University; Rothamsted Research; and Huazhong Agricultural University. The remaining 39.23% of genomes in the dataset were submitted to NCBI by contributors with 20 or fewer chromosome-scale genome uploads after filtering.

We acknowledge the substantial resources, collaboration, and long-term commitment required for large-scale genome sequencing and appreciate the efforts of all contributing institutions and individuals whose work was invaluable to this study. We also recognize the pioneering efforts of those who developed the sequencing and assembly technologies and methodologies that have made chromosome-scale genome reconstruction possible.

### Homology detection audit

To assess whether per-clade dispersal estimates could be inflated by homology detection failure, we computed the fraction of the 2361 BCnS gene families (*2*) recovered in each of the 1037 species with an NCBI annotation. Each species’ proteome was searched against the BCnS ALG reference catalog via the odp v.0.3.7 pipeline (*3*) using DIAMOND blastp v.2.1.3 (*78*) with standard reciprocal best-hit identification. Per-species median percent identity to BCnS consensus sequences was taken from the existing divergence_vs_dispersal analysis; dispersal and conservation scores (1 − dispersal) were computed as described in the “Dispersal characterization” section. Pearson and Spearman correlations were computed cohort-wide and per clade (*n* ≥ 5 species) using Pandas v.2.1.4 (*71*, *72*), and SciPy v.1.12.0 (*79*).

### Balanced phylogenetic sampling

For some analyses, we downsampled chromosome-scale genomes to test the effects of balanced coverage across the animal tree. The current implementation is egt phylotreeumap-subsample and the companion Snakemake workflow workflows/phylotree_umap_subsampling.smk. The workflow uses NCBI taxonomy (*73*, *74*) through ETE4 v.4.3.0+ (*67*) to place assemblies into taxonomic buckets and enforces a per-bucket cap (max_per_bucket). Within each bucket, priority species (human, mouse, and *Drosophila melanogaster* by default) are added first, preferring RefSeq over GenBank. Remaining slots are filled to maximize phylogenetic spread by iteratively choosing assemblies with the greatest taxonomic path distance from those already selected. The rank hierarchy, random seed, and optional priority NCBI taxonomy identifiers (taxids) are configurable.

### Identifying BCnS ALGs

Per-species RBH files remain the upstream input generated by odp rather than by egt. We used odp to compare the ancestral BCnS ALGs to chromosome-scale genomes of extant animals. In the odp config.yaml, ignore_autobreaks was set to true, diamond_or_blastp to diamond, duplicate_proteins to pass, plot_LGs to true, and plot_sp_sp to false. The plot_LGs mode uses HMM models of each BCnS ALG ortholog to find the best match in each annotated proteome. HMM searches used HMMER hmmsearch v.3.4 (*80*) with an inclusion cutoff of 1 × 10^−5^. For unannotated genomes, HMM search was not repeated because the chrombase workflow had already annotated BCnS ALG ortholog coordinates with miniprot. The resulting *P* values for conservation between BCnS ALGs and extant chromosomes were calculated with Bonferroni-corrected one-sided Fisher’s exact tests and stored in .rbh files consumed by egt.

### Inferring ALG evolutionary history

To quantify chromosomal changes across evolutionary time, we constructed a time-calibrated taxonomic tree with egt taxids-to-newick and egt newick-to-common-ancestors. The topology follows NCBI taxonomy (*73*, *74*) using ETE4 v.4.3.0+ (*67*), with ctenophores placed sister to other animals following Schultz *et al.* (*3*), and node ages were calibrated from TimeTree divergence estimates (*81*). For species absent from TimeTree, we used the closest available relative with a time estimate. The egt newick-to-common-ancestors stage emits the calibrated Newick tree plus node, edge, and divergence time tables used by downstream egt stages.

First, we calculated basic statistics about the presence or absence of BCnS ALGs in extant genomes. One genome for each NCBI taxid was selected to represent each species in the dataset. Significant BCnS ALG–chromosome combinations were selected from .rbh files using a Bonferroni-corrected one-sided Fisher’s exact test threshold of *P* ≤ 0.05. ALG pairs were scored as colocalized when both ALGs were significant and present on the same scaffold. These steps are now implemented by egt alg-fusions, which produces perspchrom.tsv, per_species_ALG_presence_fusions.tsv, locdf.tsv, tree1.tsv.gz, changestring checkpoints, and a summary of clade-specific changes. The reproducible wrapper invokes this stage in pipeline/step4_persp_chr/run.sh.

Using the same program, we next inferred the history of animal chromosome evolution from the BCnS ALGs using a lineage-aware algorithm (*n* = 5821 genomes). Briefly, the program works by reducing the information about BCnS ALG localization in extant genomes from the .rbh files into a matrix that encodes whether two BCnS ALGs are colocalized on the same chromosome in that genome. After matrix construction, for each genome, the program performs a depth-first search from tip (species) to the animal root and infers the branch along which specific chromosome fusion or loss through dispersal events occurred. The pseudo-code in Algorithm 1 is as follows:

**Algorithm 1.** For each species, this algorithm traverses the NCBI taxonomy from tip to the animal root, comparing ALG presence and colocalization against each successive sister clade to assign every inferred fusion and loss through dispersal to a specific branch. LTD, loss through dispersal.

(1) *d*: DataFrame with information about ALG presence and colocalization on single chromosomes.

(2) *t*: NCBI taxid string for a species in *d*.

(3) *n*: Node representing the inference point for ALGs. Can be a species or ancestral node.

(4) *min_M_*: Threshold for determining missing ALGs. We use 0.8. This means that 80% of the genomes being considered must be missing this ALG to consider it lost from that species set. This value below 100% allows for the detection of ALGs that are still present but difficult to detect because of the evolutionary history of the chromosomes in that clade.

(5) *min_N_*: Threshold for determining noncolocalized ALG pairs. We use 0.5. This means that if 50% of the species in this dataset do not have an ALG pair colocalized on the same chromosome, we mark that the ALGs are not colocalized on the branch leading up to the most basal shared node for this clade. If ALGs are colocalized on the same chromosome in the branch leading up to a clade, then we would expect to find the colocalization in 100% of species. However, because of dispersal or the limitations of Fisher’s exact test in genomes with few chromosomes, it is possible that using a cutoff of 100% will produce false negatives for ALG colocalizations. Therefore, a cutoff below 100% is necessary to find colocalizations. Because the algorithm below polarizes observed colocalizations in one species against a sister clade, it instead measures the percent of species in the sister clade that do not contain the ALG colocalization in question. If convergent fusions were not possible, then we would optimally test that 0% of species in the sister clade contain the ALG fusion in question to locate the branch on which the fusion occurred. However, the algorithm must accommodate the possibility of convergent fusions, lineage-specific fusions, the effects of dispersal, and the unequal sampling across the phylogenetic tree. For these reasons, to mark two ALGs as not being colocalized in a given clade, we first check that the ALGs are detectable in that clade in at least 50% of species and that at least 50% of those species do not have the two ALGs colocalized to the same chromosome.

The final result is a string representation of ALG changes for each species. These results are also saved to the file per_species_ALG_presence_fusions.tsv.

We then summarized inferred changes from per_species_ALG_presence_fusions.tsv with egt perspchrom-df-to-tree, which maps perspective chromosome changes onto the calibrated tree and performs Monte Carlo label–shuffling simulations. The output statsdf.tsv records per-branch fusion and loss-through-dispersal summaries, and the simulation outputs provide the expected background for observed-versus-null comparisons. The reproducible wrapper invokes this stage in pipeline/step6_perspchrom_df_to_tree/run.sh.

One calculated value for each change in the statsdf.tsv file is change_frac_total, which is a measure of how specific this change is to this evolutionary branch. In our previous work (*2*, *3*, *7*), we identified major BCnS ALG–preserving chromosomal changes that happened on specific evolutionary branches, which became fixed before the crown group of that clade, and, consequently, this evolutionary state can be observed in all extant species in that clade. For example, the fusion of BCnS ALGs A1b and A1a occurred in the ancestor of the Bilateria (*2*), and the change_frac_total field in the summary output shows that 98.3% of the inferred A1a-A1b fusion events independently inferred from the viewpoint of individual extant species occurred on the branch leading up to Bilateria. Similarly, the known Ea-Eb fusion is captured in this analysis, and 91.1% of all inferred Ea-Eb fusions occurred on this branch. This field can be used as a cutoff to select the most confidently called ALG change events.

Another value calculated for each ALG change is frac_samples_w_this_taxid_w_this_change, which measures the number of species in this clade in which this change could be detected. For example, despite the fact that 98.3% of A1a-A1b events detected were in the branch leading up to Bilateria (field change_frac_total), this change was only detectable in 54.5% of the species in this clade. This value is reflective of species in which A1a or A1b are no longer detectable because of dispersal.

### Dispersal characterization

We characterized the degree of BCnS ALG dispersal since the myriazoan ancestor as the fraction of BCnS ALG orthologs that remain detectable and significantly grouped on one or more scaffolds. The helper functions formerly described as AnnotateSampleDf.py now live in egt.annotate_sample_df, but the reproducible figure pipeline uses egt decay-pairwise for divergence–time decay curves and optionally egt alg-dispersion for ALG conservation/dispersal plots. The same fields are retained: frac_ologs, frac_ologs_sig, frac_ologs_single, and frac_ologs_{ALG_name}, all based on Bonferroni-corrected one-sided Fisher’s exact tests.

To show the relationship between elapsed evolutionary time and dispersal, we compared genomes in the dataset to the chromosomes of *P. maximus*, which have undergone few interchromosomal changes since the myriazoan ancestor. Pairwise decay curves are now generated by egt decay-pairwise using divergence times emitted by egt newick-to-common-ancestors. Conservation was measured as the fraction of *P. maximus* chromosomal genes significantly conserved on any chromosome in the comparison genome.

### ALG evolution simulations

To estimate whether any category of ALG fusion or loss through dispersal occurred more often than expected under an identical evolutionary scenario, we used the Monte Carlo simulation implemented in egt perspchrom-df-to-tree. The simulation loads observed events from per_species_ALG_presence_fusions.tsv, shuffles the 29 BCnS ALG labels, reinfers event placement on the same calibrated tree, and sums results across simulations to estimate the branch-wise null rate for each event type.

### Correlation inference with species diversity

We measured one-tailed Spearman rank correlations between ALG fusion or loss-through-dispersal rates and species extinction or origination intensity (*31*). The current implementation is egt branch-stats-vs-time, which bins per-branch changes from statsdf.tsv into 1 million–year intervals, computes clade-level rate series with phylogenetic weighting, and emits modified node and edge tables plus per-clade and custom-clade summaries. Tree visualizations are generated by egt branch-stats-tree and egt collapsed-tree.

### Chromosome evolution periodicity analysis

To determine whether changes in chromosomal evolutionary rates have significant underlying periods, we conducted Fourier analyses and Monte Carlo simulations, following the methods described by Rohde and Muller (*31*). Our dataset included rates of ALG fusions and losses through dispersal across vertebrates and protostomes. We truncated the oldest time for each clade to between 250 and 525 million years ago depending on the age of the clade and removed the most recent million-year bin to avoid edge effects in the Fourier transform and polynomial detrending that would distort the frequency analysis. Cubic or quartic polynomials were fitted to the rates, and detrended datasets were created by subtracting these functions. Discrete Fourier transforms were then performed on both raw and zero-padded data to identify the strongest peaks in the spectral power distribution for further analysis.

For each spectrum, we identified the top two or three peaks for Monte Carlo evaluation. Following Rohde and Muller (*31*), we implemented two nulls: an “R” model, a random walk through observed transitions, and a “W” model, a block-shuffle random walk that preserves short-term correlations by partitioning transitions into blocks and shuffling within blocks. In both nulls, the starting rate matched the observed series, which ensures that the end also always matches the observed rates. Unlike prior reports that block number has little effect on background spectra, our chromosome-rate data were sensitive to block size: Varying it changed the fraction of spectra exceeding observed peak magnitudes by roughly 20 to 30%. Because “the choice of 20 blocks is somewhat arbitrary” (*31*) and background variance is high, we ran W-type simulations across 10 to 50 blocks. Each target peak was evaluated with 5000 trials, and we summarized the percentage of trials exceeding the observed magnitude [compare Rohde and Muller (*31*) and fig. S4]. We also computed expectations under an exponential model. For each clade by change type by padding by period combination, this yields a range of support values; medians are reported in the supplementary text section “Long-term patterns in genomic changes,” and the full matrix appears in table S12.

These periodicity analyses are implemented as egt fourier-of-rates. The reproducible wrapper runs this subcommand on the weighted clade-level rate tables emitted by egt branch-stats-vs-time, including the Protostomia-minus-Clitellata and Vertebrata-minus-Teleostei subsets and the Vertebrata-minus-Teleostei maximum-time sweep.

### Simulation of FWM

We sought to simulate the mixing process of chromosomal FWM events on single (simplified) chromosomes. During the simulations, we measured the rate and amount of gene mixing entropy over time on simplified chromosomes. These simulations are implemented in a Python software package called chromsim (https://github.com/conchoecia/chromsim). The software was tested on Python v.3.11.7, and packages used in the implementation include Matplotlib v.3.8.0 (*82*), SciPy v.1.12.0 (*79*), NumPy v.1.26.4 (*83*), and Pandas v.2.1.4 (*71*, *72*).

Such a chromosome consists of genes assigned to two linkage groups A and B. Initially, the two groups are each present in contiguous blocks, only joined to each other at one A-to-B transition. This configuration emulates the state of a chromosome after a recent fusion or translocation event and before any chromosome inversions have spanned the genes that were originally on two separate chromosomes. Next, the chromosome is subjected to a process of stochastic inversions.

In each chromosome inversion cycle, both inversion breakpoints are chosen with a uniform distribution. We only allow inversions between genes; therefore, we can treat the length of the chromosome as a vector of length L, one shorter than the number of genes on the chromosome, L=A+B−1. Therefore, each value between 0 and L represents the cutpoint between a single gene. These two random variables range between [0,L], and the distance between the two random variables, d, has a linear relationship because of the maximum value imposed by L. Given the range of possible values in [0,L], the probability density function for d can be expressed as $f_{D}\left(d\right)=\frac{2\left(L-d\right)}{L^{2}}$ for 0≤d≤L. Therefore, the probability of yielding an inversion of length d linearly decreases.

At the beginning of the simulation and after each inversion cycle, we calculate the mixing value m—a measurement of the entropy of the A and B genes that were previously on separate chromosomes. For a definition of m, see section S13.1.2, page 81, in Schultz *et al.* (*3*). Each simulation is run until the value m reaches a state in which further inversion events do not cause m to change markedly—that is, after a burn-in period when m is exploring the main mass of the probability distribution of m in an ideally mixed genome. After collecting this distribution, it is possible to estimate how many inversions it would take to reach a certain entropy value m observed in reality.

An additional feature of chromsim is that the novelty of local gene neighborhoods are measured after each inversion cycle. Every time there is an inversion, there is the possibility for two or more genes to come into close two-dimensional (2D) proximity to one another for the first time. The simulation keeps track of newly encountered genes in windows of 2 to 10 genes. This feature allows estimation of the number of inversions required to fully explore the space of possible gene combinations after the chromosome fusion event. Chromsim offers the ability to collate the results for many runs and calculate averages or compare the mixing and novel locus encounter behavior under different parameter sets.

We ran chromosome simulations for four different window sizes *W* = 1,2,5,10 by executing the program like so: path/to/main.py -S -a A -b B -w W. A and B are the ALG sizes, the following tuples were used: (A,B) = (4,6), (20,30), (45,55), (100,150), (200,300), (300,400), (450,550). Each combination of chromosome size tuples (A,B) and window sizes *W* was simulated 10 times, unless 2·W>A+B [effectively, this means that (A,B) = (4,6) was not run with *W* = 10]. Mean, SD, median, first and third quartiles, as well as minimum and maximum values were calculated from 11 runs with each parameter set (path/to/main.py -m). One run of each parameter set was plotted to produce trace and dot plot figures (main.py -P -s path/to/output). Box plots of the collected runs were produced with main.py -p -o -f path/to/output.

These simulations can also be run using chromosome sizes and A and B group sizes, inferred from chromosomal orthology information encoded in .rbh files. This mode of operation can infer how far along a chromosome is on the process of mixing through inversions by comparing the observed m to the distribution of m over the course of the simulation. We used *P. maximus* chromosomes that underwent ancient FWM events to demonstrate the varied evolutionary history of these events. First, we identified *P. maximus* FWM events using the odp script in the odp software package commit 8310442. Consistent with previous results (*2*, *3*), we found five chromosomes that are composed of two BCnS ALGs that have fused and mixed. Among the five chromosomes, there are two chromosomes with bilaterian synapomorphies (A1a ⊗ A1b in scaffold NC_047017.1 and Ea ⊗ Eb in scaffold NC_047021.1), two chromosomes with spiralian synapomorphies (J2 ⊗ L in scaffold NC_047018.1 and K ⊗ O2 in NC_047019.1), and one chromosome with a scallop synapomorphy (B2 ⊗ M in scaffold NC_047016.1) (table S14).

### Inversion breakpoint inference

We sought to infer rates at which chromosomal inversions become fixed by reconstructing the evolutionary history of homologous chromosomes in groups of closely related species. To do this, we expanded our previously published software package, breakpointer2 commit 97dda4c (*84*), to automatically infer homologous portions of, or whole, chromosomes before inferring breakpoints. Additional package requirements by breakpointer2 are Matplotlib v.3.8.0 (*82*), NumPy v.1.26.4 (*83*), Pandas v.2.1.4 (*71*, *72*), and SciPy v.1.12.0 (*79*).

Briefly, breakpointer2 works on paf-format whole-genome alignment files generated with Minimap2 v.2.28 (*85*), subsetting the alignments to contain homologous chromosomes that have been identified using odp v.0.3.3 (*3*) or D-GENIES v.1.5.0 (*86*). Only alignments of a minimal length of 100 kb and a quality score of 30 are considered. Collinearity breaks are then identified as discontinuities in the sorted pairwise alignment position table. Alignments are considered contiguous if there is a gap smaller than 5 Mb between them, and they share the same alignment orientation. This gap parameter can be adjusted for different species combinations to minimize falsely identified breakpoints.

To further study whether collinearity breakpoints overlap with insulation boundaries, breakpointer2 searches for relationships between breakpoints and regions between TADs and other 3D chromosomal topological features. The hypothesis that is tested is whether the breakpoints tend to occur between TADs or other 3D topological features. We define regions that occur between TADs or other 3D topological features as regions with low insulation scores. The null hypothesis is that breakpoints do not tend to occur between TADs and 3D topological features, and we model this distribution of randomly sampled insulation scores. To prepare the data for these analyses, the Hi-C reads (*84*) were aligned to reference genomes with chromap v.0.2.3 (*87*) with a quality cutoff of 0. Then, Pairix v.0.3.7 (*88*) was used to bgzip compress the .pairs files from chromap. The program cooler v.0.9.1 (*89*) was used to generate .cool matrices with bin sizes of 5 and 100 kb. The .cool files were then normalized with HiCNormalization from HiCExplorer v.3.7.2 (*90*). The normalized .cool files were balanced using cooler balance (*89*). The normalized and balanced .cool files were converted into the .fance format using FAN-C v.0.9.26 (*91*), and insulation scores were generated with FAN-C for windows of 100 kb, 250 kb, 500 kb, 750 kb, and 1 Mb. The resulting insulation scores, in .bed format, are used as the input for comparing breakpoints with insulation score.

To complete the analysis of breakpoints and insulation scores, first, the actual breakpoint coordinate is computed as the midpoint between the stop and the start of the previous and the next alignment region, respectively. The insulation scores at these locations and for each chromosome are then compared to the random sample across the same chromosome, with the sample size being the same as the number of observed breakpoints. The median insulation score for the random samples are calculated for 100,000 trials to generate a distribution of insulation score medians for a given chromosome. The medians of the observed breakpoint insulation scores are then tested against the random sampling median distribution using a one-tailed permutation test. The one-tailed permutation test yields a false discovery rate of finding an observed median insulation score smaller than the randomized background distribution. That is, a false discovery rate of less than 0.05 represents breakpoints that occur in the lowest 5% of possible insulation scores for an identically sized sample and likely represent regions between TADs or other topological features that have high insulation scores.

We used breakpointer2 to characterize the breakpoints of the octopus species *Octopus vulgaris* (GCA_951406725.2) (*84*), *Octopus sinensis* (GCA_006345805.1) (*92*), *Octopus bimaculoides* (GCA_001194135.2) (*9*), and *Amphioctopus fangsiao* (https://figshare.com/s/fa09f5dadcd966f020f3) (*93*). Using the inferred phylogenetic relationship for these four species (*93*) and by comparing the .bed output of breakpointer2 using BEDTools v.2.31.1 (*94*), we inferred shared and derived inversions along each evolutionary branch for these species. Inversion breakpoints found to exist on two sister species, but not in outgroup species, were assumed to be shared, derived inversions that occurred on the branch leading to the crown group of the two sister species.

### Evolutionary topology methods

The two forms of topological analysis are now implemented in egt phylotreeumap and the Snakemake workflows workflows/phylotree_umap.smk and workflows/phylotree_umap_subsampling.smk. The egt phylotreeumap subcommand group builds per-species distance matrices, constructs sparse all-sample matrices, indexes ortholog-pair columns, runs MGT/MLT UMAP projections, and renders static or interactive plots. Figure-specific UMAP panels are rendered with egt.phylotreeumap_plotdfs.

To create the matrix for MGT matrices, the .rbh files for all species in the analysis are parsed into a compressed distance matrix. The distance that we record in this manuscript is the base pair distance between two loci on the same scaffold. These distances are stored as raw base pair values with no normalization for chromosome length, gene count, or genome size. The selection of distance matrices for the genomes of interest, usually all of the available genomes from a specific clade, is then organized into a SciPy sparse matrix, while the sample information and index of ortholog pairs are stored separately as tab-separated files. Ortholog pairs that are not colocalized on the same chromosome are not recorded in the sparse matrix. To visualize the matrix as a 2D projection, the sparse matrix is loaded from the disk, and the ortholog pairs that are not colocalized on the same chromosome are encoded as 999,999,999,999—a value substantially larger than any known chromosome length in base pairs. This matrix was then embedded into two dimensions with parameter low_memory = True and with a parameter sweep of number of neighbors and minimum distance.

The MLT matrix creation and visualization procedure differs slightly from that of the MGT matrices. Each MLT matrix is a symmetric N×N matrix, where N is the total number of orthologous loci across the entire dataset. The goal of this matrix is to express the topology of a genome for a single genome or collection of species and to be able to compare topologies between species or clades. For this reason, it is necessary to average the distances between ortholog pairs for all of the samples in the comparison.

The MLT matrix phylogenetic weighting is implemented in the adjacency_dag class in egt.phylotreeumap. To perform phylogenetic weighting, we construct a directed acyclic graph reflecting evolutionary relationships among species. Each node’s distance matrix is calculated by normalizing contributions from descendant species according to relatedness, preventing densely sampled clades such as drosophilids or vertebrates from dominating the averaged weights. Ortholog pairs on separate chromosomes are encoded with the same large missing-distance sentinel used for MGT.

A more detailed explanation of the algorithm is below:

1) We construct the directed acyclic graph (DAG) and its inverse graph from NCBI taxid lineage information, and the branch lengths between taxids in the lineages were encoded as 1 s. In this graph, nodes are extant species or ancestors of one or more species. Edges represent branches on the phylogenetic tree, and the value of the edge is the length of the branch. The root is determined and is defined as the node that has no parents. Below, we will refer to a node of the DAG as DAG [node1] and the edge weight between two nodes as DAG [node1] [node2].

2) After constructing the DAG, the branch lengths are normalized, such that the total distance from the root to any leaf is 1. The reason this is done is to enable matrix multiplication to be used to perform the phylogenetic averaging. Therefore, both cladograms and phylograms can be used as input. The algorithm finds the longest path from the root and scales the branch lengths accordingly. Let *P*_longest_ be the longest path from the root to a leaf with ℓ nodes (hence, ℓ−1 edges). We define

remaining_length=1

$$\text{edge}\_\text{length}=\frac{\text{remaining}\_\text{length}}{\ell -1}$$

For each child c of node n

$$DAG\left[c\right]\left[c\right]=\left\{ \begin{array}{cc} x\left(n\right) & \text{if}\;c\;\text{is}\;a\;\text{leaf}\\ x\left(n-1\right) & \text{otherwise} \end{array}\right.$$

The algorithm then recursively adjusts the branch lengths for the subtree of each child node. This is all performed in the function adjacency_dag.normalize_branch_lengths().

3) A distance matrix is generated for each pair of tips (*t_i_, t_j_*), where each tip *t_i_* is one of the species in the tree that is being averaged. The distance between two tips (*t_i_, t_j_*) is the sum of the weights along the path from *t_i_* to the last common ancestor node shared with *t_j_*. The distance matrix, *D*, is then normalized to be between the maximum weight recorded in the matrix.

$$D_{\text{normalized}}=\frac{D}{\text{max}\left(D\right)}$$

4) The phylogenetic weight calculation is as follows:

The total distance for each sample: $T_{i}=\sum_{j}D_{\text{normalized}}\left[i\right]\left[j\right]$

The weight vector for the samples: $W_{i}=\frac{T_{i}}{\sum T_{i}}$

5) The phylogenetic weight matrix is calculated by calculating the dot product of the transposed MGT ${\text{matrix}}^{T}$ and the weight vector w.

$${\text{matrix}}_{\text{phylogenetic average}}={\text{matrix}}^{T}\cdot w$$

6) The final vector is transformed into the N×N distance matrix based on the predetermined mapping of MGT matrix indices to ortholog IDs.

We generated a standalone interactive Bokeh Hypertext Markup Language (HTML) file with egt phylotreeumap plot-html from the precomputed MGT UMAP table (n_neighbors = 250, min_dist = 1.0), plotting each genome by its UMAP coordinates and manuscript taxonomic color. The visualization includes hover labels, taxid/taxonomy search, lasso selection, table export, and a taxid-linked calibrated Newick tree and can be opened locally without a server.

### Graph database integration

We built a Neo4j graph database (Community Edition v.5.26.13) of orthology relationships across the species in this study, using the .rbh files from identifying BCnS ALGs as input (fig. S6, A and B). The graph contains four node types—ALGs, orthogroups, genes, and chromosomes—linked by orthologous (chromosome-ALG, gene-orthogroup) and location (gene-chromosome, orthogroup-ALG) edges. Nodes carry NCBI taxid, assembly, species, lineage, and name properties, with taxid-prefixed labels (e.g., CHR9606 and GENE9606) for taxonomically scoped queries. Chromosome-ALG edges were assigned when the Bonferroni-corrected Fisher’s exact *P* value was <0.01; gene-orthogroup edges followed reciprocal best-BLAST-hit relationships. The graph was visualized in Neo4j Browser v.2025.8.0, and we provide a Cypher-based query tool for retrieving subgraphs by species and ALG (fig. S6, A and B). The database is distributed as a Neo4j image, GraphML, JSON, and CSV.

### GO enrichment of stable pairs

To test whether stable ALG family pairs are enriched for shared GO annotations, BCnSSimakov2022 ALG family identifiers (*2*) passing the per-clade stability filter were mapped to NCBI human gene IDs via the family-naming-map postanalysis (egt family-naming-map subcommand) and to GO terms via the NCBI human gene2go table (both at https://ftp.ncbi.nlm.nih.gov/gene/DATA/) (*95*) augmented by GO Consortium annotation (*96*). For each clade, hypergeometric tests for GO term enrichment were computed against the full BCnS ALG (*2*) family universe using both egt go, with Benjamini-Hochberg false discovery rate correction applied across the entire parameter sweep (*97*). All results generated with egt go were compared against GOATOOLS (*98*) for validation.

### Stable pair selection and visualization

To present examples spanning the full distance distribution rather than a single mode, candidates within Vertebrata and Arthropoda were binned by the empirical cumulative distribution function (CDF) of mean_in, and one example was selected per target percentile (5th, 15th, 25th, 35th, 45th, 55th, 65th, 75th, 85th, and 95th). Within each percentile window, one pair was chosen. For each selected gene pair, lanes were drawn for each species with both anchor genes resolved on the same scaffold, in the order produced by a descending phylogenetic sort of the calibrated BCnS_2026 species tree from earlier steps of the manuscript. Phylogenetic rate conservation tracks were obtained from the UCSC genome-browser (*99*): *Homo sapiens*: phyloP 100-way (*100*); *D. melanogaster*: phyloP 124-way (*100*) and phastCons 124-way (*101*) (phyloP124way/dm6.phyloP124way.bw and phastCons124way/dm6.phastCons124way.bw). For *H. sapiens*, the layered H3K27Ac track (wgEncodeRegMarkH3k27ac), representing ChIP-seq enrichment across seven cell lines, was also used (*102*).

### TF binding tracks and per-pair enrichment

To assess for TF enrichment in the observed gene pairs, we identified nearby TFs in the pairs and compared them against all TFs present in the genome in the same window near other genes. Aggregated TF ChIP-seq peaks were downloaded from ReMap2022 (*103*). A focal species promoter universe was constructed once per build by collapsing isoforms in the focal species .chrom.gz and defining the promoter window of each gene locus as the 2-kb region 5′ of the transcription start sites. For each TF *t* in ReMap2022, we calculated the per-TF prior probability *p_t_* = *K_t_*/*N*, where *K_t_* is the count of promoters with at least one *t* peak intersecting their 2-kb upstream window and *N* is the count of promoter windows in that genome. In figure panels, the top *N* TFs by specificity rank are drawn as thin peak-tick lanes above the focal species gene track; ticks falling inside either anchor’s 2-kb upstream window are highlighted in the TF’s color to indicate promoter-proximal binding. ReMap pools ChIP experiments across many cell types and laboratories, so cobinding of a TF at both promoters does not imply binding in any specific biological context.

## Supplementary Materials

### The PDF file includes:

Supplementary Text Figs. S1 to S14 Legends for tables S1 to S15, and S17 Table S16 Legend for supplementary file 1 Supplementary Files Dryad Repository Content Guide References
