# Supplementary material for: Topological mixing and irreversibility in animal chromosome evolution
Source: Sci Adv. 2026 Aug 19;12(34):eadz5561. doi: 10.1126/sciadv.adz5561 (PMC13508932; doi:10.1126/sciadv.adz5561)
Supplement: Supplementary file 1 [file sciadv.adz5561_sm.pdf]

Science Advances
AAAS

Supplementary Materials for

Topological mixing and irreversibility in animal chromosome evolution

Darrin T. Schultz *et al.*

Corresponding author: Darrin T. Schultz, dts@lehigh.edu,
Oleg Simakov, oleg.simakov@univie.ac.at

Sci. Adv. **12**, eadz5561 (2026)
DOI: 10.1126/sciadv.adz5561

The PDF file includes:

Supplementary Text
Figs. S1 to S14
Legends for tables S1 to S15, and S17
Table S16
Legend for supplementary file 1
Supplementary Files Dryad Repository Content Guide
References

Other Supplementary Material for this manuscript includes the following:

Tables S1 to S15, and S17
Supplementary file 1

Supplementary Text - Glossary

To clarify the intended meanings of our text, we include below a glossary of terms defined in this manuscript, or existing terms and the meaning of these terms based on previous publications.

New terms introduced in this manuscript include:

1. Entanglement
2. Evolutionary genome topology
3. Multi-genome topology
4. Multi-locus topology

Otherwise, all terms present in this glossary have been published in peer-reviewed articles, and we cite the original literature when possible.

**Algebraic changes**	The *four* elementary operations or relationships that underlie metazoan chromosome dynamics. (A) **Syntenic equivalence** means chromosome conservation across species, and the types of change operations are (B) **Robertsonian translocation**, (C) **Centric insertion**, (D) and an extension of the above two processes, **Fusion-with-mixing**. These *three* algebraic changes are composed only of **building** new chromosomes from ancestrally conserved whole chromosomes or chromosomal arms (such as BCnS ALGs), and do not account for chromosomes splitting, fissions, or interchromosomal translocations (some may call such changes "non-algebraic"). See **genome tectonics**.<br>- Term introduced in Simakov et al (*2*).<br>- This term is also used in (*13*).<br>- This term is not used in (*9*).
**Ancestral linkage group**	(ALGs) Refers to groups of genes that are conserved together over macroevolutionary time, usually together on the same chromosome. The term ALG does not require that the loci are one contiguous block. Therefore, ALGs can be an extension of the concept of **Macrosynteny**. In Putnam et al (*5*), the authors refer mostly to chordate linkage groups, then use the term 'ancestral linkage group' once.<br>- Term introduced in (*5*).<br>- This term is used in (*2*, *3*, *7*, *9*, *104*, *105*).<br>- This term is not used in (*17*).
**Centric insertion**	One chromosome is inserted into another, denoted symbolically by ↘. This is an irreversible change.<br>- Symbol denoting this process (↘) introduced in Simakov et al (*2*).

Chromosomal element — In our usage, similar to **Ancestral linkage group**. Refers to groups of genes or loci that are conserved together as units over time. In the original usage by Sturtevant and Novitski (*6*), it refers to contiguous homologous regions that still persist in closely related species, *Drosophila* in their case. Earlier, McClintock (*106*) used this term, but it only referred to parts of the genome that shape the nucleolus organization.
- Term introduced in (*6*).
- Term used in (*2*, *7*).
- Term not used in (*3*, *5*, *9*, *104*, *105*, *107*).

Chromothripsis — A single-generation catastrophic shattering and random reassembly of a chromosome, first described after the observation of the phenomenon in tumor cells.
- Term introduced in Stephens et al (*41*).

Dispers(al/ed) — **Dispersal:** The process of loci being spread out across other genomes through small, difficult-to-detect translocations, transposable element-mediated transposition, or other mechanisms. **Dispersed**: Genes that have undergone dispersal.

Previous Usage:
- Earlier use in (*108*) to talk about homeobox gene clusters splitting apart from an ancestral clustered state to spread across a single chromosome. This meaning is akin to what we call mixing, which is an intrachromosomal process. The use of dispersal in Luke et al (*108*) as an intrachromosomal process differs from our meaning (*2*, *7*), in which dispersal is an interchromosomal process.
- *Dispersed* introduced in (*7*).
- *Dispersal* introduced in Simakov et al (*2*).
- Term not in (*9*, *17*).

Entanglement — When two or more loci come under the same regulatory or topological constraint, and the mixing of their regulatory interactions (such as enhancer-promoter links) results in a locus in which reverting the locus back to the ancestral pre-entangled state by translocation or other means, is selected against.
- ***Term introduced in this manuscript.***

Fusion-with-mixing The genes of two chromosomes are brought together (by either Robertsonian or end-to-end translocation or centric insertion) and, through a series of intrachromosomal rearrangements, become interspersed, denoted symbolically by the outer product symbol: $\otimes$. This is an irreversible change. Can be used to determine phylogenetics, in the case where shared, derived fusion-with-mixing events are found.
- Term introduced in Simakov et al (*2*).
- Symbol denoting this process ($\otimes$) introduced in Simakov et al (*2*).
- Term also used in (*3*), or in a sense in (*9*, *17*).

Genome/chromosome tectonics The basic mechanisms and the systematic construction of contemporary chromosomes from ancestral units. See **algebraic changes**.
- Term introduced in Simakov et al (*2*).
- Term not used in (*9*, *17*).

Linked Linked refers to loci that are not only **mixed**, but also are conserved together over evolutionary time.

Mixed Mixed refers to the state of loci that were previously on separate chromosomes, but are now both on the same chromosome and the loci have become intermingled through inversions. For example, two separate chromosomes, [AAAAA] and [BBBBB] fused into a single chromosome [AAAAABBBBB]. Then, after a series of inversions the A and B loci are in rearranged, stochastic state [BAABABBAAB]. It is not necessary that A and B have the same number of loci being considered. It is also not necessary that the A and B loci are alternating in a one-after-the-other pattern, like [ABABAB…].

Modality See *mode*. In this manuscript we use the term *mode* where others may have used *modality*.

Mode See also *modality*. We define a "mode of genome evolution", or "mode of chromosome evolution" as how chromosomes change over time. The mode of evolution is defined by the sum of the effects of translocations, inversions, and chromosome fissions.

The development of this term to the meaning above comes from a long line of research dating back to early comparative genomics studies predating modern DNA sequencing. One of the first usages of this term based on our searches was in reference to whether DNA evolution is 'ignorant' or 'selfish' (1980) (*109–111*). Later, "Mode and tempo in primate chromosome evolution" was used to discuss the divergence in karyotype between primate species (1983) (*112*). The term "Modes of Chromosomal Evolution" was used to reference the discovery of paracentric inversions in potato chromosomes

(1988) (*113*), and later for karyotype evolution in carcinomas (2003) (*114*). The first usage in the context of "mode of genome evolution" or "mode of chromosome evolution" was in reference to macrosynteny shared between ascomycete fungi species, which they called "mesosynteny" (2011) (*32*). The term "patterns of metazoan evolution" was used in 2017 (*33*), and later the term "mode of chromosome change" was used in the sense of our definition above, in reference to chromosome FWM events.

Multi-genome topology Acronym: MGT. A topological comparison of entire genomes. Each sample (dot on a plot) is a single genome. Each sample has a vector of comparison that contains all of the possible pairwise interactions for an orthology set. The values of the pairwise interactions could be base pair distance, base pair distance divided by chromosome length, Euclidean distance in 3D space, or other weight values derived from these or other sources. 2D projections of this matrix are called MGT plots.
- ***Term introduced in this manuscript.***

Multi-locus topology Acronym: MLT. A topological comparison of the loci in a single genome or a phylogenetically weighted representation from a clade. Each sample (dot on a plot) is a single locus. In this manuscript, we use a 2361 x 2361 matrix of the BCnS ALG orthologs as the loci. Each sample, a single BCnS ALG ortholog, has a vector that contains the distance of this BCnS ALG to all other orthologs. This matrix is symmetric. The values of the pairwise interactions could be base pair distance, base pair distance divided by chromosome length, Euclidean distance in 3D space, or other weight values derived from these or other sources. 2D projections of this matrix are called MLT plots.
- ***Term introduced in this manuscript.***

Myriazoa A clade containing all extant animals except for the comb jellies (Ctenophora). In this phylogenetic hypothesis, Ctenophora is the sister clade to Myriazoa. The clade Myriazoa is not compatible with the phylogenetic hypothesis of Eumetazoa, in which Porifera (sponges) is the sister clade of all other animals. Myriazoa is synonymous with the term Benthozoa.
- Term introduced in Schultz et al (*3*).
- Benthozoa introduced in Erives & Fritzsch (*115*).

Robertsonian translocation Two chromosomes fuse without mixing, with a stable boundary denoted symbolically by • or ●. Robertsonian fusions are reversible if no mixing has occurred. May also describe end-to-end fusions (*2*).
- Symbol denoting this process (●) introduced in Simakov et al (*2*).

Saltation The process or result of **saltatory changes** occurring.

Saltatory changes Dramatic changes of chromosomes, and consequently of a whole genome, from the ancestral state. Usually in this process there are not only **algebraic changes**, as a complete fusion of chromosomes would result in a very small karyotype, but also chromosomal fissions.

Synteny The conserved colocalization of genes on the same "ribbon" (chromosome) across species. The term synteny in classical genetics simply meant genes located on the same chromosome. Its modern usage in comparative genomics (conserved gene linkages across species) often now refers to the preservation of gene order over large chromosome spans when comparing multiple genomes (what geneticists call **collinearity**). The term **macrosynteny** refers to the conservation of chromosome identity without regard to the gene order, while the term **microsynteny** refers to the conservation of the gene identities in local neighborhoods. Unlike collinearity, which requires conserved gene order, clusters of genes can be microsyntenic even if the order is shuffled, some genes are absent, and some new genes have become interleaved in the cluster.
- Concepts introduced in early work like Dobzhansky & Sturtevant (*116*), Sturtevant & Tan (*117*), Muller (*118*), and more. The term was refined over many decades and publications, including Renwick (*119*), Passarge et al (*120*), and many more.

Syntenic equivalence One of the **algebraic changes** and part of **genome tectonics**. This is conserved synteny without regard to collinearity, denoted symbolically by ≡.
- The earliest manuscript with this term we could recover was in reference to the homology of the human and mouse X chromosome by Schlessinger et al (*121*).
- Term introduced in the context of all chromosomes in Simakov et al (*2*).
- Symbol denoting this process (≡) introduced in Simakov et al (*2*).

Whole-genome duplication A whole genome duplication is the process by which all chromosomes in an organism's genome are duplicated, resulting in a polyploid individual. The adjective indicating that two chromosomes are siblings after a duplication is *homeologous*. Over time, these chromosomes extensively lose genes and eventually contain a subset of the original chromosome.
- The concept of WGD as a driver of vertebrate genome evolution originates from Susumu Ohno (*37*).

- The term WGD and 2R hypothesis were later supported with genomic data by Dehal & Boore (*38*).

Supplementary Text - Long-term patterns in genomic changes

There is also evidence for periodicity in species origination and extinction (*31*, *122*), most notably in cycles of 62- and 140 million years (*31*, *122*). The first hypotheses invoked possible solar, geological, and biological explanations to these cycles (*31*), while subsequent work pointed out that troughs in these cycles coincide with periods of low sea level as a biodiversity-reducing macroevolutionary force (*123*, *124*). We therefore asked whether chromosomal change rates exhibit comparable periodic structure, detectable from genome evolutionary histories rather than fossils, and applied Fourier analyses with Monte Carlo simulations to vertebrate and protostome chromosomal rate change series, following Rohde and Muller (*31*). Briefly, the analysis detrends the rates of chromosomal change (**fig. S4A)**, Fourier transforms the residuals into frequency space (**fig. S4B**), checks for significance of different periods of change (**fig. S4C,D**), and fits the supported periods to the detrended rates of chromosomal change (**fig. S4E,F**). For significance testing, we performed two types of random walks through the observed data (**Methods, fig. S4A-F**). We focused our analyses on large clades with hundreds to thousands of species, and excluded subclades with massive genome rearrangements. For example, we analyzed Vertebrata (*n* = 2,007 genomes) minus Teleostei (*n* = 853 genomes) given the teleost whole-genome duplication (*125*), and Protostomia (*n* = 3,394 genomes) minus Clitellata (*n* = 21 genomes) because of their rearrangements (*13*–*15*).

In vertebrates minus teleosts, the periods with strongest support after significance testing were 70 and 87.2 million-year periods for BCnS ALG fusions (97.6% and 96.0% support, Monte Carlo simulations, *n* = 5000) (**fig. S4A-F**, **table S12**). Looking in well-represented subclades within Vertebrata, we found a similar period of 88.7 million years within Tetrapoda (96.3% support), and within Amniota periods of 76.3 and 113.2 million years (99.1% and 92.4% support). In protostomes minus clitellates, the top supported period is 71.3 million years for ALG dispersals (89.9% support), and within Neoptera (*n* = 2,662 genomes) the top supported periods were 100 and 125 million years for ALG dispersals (99.3% and 93.6% support). These results are similar, but not identical to, the cadences reported for Phanerozoic origination and extinction (*31*, *122*) (**fig. S4**, **table S12**). Clade-specific changes, such as the periods of 113.2 million years in Amniota and 125 million years in Neoptera could be the result of a shared external pacing of genome restructuring that is modulated by lineage-specific mechanisms, like bottleneck-driven fixation in protostomes and rediploidization dynamics in vertebrates.

The correlation analysis and spectral results together indicate that protostome and vertebrate diversification were associated with distinct chromosomal dynamics that varied in intensity over time. It is unclear whether weak correlations between chromosomal changes and species origination or extinction are bona fide or because of inference biases in tree construction, the long-discussed fossil sampling biases (*124*), or methodological issues. Notable challenges are that event times are not identifiable at a finer resolution than the branches on which they are inferred, and that early timepoints have sparse branch representation, both making more recent chromosomal changes easier to measure. However, as chromosome-scale genome sampling improves along with a pan-animal dated phylogenetic tree, these analyses provide a baseline measurement and methodological basis to better understand the chromosomal changes underlying animal evolutionary history. Future analyses may focus on the interface of shifting

geologic (*123*) and selective constraints, such as population bottlenecks following near-extinction events.

The observed periodicity, whether caused by internal genomic factors or external factors (e.g., geological changes), generally suggests multiple periods of chromosome-scale remodeling opportunities, providing for such potential at least several times in each major animal lineage. We note, and others have noted (*31*), that the number of 62 million years is a consequence of the sampling rate and number of observations, therefore the mechanistic reasons for such periodicity remain elusive. Nevertheless, it is important to highlight that genomic data alone reflects macroevolutionary periodicity and arrives at a similar period duration estimate as analyses of fossil deposits, warranting further investigations into geologic phenomena, like sea-level lowstands (*123*), as macroevolutionary forces in speciation and extinction. Future work in this area might focus on a refinement of node ages across the tree of life, and finer modeling of uncertainty of node ages, to better ascertain the variances in the periodicity of genomic changes over time.

Supplementary Text - Gene Ontology of clade-specific close or stable locus pairs

To test whether the clade-specific stable or close locus pairs are enriched in function, process, or cell component, we performed Gene Ontology (GO) term analyses in 28 metazoan clades spanning animal diversity. Analyzing stable and close locus pairs as total gene groups, clade-specific stable and close locus pairs are overrepresented for GO biological processes (BP), molecular function (MF), and cellular component (CC) terms that, in some cases, track with the distinctive biology of each clade. Below we summarize the strongest enrichments for select well-sampled clades (false discovery rate (FDR) $q \leq 0.01$, hypergeometric enrichment with Benjamini and Hochberg correction) for clades with adequate sampling (*n* genomes ≥ 40). Our calculated enrichment values were consistent between our custom implementation (egt go) and an established GO analysis pipeline, GOATOOLS v1.6.4 (*98*) (Pearson $r = 0.997$, GO terms between tools identical in 20 of 28 clades) (Materials and Methods).

Nematode-specific locus pairs ($n = 139$ genomes) yielded the smallest *q*-values among all clades tested. They were enriched for nucleotide binding (GO:0000166; 8/48 where k/n, fold = 12.3, $q = 1.4 \times 10^{-5}$), minus-end-directed microtubule motor activity (GO:0008569; 3/19, fold = 90.4, $q = 1.2 \times 10^{-4}$), and the dynein complex (GO:0030286; 3/19, fold = 72.3, $q = 1.5 \times 10^{-4}$). These three terms jointly implicate dynein-dependent microtubule transport used in the ciliated sensory neurons that are a defining feature of nematode chemosensation (*126*, *127*).

Deuterostome-specific locus pairs ($n = 2{,}202$ species) were dominated by the cap-dependent translation initiation apparatus: translation initiation factor activity (GO:0003743; 10/154, fold = 5.95, $q = 7.5 \times 10^{-4}$), eukaryotic 48S preinitiation complex (GO:0033290; 6/154, fold = 8.92, $q = 1.8 \times 10^{-3}$), and eukaryotic translation initiation factor 3 complex (GO:0005852; 6/154, fold = 8.92, $q = 1.8 \times 10^{-3}$). At the BCnS Ancestral-Linkage-Group level, the ten contributing families of GO:0003743 partition 5/10 onto ALG J2 (EIF2S1, EIF3E, EIF3H, EIF3I, and COPS5), with EIF3G on M, EIF3M on O1, EIF2B4 on J1, MCTS1 on F, and EIF4E on Qc.

One related association is the colocalization of the genes EIF2S1 (Simakov2022BCnS_genefamily_3869) and ATP6V1D (Simakov2022BCnS_genefamily_1312)

on the branch leading to vertebrates. These loci are on separate chromosomes outside of deuterostomes, and also on separate chromosomes in ambulacrarians (i.e., *Lytechinus* spp., *Ptychodera flava*, etc.) and in non-vertebrate chordates (i.e., *Branchiostoma* spp., *Ciona intestinalis*, etc.) (**table S17**). However, within vertebrates (n = 2,001 species) this was the most tightly colocalized pair in the eIF-adjacent set, with a mean in-clade scaffold distance of 50 kb versus a mean out-of-clade distance of approximately 29 Mb. While EIF2S1 is part of ALG J2, and ATP6V1D is part of ALG A1a, this pair appears to be the result of a single-gene or small translocation of A1a to a J2-containing chromosome. EIF2S1 encodes eIF2α, whose Ser51 phosphorylation is part of the molecular response to endoplasmic reticulum stress (*128*). ATP6V1D encodes the V1-D subunit of the vacuolar V-ATPase proton pump which acidifies intracellular compartments (*129*). In human, these two genes are coregulated at the transcriptional level by nuclear respiratory factor 1 (NRF1) (*130*), and sit with their 5' transcription start sites on opposite strands in the head-to-head orientation within 500 bp of each other, sharing the same H3K27Ac-rich transcription factor binding region (**fig. S12**). Whether the subchromosomal proximity is itself under regulation-driven negative selection (through shared enhancer architecture, TAD-level coregulation, or a chromatin-loop-level integrated stress response (ISR) switch) remains to be tested, however, the pair is a promising candidate for a cis-regulatory follow-up given that 90.4% (n = 1,910 of 2,113) of vertebrate species' genomes contain this stable and close pair.

Chordate-specific locus pairs (n = 2,147 species) echoed the Deuterostomia translation-machinery signal with translation initiation factor activity (GO:0003743; 7/79, fold = 8.12, $q = 2.2 \times 10^{-3}$), and added two closely related ribosome-maturation hits: rRNA processing (GO:0006364; 5/38, fold = 10.8, $q = 5.0 \times 10^{-3}$) and RNA processing (GO:0006396; 5/38, fold = 9.13, $q = 5.7 \times 10^{-3}$). Vertebrate gene pairs (n = 2,001 species) were enriched for endocytic recycling (GO:0032456; 8/144, fold = 5.8, $q = 1.2 \times 10^{-2}$), chromatin binding (GO:0003682; 5/20, fold = 5.3, $q = 1.9 \times 10^{-2}$), and rRNA base methylation (GO:0070475; 3/72, fold = 31.8, $q = 5.2 \times 10^{-3}$). The $k = 8$ endocytic-recycling term is the strongest vertebrate-specific hit. We also found two $k = 2$ neural-patterning hits, one for adult locomotory behavior (GO:0008344; 2/18, fold = 42.4, $q = 1.2 \times 10^{-2}$) and another for negative regulation of neuron projection development (GO:0010977; 2/18, fold = 31.8, $q = 1.2 \times 10^{-2}$).

Bony-fish-specific entanglements (Teleostei, n = 839 species) yielded a significant methyltransferase trio: S-adenosylmethionine-dependent methyltransferase activity (GO:0008757; 3/20, fold = 68.7, $q = 2.4 \times 10^{-4}$), RNA methyltransferase activity (GO:0008173; 3/20, fold = 57.3, $q = 2.4 \times 10^{-4}$), and rRNA base methylation (GO:0070475; 3/41, fold = 55.9, $q = 4.4 \times 10^{-4}$). The three gene ontology terms are partially overlapping (same SAM-dependent methyltransferase orthologs populating the BP, MF, and CC slices).

All 28 clades considered had at least one gene ontology term enriched. The locus pairs can be found in **table S17**.

Supplementary Text - State formulation of the (sub)chromosomal mixing process

Probability vector and matrix multiplication of genomic states: This study suggests the presence of distinct irreversible evolutionary trajectories in animal genomes, with different degrees of irreversibility, depending on the number of components that are being mixed and the strength of their coupling (for example, chromosomal meiotic constraints or regulatory linkages at the subchromosomal level). The irreversibility is a product of mixing of genes that are located on distinct evolutionary units. In our previous studies (*2*, *7*), these units were simply the chromosomal elements (or chromosomes) themselves. In this study, we generalize this mixing to include mixing of previously separated subchromosomal elements.

This degree of irreversibility depends on the size of the mixing units (chromosomal versus subchromosomal) and mechanistic reasons (regulatory linkage, meiotic constraints) behind the fusion process. An important property of the mixing is the loss of the information that the constituent elements can be separated back into the more ancestral and distinct units. For this reason, we can only identify the existence of these ancestral states through evolutionary outgroup comparisons. To begin to study these processes within a more rigorous and quantitative framework, we describe a mathematical approach to quantify the degree of irreversibility and its application in the manifold approximation context presented in this paper.

(Sub-)chromosomal fusion-with-mixing representation: We treat each chromosome as a coordinate system assigning a probability of observation for a given genomic feature. For example, the probability of finding a gene within a chromosome can be defined as a probability density vector of a size of total chromosomal positions. To find two genes to be colocalized at the same coordinate is simply the inner product of the two corresponding gene vectors (**a * b , or < a | b >**). Genomic features move according to their propagation mechanism, such as translocations. An 'evolution operator' U multiplied by a given state vector (**U | a >**) gives the new probability of observing feature **a** at the next evolutionary step. The system of two elements a and b can be described as a tensor product of these two, $\mathbf{a} \otimes \mathbf{b}$, as that (two-dimensional) matrix will peak at a point where both densities are highest in relation to the coordinate systems of a and b. For systems of many genes, this tensor product is simply generalized in a longer string of products ($\mathbf{a}\otimes\mathbf{b}\otimes\mathbf{c}\otimes\mathbf{d}$ ...).

In our previous studies, we represented the fusion-with-mixing process as a tensor product of two chromosomes. However, this is not a completely accurate representation, as the tensor (outer) product simply defines a combination of two coordinate systems and correspondences of each element between the two coordinate systems. If a gene '**a**' on chromosome **A** has probability density of **a=(1,0,0)** and gene '**b**' on chromosome **B** has probability of **b=(0,0,1,0)**, then $\mathbf{a} \otimes \mathbf{b}$ **= (0,0,1,0 ; 0,0,0,0 ; 0;0;0;0)** (or an expanded vector). This describes the system state according to the corresponding coordinate systems (e.g., chromosomal position) of **a** and **b**, but does not provide a framework to qualitatively or quantitatively describe how and how well chromosomes **a** and **b** have become mixed since their initial fusion event.

To represent the mixing property one has to measure the 'entanglement' of the two vector spaces, where, as an initial proxy, correlative measures (such as Pearson correlation) can be used. An initial, unmixed, state consisting of two fused chromosomes (via a Robertsonian fusion), each of size two, and each containing a single gene that can be found with equal probability at either

of the two positions, can be represented as **a = (0.5,0.5,0,0)** and **b = (0,0,0.5,0.5)** and **a ⊗ b = (0, 0, 0.25, 0.25 ; 0, 0, 0.25, 0.25 ; 0, 0, 0, 0 ; 0, 0, 0, 0)**. Similar to the eigenvector decomposition, one can then use singular value decomposition of **a ⊗ b** to find that this matrix's first singular value is explaining 100% of the matrix, suggesting that **a** and **b** can be decomposed and are therefore not yet mixed. However, if the two genes translocate within the newly formed fused chromosome, one possible resulting matrix is **(0, 0, 0, 0; 0.25, 0, 0.25, 0; 0.25, 0, 0, 0; 0.25, 0, 0, 0)**, and the 1st singular value will explain less than 100% of variance in the matrix indicating that a and b coordinates cannot be decomposed clearly any longer, reflecting mixing of the two ancestral states. Together, this suggests that fusion (without yet mixing) is represented by the tensor (or outer) product (⊗ or , **|a><b|**), whereas the mixing itself is moving the elements within this state matrix. Therefore, braiding (×, as defined in category theory) is mathematically a more appropriate representation of the mixing process. In our current implementation in this manuscript, we are using distance between orthologous genes as input for dimensionality reduction approaches and manifold projection. This allows us to compare evolutionarily very distant genomes, and focus on the variation in the genomic distance for the profiled set of orthogroups (n = 2,361). In this context, the positional information of the example above is encoded by the orthogroup pair identifier. In the future, instead of profiling orthogroups, this approach will also allow generalization to other genomic features, yet always requiring a high-confidence underlying homology analysis.

In a multi-dimensional system represented by the tensor product of its components' state vectors, a single mixing event (analogous to a chromosomal inversion or translocation) of the components along their coordinate systems is achieved by swapping values within the matrix. Each swap alone, without subsequent changes, is reversible (single braid). Irreversibility comes in when the topology-driven mixing results in a state where a and b identities cannot be distinguished anymore, without the knowledge of their separate identities from the outgroups. Thus, correlative approaches such as singular value decomposition, principal component analysis, and manifold approximation/projection are useful for the empirical identification of mixed states in genomic spaces. Additionally, singular value decomposition (or any other topological entropy measurements) can provide important insight into the 'strength' of mixing, with stronger mixing of the individual states being less likely to reverse.

Supplementary Figures and Captions

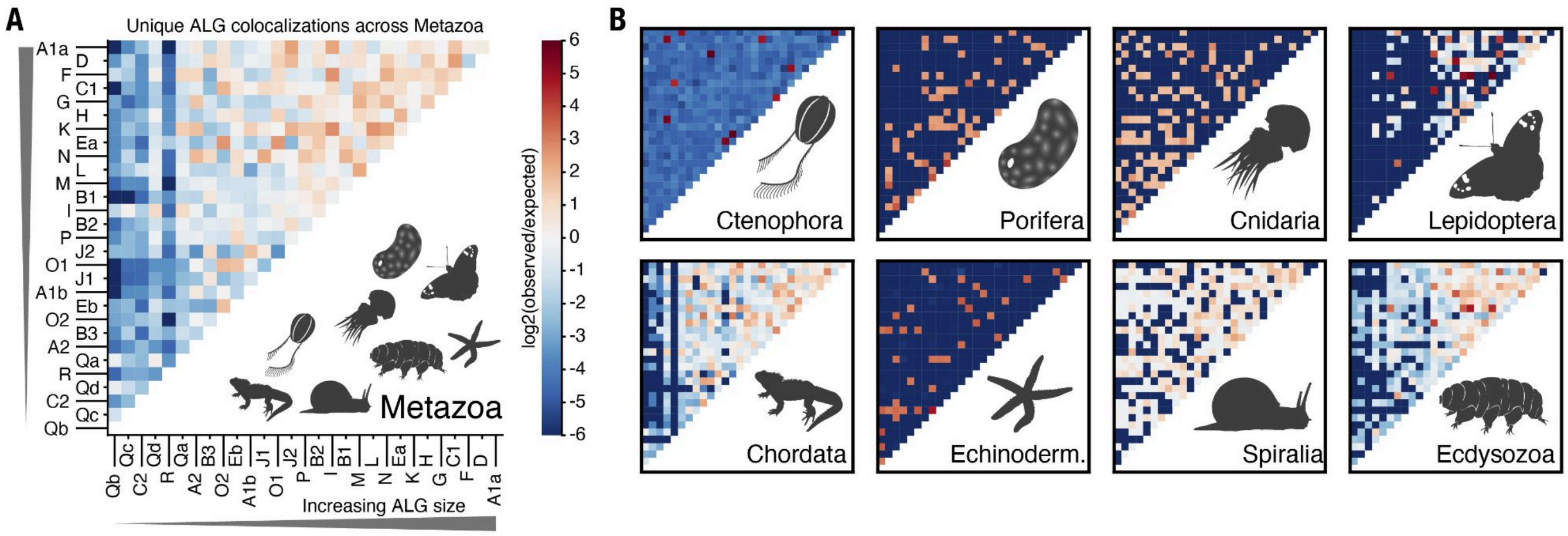

Fig. S1. Phylogeny-aware counts of BCnS ancestral-linkage-group (ALG) colocalizations. (**A**) Metazoan overview (*n* = 5,821 genomes). Heatmap shows, for every pair of BCnS ALGs, how often their loci are found on the same chromosome within a species, expressed as $\log_2$(observed/expected). The expected value is calculated from a randomized, phylogenetically aware null model in which BCnS ALG labels are shuffled, then the lineage-specific ALG fusions and losses through dispersal are recorded along an identical phylogenetic tree. Red indicates enrichment (pairs colocalize more often than expected because of random chance), blue indicates depletion (pairs colocalize less often than occurs because of random chance), white is near expectation. Only the upper triangle is plotted because the matrix is symmetric. Rows and columns are BCnS ALGs (labels along the axes) ordered by increasing ALG size from left to right and bottom to top; the gray bar indicates relatively increasing ALG sizes. Key pattern: across Metazoa, smaller ALGs are generally depleted for colocalization, whereas larger ALGs tend to be neutral or enriched, consistent with greater retention and detectability of larger linkage groups. (**B**) Clade-specific heatmaps using the same scale and ordering as in **A** for the indicated groups. These panels illustrate that BCnS ALG colocalization, and thus inferred fusion propensity, varies among clades as a joint function of their chromosome numbers and lineage-specific dispersal and rearrangement dynamics (see also **Fig. 3**). Over- or under-representation of ALG combinations may be explained by random convergent fusions, but could also be a result of long-term impact of specific fusions to generate selectively beneficial or disadvantageous configurations.

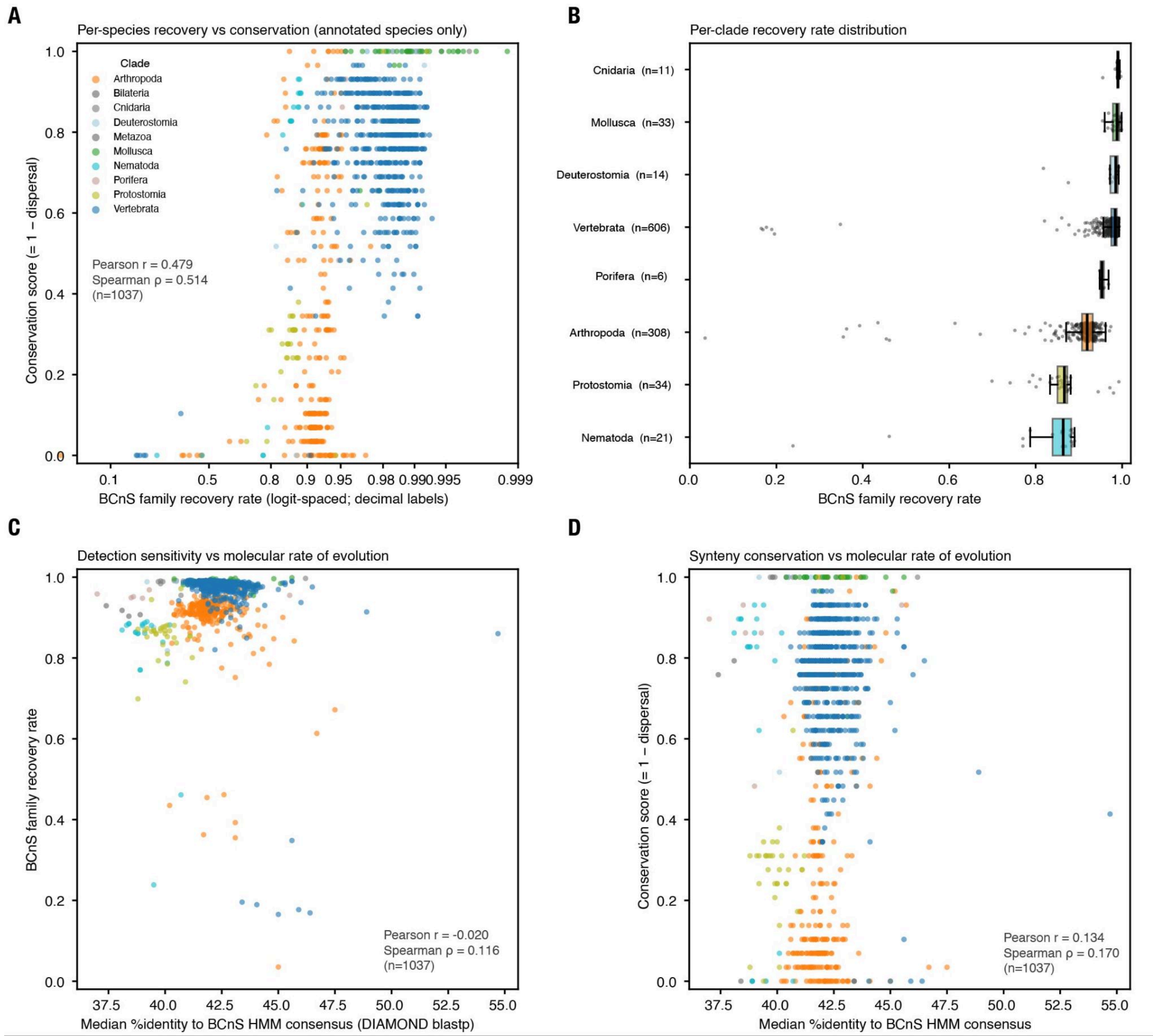

Fig. S2. **Homology detection failure audit**. All panels are per-species, restricted to the 1,037 species represented by 1,088 genomes with a publicly available annotation. (**A**) BCnS gene-family recovery rate versus synteny conservation score, colored by clade. Whole-cohort Pearson $r = 0.479$, Spearman $\rho = 0.514$ ($n = 1{,}088$); $\rho > r$ modestly, consistent with a real but nonlinear monotonic association concentrated in a few clades. (**B**) Per-clade box-and-strip plot of recovery rate, ordered by median. (**C**) Median per-species % identity to BCnS hidden Markov model (HMM) consensus sequences (DIAMOND blastp) versus recovery rate. The relationship is essentially flat (Pearson $r = -0.02$, Spearman $\rho = 0.12$), consistent with RBH detection being a near-binary call rather than a graded function of sequence identity once a threshold is met. (**D**) Median % identity versus conservation score; weak whole-cohort association ($r = 0.13$, $\rho = 0.17$).

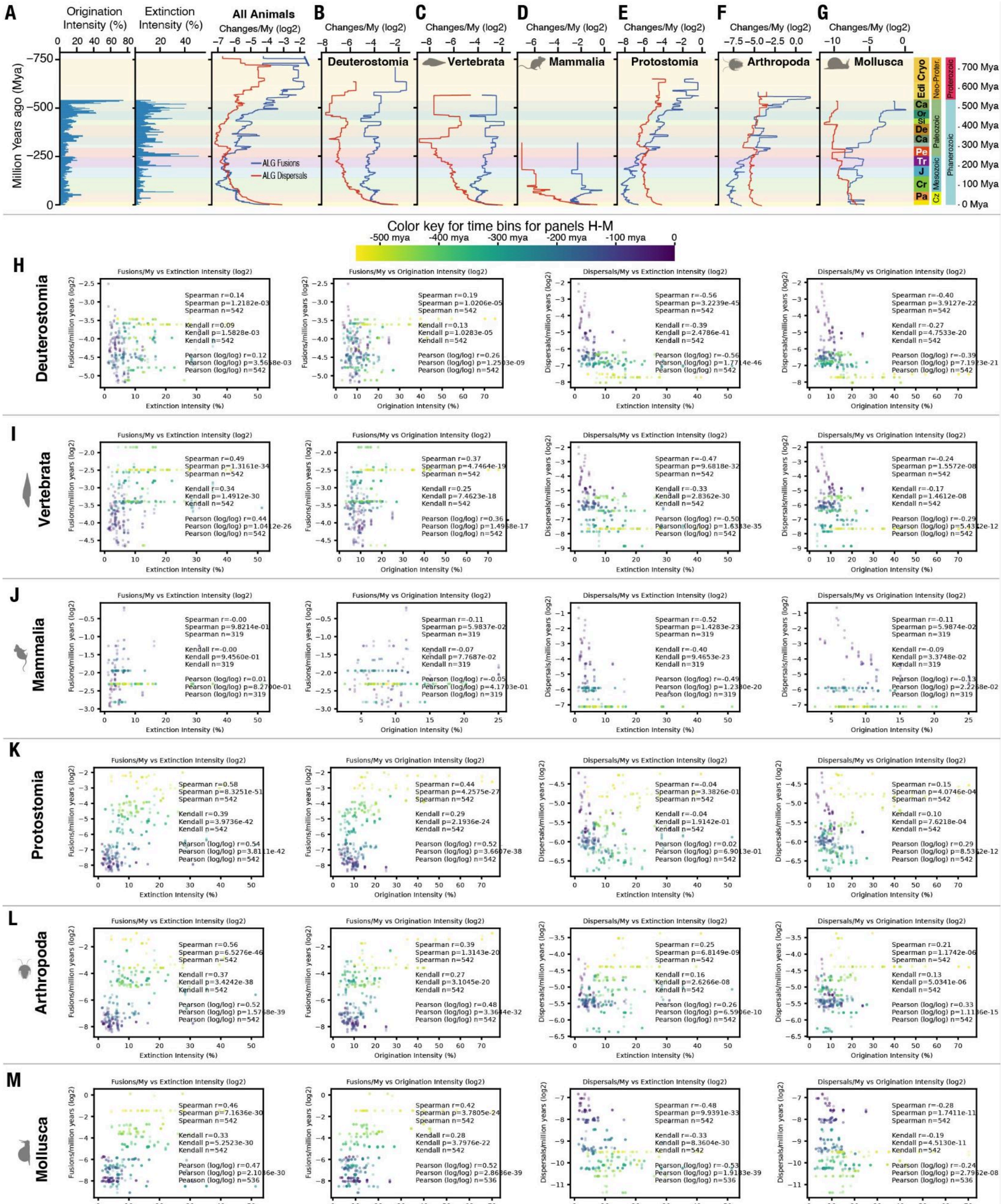
A
Origination Intensity (%)
Extinction Intensity (%)
All Animals
Changes/My (log2)
Million Years ago (Mya)
ALG Fusions
ALG Dispersals
B
Deuterostomia
C
Vertebrata
D
Mammalia
E
Protostomia
F
Arthropoda
G
Mollusca
Cryo
Edi
Ca
Or
Si
De
Ca
Pe
Tr
J
Cr
Pa
Neo-Proter.
Paleozoic
Mesozoic
Cz
Proterozoic
Phanerozoic
700 Mya
600 Mya
500 Mya
400 Mya
300 Mya
200 Mya
100 Mya
0 Mya
Color key for time bins for panels H-M
-500 mya
-400 mya
-300 mya
-200 mya
-100 mya
H
Deuterostomia
I
Vertebrata
J
Mammalia
K
Protostomia
L
Arthropoda
M
Mollusca
Fusions/My vs Extinction Intensity (log2)
Fusions/My vs Origination Intensity (log2)
Dispersals/My vs Extinction Intensity (log2)
Dispersals/My vs Origination Intensity (log2)
Fusions/million years (log2)
Dispersals/million years (log2)
Extinction Intensity (%)
Origination Intensity (%)

Fig. S3. Timing and macroevolutionary correlates of ALG fusion and loss through dispersal rates. (**A**) This panel is an expansion of what is shown in Fig. 3D to G. The left two panels show origination intensity (%) and extinction intensity (%) by 1 million year bins, taken from Rohde & Muller (*31*). Right panel shows rates ALG events per million years on a $\log_2$ scale: fusions in blue and losses through dispersal in red. The y-axis is time before present (million years ago). Background color bands indicate geologic periods (key at right). (**B**) Deuterostomia. Same display as in **A** but showing ALG event rates for genomes from Deuterostomia only. Panels **C to G** depict the same as **A and B** but of genomes from the (**C**) Vertebrata, (**D**) Mammalia, (**E**) Protostomia, (**F**) Arthropoda, and (**G**) Mollusca. Trends per clade vary in that fusion or dispersal rates may change independently per clade over time. (**H to M**) Scatter plots relate ALG change rate (y-axis; $\log_2$ events per million years) to macroevolutionary intensity in the same 1-million year bins (x-axis; % from Rohde & Muller (*31*)). Points are colored by the age of the bin. Each dot is one time bin (n shown on panels). Reported stats are one-tailed Spearman's rank correlation coefficients r_s and *p*, Kendall's rank correlation coefficient, and the Pearson's correlation coefficient. Left-to-right, each panel shows the fusion rate versus extinction intensity, the fusion rate versus origination intensity, the dispersal rate versus extinction intensity, and the dispersal rate versus the origination intensity. (**H**) Interpretation for **Deuterostomia**: Fusions: weakly positively correlated with extinction intensity (r_s = 0.14) and origination intensity (r_s = 0.19). Dispersals: Moderately anticorrelated with species extinction (r_s = −0.56) and origination (r_s = −0.40). However, plotting by the age of the bin reveals that the anticorrelation is likely because of decreasing origination and extinction intensity over time. Similar patterns of correlation and anticorrelation are seen in (**I**) Vertebrata, (**J**) Mammalia, (**K**) Protostomia, (**L**) Arthropoda, and (**M**) Mollusca.

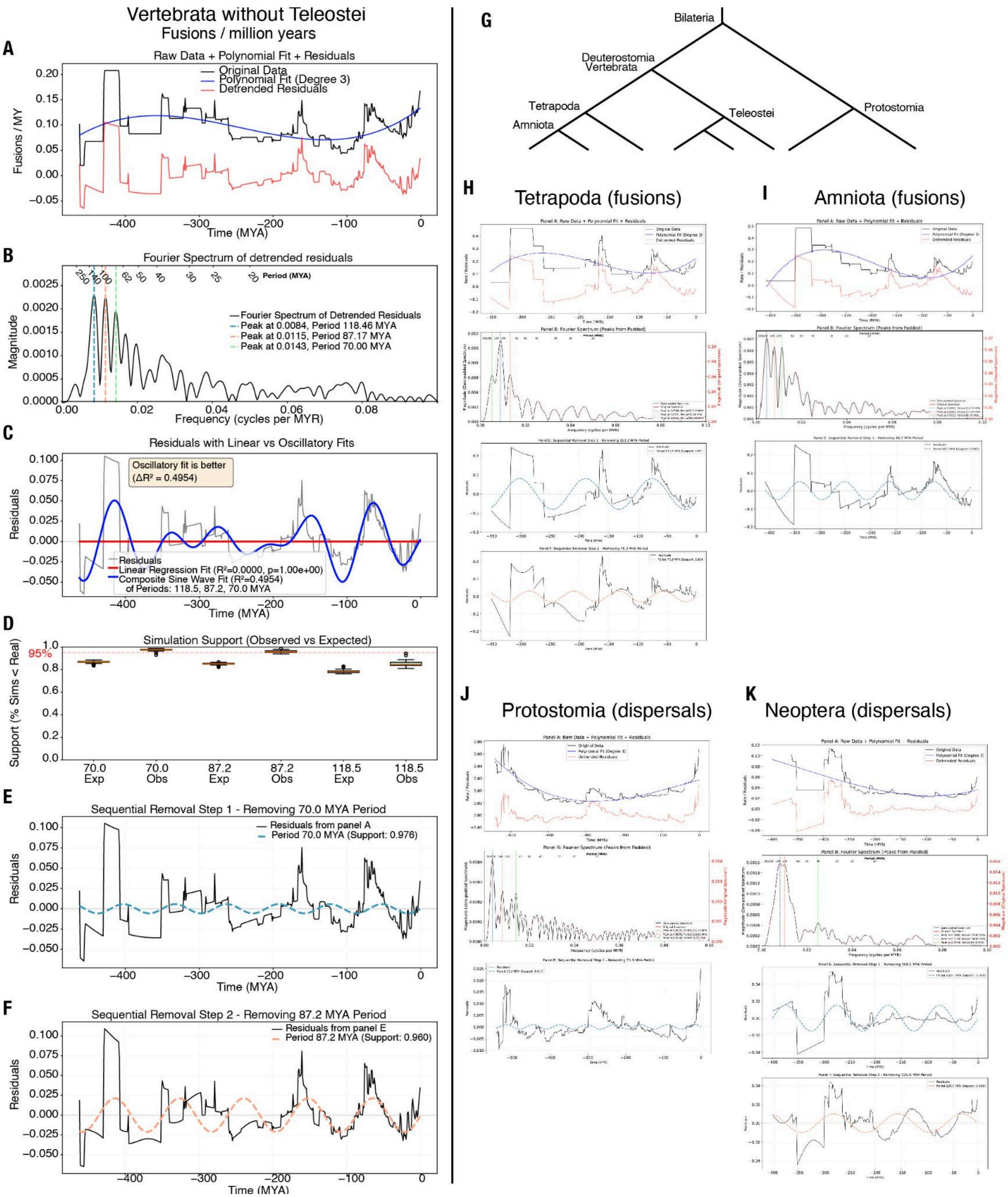
Vertebrata without Teleostei
Fusions / million years
A
Raw Data + Polynomial Fit + Residuals
Original Data
Polynomial Fit (Degree 3)
Detrended Residuals
Fusions / MY
Time (MYA)
B
Fourier Spectrum of detrended residuals
Period (MYA)
Fourier Spectrum of Detrended Residuals
Peak at 0.0084, Period 118.46 MYA
Peak at 0.0115, Period 87.17 MYA
Peak at 0.0143, Period 70.00 MYA
Magnitude
Frequency (cycles per MYR)
C
Residuals with Linear vs Oscillatory Fits
Oscillatory fit is better
(ΔR² = 0.4954)
Residuals
Linear Regression Fit (R²=0.0000, p=1.00e+00)
Composite Sine Wave Fit (R²=0.4954)
of Periods: 118.5, 87.2, 70.0 MYA
Time (MYA)
D
Simulation Support (Observed vs Expected)
95%
Support (% Sims < Real)
70.0 Exp
70.0 Obs
87.2 Exp
87.2 Obs
118.5 Exp
118.5 Obs
E
Sequential Removal Step 1 - Removing 70.0 MYA Period
Residuals from panel A
Period 70.0 MYA (Support: 0.976)
Residuals
Time (MYA)
F
Sequential Removal Step 2 - Removing 87.2 MYA Period
Residuals from panel E
Period 87.2 MYA (Support: 0.960)
Residuals
Time (MYA)
G
Bilateria
Deuterostomia
Vertebrata
Tetrapoda
Amniota
Teleostei
Protostomia
H
Tetrapoda (fusions)
I
Amniota (fusions)
J
Protostomia (dispersals)
K
Neoptera (dispersals)

Fig. S4. Periodicity analysis of BCnS ALG fusion or loss through dispersal events. (**A**) Rates of BCnS ALG fusions across the Metazoa in million-year bins, fitted with a quartic function. Black = original BCnS ALG fusions time series; blue = quartic polynomial fit to the original time series; red = detrended residuals. (**B**) Power spectrum of the series in panel **A**. Black = zero-padded discrete Fourier transform of the detrended series from panel A. The three strongest peaks are highlighted with vertical dotted lines. (**C**) A linear regression fit, and fit of an oscillatory model made from the three strongest peaks in panel B. (**D**) Distributions of supports from Monte Carlo simulations of different block sizes. The red support value is the fraction of simulated Fourier transforms with magnitudes less than the observed peak's magnitude. The individual points of the box-and-whisker plots are single simulations for number of randomized blocks, from 10 to 40. (**E**) The dominant peak supported by Monte Carlo simulations, fit to the detrended ALG fusion rate from panel A. (**F**) A sine wave of the second highest-supported period from the Monte Carlo simulations (orange dashed line). The black line is the detrended fusion rate from E with the fit sine wave from E subtracted. (**G**) Tree topology of subclades tested using this time-series analysis. (**H to K**) Fusions or dispersals for animal subclades, showing significant periods supported by Monte Carlo simulations. These clades were analyzed in the same fashion as vertebrates without teleosts (panels A-F).

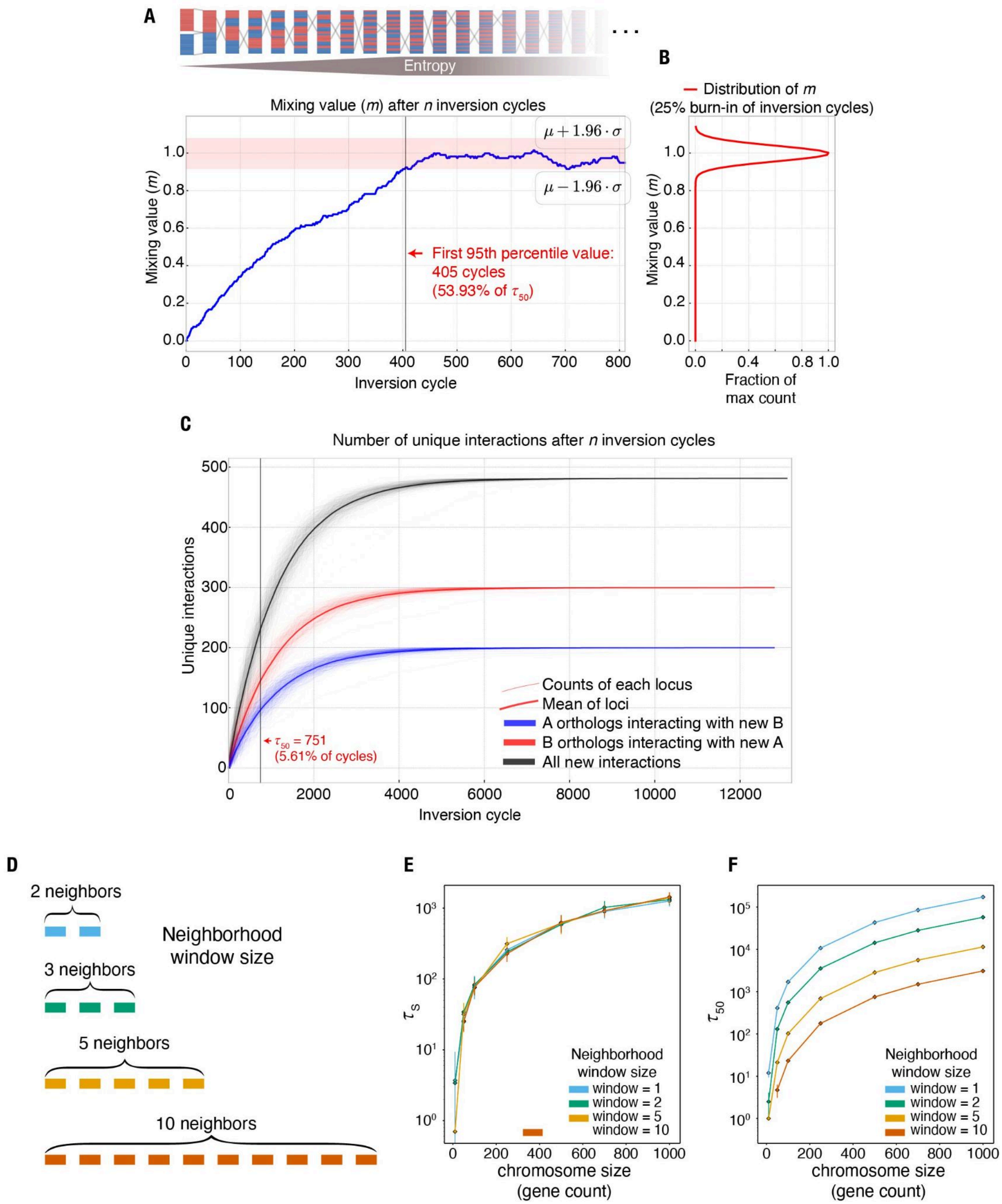

Fig. S5. Timescale of mixing and genomic neighborhood exploration. (**A**) Trace of the mixing value *m* (Materials and Methods) for two beads-on-a-string chromosomes after fusion. This simulation is for 300 genes in group A fusing with 200 genes in group B. (**B**) The mixing value *m* reaches the mass of the distribution (μ-1.96σ < *m* < μ+1.96σ of *m* after 25% burn-in) of a well-mixed state after 405 inversion cycles. (**C**) Counting the number of unique direct (nearest neighbor) interactions or pairs during the simulation. For the gene and window parameters of panel A, half of all possible neighborhoods have been explored only after 751 inversion cycles.

(**D**) The window size parameter changes the window size of genes that can be considered for new interactions. A small number of neighbors may best model genomes with cis-regulation, while a large number of neighbors may best model genomes with gene regulation grouped by TADs. (**E**) Window size has little effect on the amount of time it takes for genes to fully explore the number of possible combinations. Error bars are the standard deviations of 10 simulations. (**F**) More practically, however, the time to explore half or more of the combinations is reduced exponentially as the window size increases.

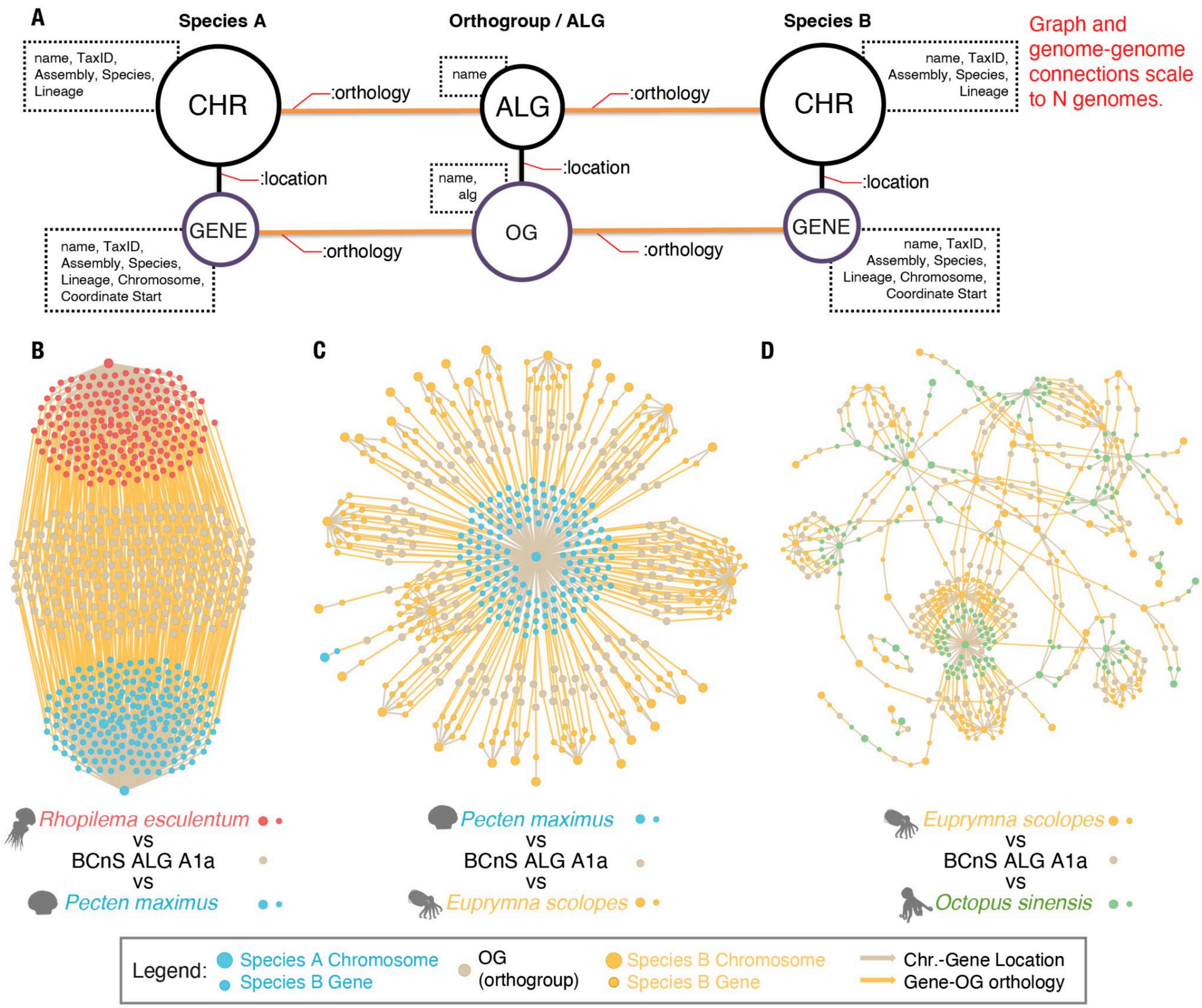

Fig. S6. Graph representation of orthology and genome context across species. This figure depicts the graph database encoding the relationships between the 4,454 species with chromosome-scale genomes in our dataset. **(A)** Data model used in the Neo4j database. Nodes represent biological entities: chromosome (CHR), gene (GENE), orthogroup (OG), and ancestral linkage group (ALG). Solid "location" edges (relationships) connect a feature (node) to the chromosome on which it resides within a genome. "Orthology" edges connect genes to orthogroups (OGs). Example node properties stored in the database (e.g., taxid, assembly, species, lineage, genomic coordinates) are indicated in dashed boxes. (**B**) This subgraph shows the genomic relationship between the ALG A1a and the genomes of the jellyfish *Rhopilema esculentum* (red) and the scallop *Pecten maximus* (blue). For each species, there are genes connected to (orthologous to) the BCnS ALG A1a OGs. These displayed genes are connected to the chromosome on which they exist for both species. The *Pecten* and *Rhopilema* chromosomes have been highly conserved since the myriazoan ancestor, and retain single chromosomes for the

BCnS A1a ALG. Node size encodes entity type, as in panel A, and as shown in the legend: large nodes are chromosomes (CHR), medium gray nodes are orthogroups (OG), small nodes are genes (GENE). (**C**) *Pecten maximus* and the Hawaiian bobtail squid *Euprymna scolopes*: There are multiple chromosomes in the squid genome that contain genes from ALG A1a, reflecting the complex rearrangements of BCnS ALGs in the evolutionary history of cephalopods since the common ancestor of all mollusks. (**D**) *Euprymna scolopes* versus *Octopus sinensis*: *Octopus* and other species within Octopodiformes have undergone their own complex genome rearrangements, and this is reflected in the complex ALG A1a graph between *Euprymna scolopes* (squids, Decapodiformes) and *Octopus sinensis* (octopuses, Octopodiformes). Together these networks show how the graph captures both orthology and genomic context, enabling qualitative assessment of one-to-one versus split linkage relationships across species.

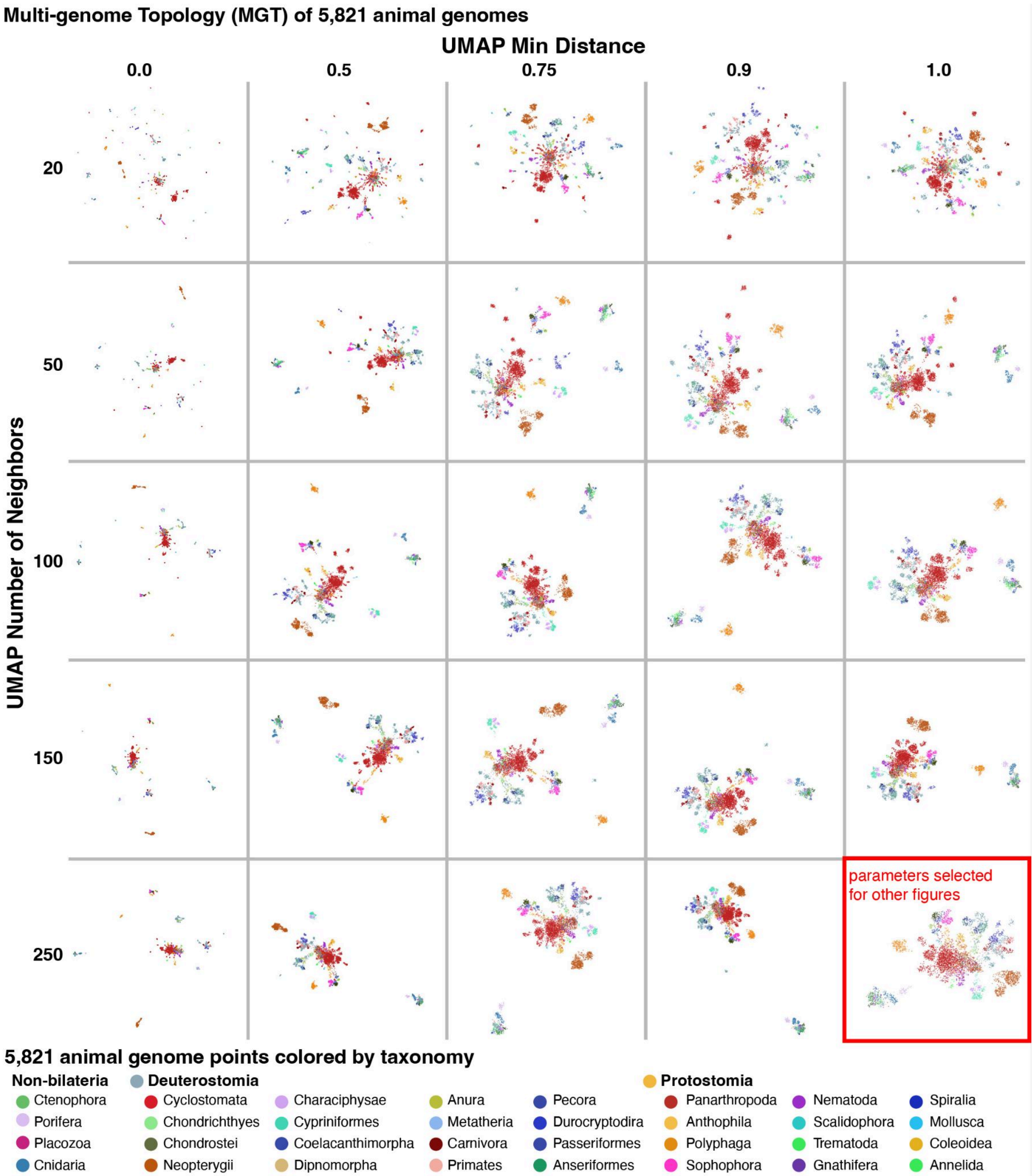

Multi-genome Topology (MGT) of 5,821 animal genomes
UMAP Min Distance
0.0
0.5
0.75
0.9
1.0
UMAP Number of Neighbors
20
50
100
150
250
parameters selected for other figures
5,821 animal genome points colored by taxonomy
Non-bilateria
Ctenophora
Porifera
Placozoa
Cnidaria
Deuterostomia
Cyclostomata
Chondrichthyes
Chondrostei
Neopterygii
Characiphysae
Cypriniformes
Coelacanthimorpha
Dipnomorpha
Anura
Metatheria
Carnivora
Primates
Pecora
Durocryptodira
Passeriformes
Anseriformes
Protostomia
Panarthropoda
Anthophila
Polyphaga
Sophophora
Nematoda
Scalidophora
Trematoda
Gnathifera
Spiralia
Mollusca
Coleoidea
Annelida

Fig. S7. UMAP parameter space exploration. Each of these MGT UMAPs is a projection of the same 5,821 animal genomes using different parameters: from 20 to 250 number of neighbors (*n*), and from 0.0 to 1.0 minimum distance (*m*). Each genome point is colored by the specified taxonomic rank, defaulting to the higher ranks otherwise. While local distribution of species on UMAPs changes according to neighborhood and min distance parameters, the overall clade assignment and composition remain stable. Using a smaller number of neighbors results in clusters in which genomes cluster akin to lower taxonomic ranks (orders, families, genera), while using a larger *n* results in clusters reflective of higher taxonomic ranks (phyla, classes, orders). We selected the parameters $n = 250$, $m = 1.0$ to focus on pan-animal relationships depicted well given the high number of neighbors.

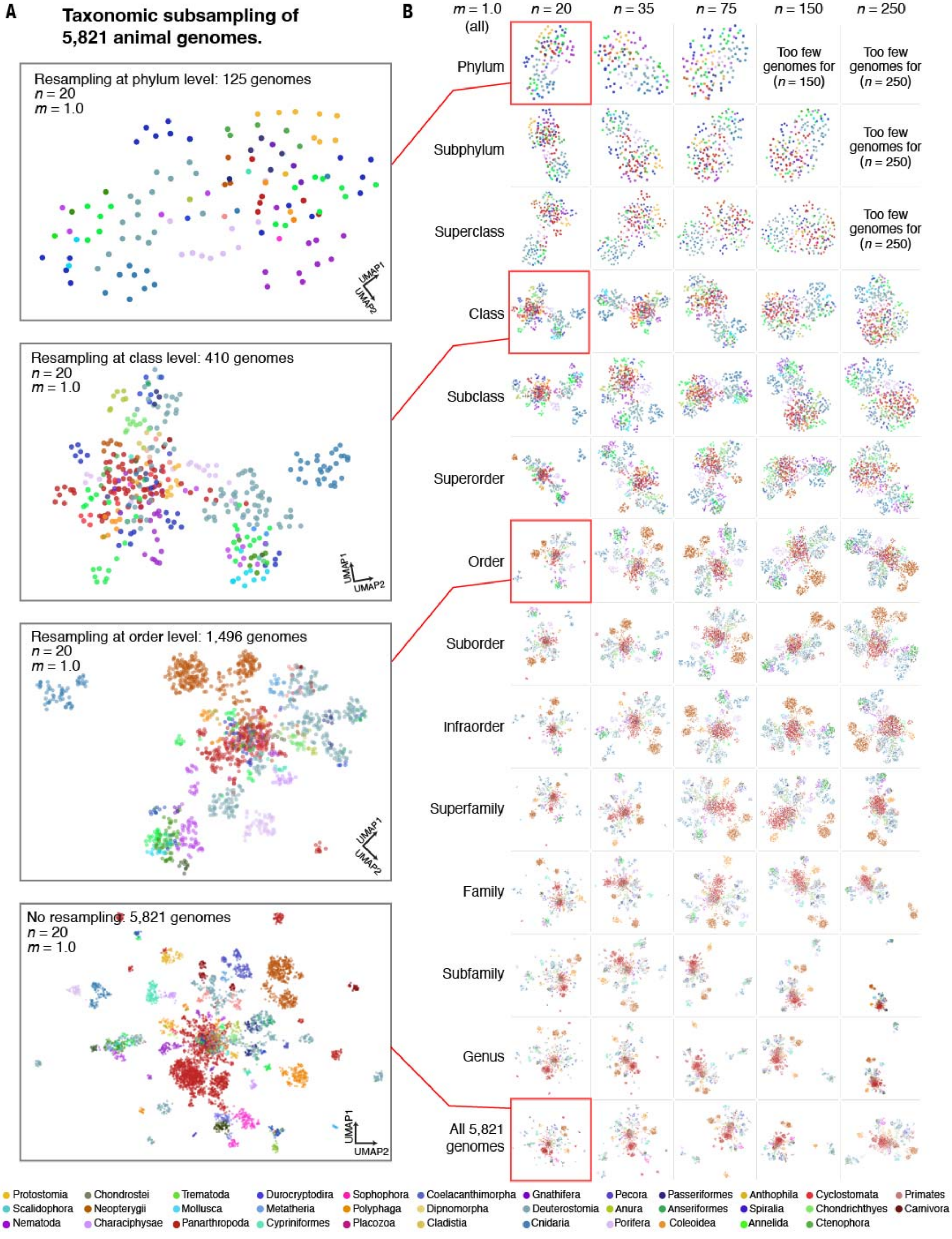
A
Taxonomic subsampling of 5,821 animal genomes.
Resampling at phylum level: 125 genomes
n = 20
m = 1.0
UMAP1
UMAP2
Resampling at class level: 410 genomes
n = 20
m = 1.0
Resampling at order level: 1,496 genomes
n = 20
m = 1.0
No resampling: 5,821 genomes
n = 20
m = 1.0
B
m = 1.0 (all)
n = 20
n = 35
n = 75
n = 150
n = 250
Phylum
Subphylum
Superclass
Class
Subclass
Superorder
Order
Suborder
Infraorder
Superfamily
Family
Subfamily
Genus
All 5,821 genomes
Too few genomes for (n = 150)
Too few genomes for (n = 250)
Protostomia
Scalidophora
Nematoda
Chondrostei
Neopterygii
Characiphysae
Trematoda
Mollusca
Panarthropoda
Durocryptodira
Metatheria
Cypriniformes
Sophophora
Polyphaga
Placozoa
Coelacanthimorpha
Dipnomorpha
Cladistia
Gnathifera
Deuterostomia
Cnidaria
Pecora
Anura
Porifera
Passeriformes
Anseriformes
Coleoidea
Anthophila
Spiralia
Annelida
Cyclostomata
Chondrichthyes
Ctenophora
Primates
Carnivora

Fig. S8. Taxonomic subsampling and MGT stability. This figure shows MGT UMAPs of the full 5,821-genome dataset, or of phylogenetically subsampled MGT UMAPs. Dots (individual genomes) are colored by the lowest taxonomic rank, shown in the legend. **(A)** MGT UMAPs using 20 neighbors and 1.0 minimum distance. Each box depicts the MGT UMAP from a different level of taxonomic subsampling (10 species per unique taxonomic unit). MGT UMAPs are rotated to maximize plot area. Under increasingly stringent subsampling, clade structure and long-range "trajectory" relationships remain visible. **(B)** Sensitivity of the manifold to neighborhood size across taxonomic subsampling levels. For each rank (rows; phylum → genus and the full set), MGT UMAPs are shown for n = 20, 35, 75, 150, and 250 (columns; m = 1.0 throughout). Red boxes highlight the panels shown at larger scale in panel A. Empty/annotated cells ("Too few genomes") indicate cases where the subsampled dataset was smaller than the requested number of neighbors (*n*). Overall, the same clade-specific clusters and projections persist across both taxonomic resampling and a wide range of MGT UMAP neighborhood sizes, indicating that MGT UMAP patterns are not driven by overrepresentation of particular lineages.

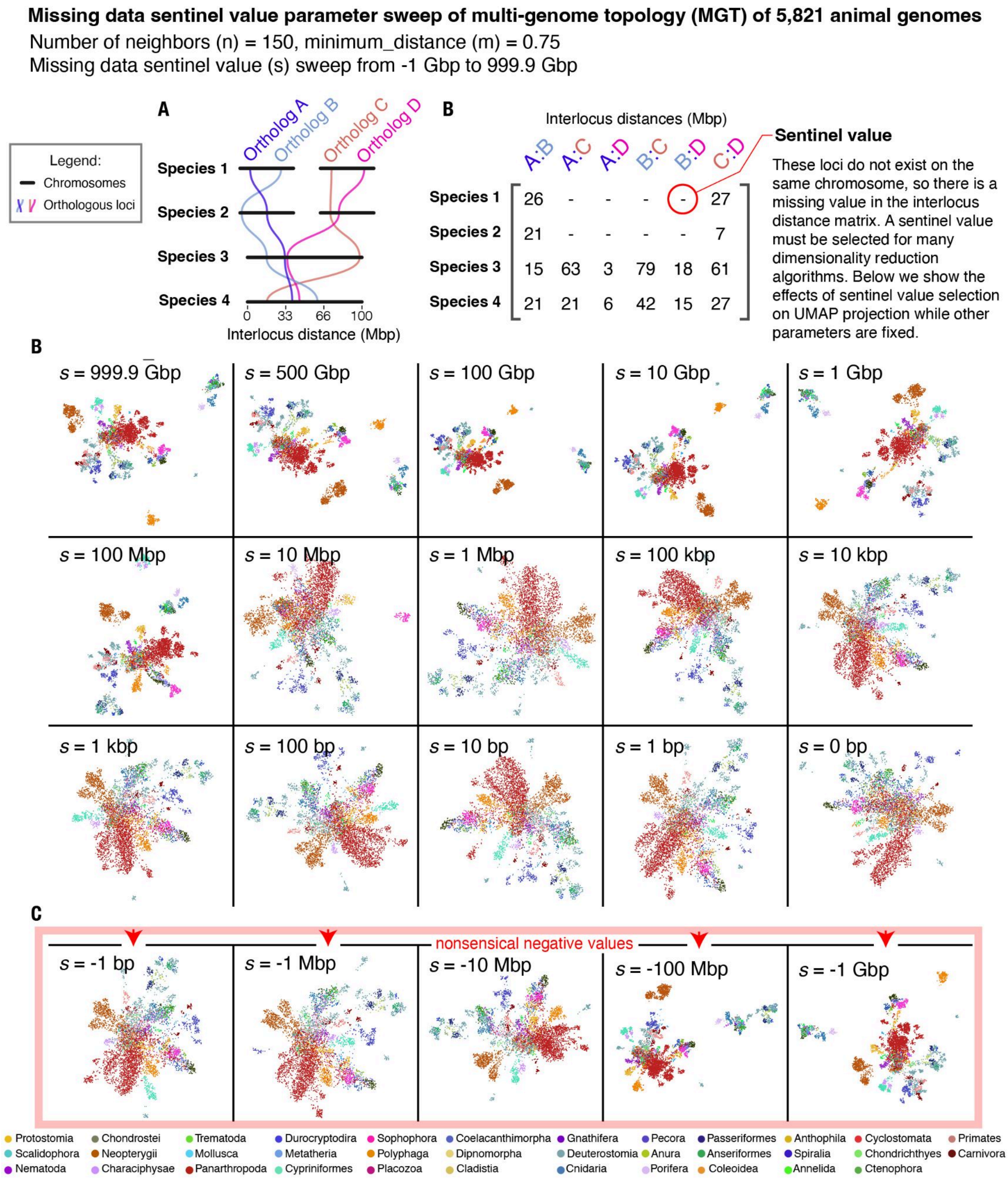

Missing data sentinel value parameter sweep of multi-genome topology (MGT) of 5,821 animal genomes
Number of neighbors (n) = 150, minimum_distance (m) = 0.75
Missing data sentinel value (s) sweep from -1 Gbp to 999.9 Gbp
A
Legend:
Chromosomes
Orthologous loci
Ortholog A
Ortholog B
Ortholog C
Ortholog D
Species 1
Species 2
Species 3
Species 4
0
33
66
100
Interlocus distance (Mbp)
B
Interlocus distances (Mbp)
A:B A:C A:D B:C B:D C:D
Species 1 26 - - - - 27
Species 2 21 - - - - 7
Species 3 15 63 3 79 18 61
Species 4 21 21 6 42 15 27
Sentinel value
These loci do not exist on the same chromosome, so there is a missing value in the interlocus distance matrix. A sentinel value must be selected for many dimensionality reduction algorithms. Below we show the effects of sentinel value selection on UMAP projection while other parameters are fixed.
B
s = 999.9 Gbp
s = 500 Gbp
s = 100 Gbp
s = 10 Gbp
s = 1 Gbp
s = 100 Mbp
s = 10 Mbp
s = 1 Mbp
s = 100 kbp
s = 10 kbp
s = 1 kbp
s = 100 bp
s = 10 bp
s = 1 bp
s = 0 bp
C
nonsensical negative values
s = -1 bp
s = -1 Mbp
s = -10 Mbp
s = -100 Mbp
s = -1 Gbp
Protostomia
Chondrostei
Trematoda
Durocryptodira
Sophophora
Coelacanthimorpha
Gnathifera
Pecora
Passeriformes
Anthophila
Cyclostomata
Primates
Scalidophora
Neopterygii
Mollusca
Metatheria
Polyphaga
Dipnomorpha
Deuterostomia
Anura
Anseriformes
Spiralia
Chondrichthyes
Carnivora
Nematoda
Characiphysae
Panarthropoda
Cypriniformes
Placozoa
Cladistia
Cnidaria
Porifera
Coleoidea
Annelida
Ctenophora

Fig. S9. Effect of the missing-data sentinel on multi-genome topology (MGT) UMAPs. (**A**) Schematic of four orthologous loci (A–D) on chromosomes from four species. Inter-locus distances are measured along chromosomes when loci co-occur on the same chromosome; otherwise the distance is undefined. (**B**) Example MGT interlocus distance matrix illustrating missing entries (dashes) when loci are on different chromosomes. These undefined distances are encoded by a single "sentinel" value *s* and must be converted to a numeric value in the MGT distance matrix. **(C)** UMAP projections of 5,821 animal genomes while sweeping the sentinel value *s* from very large to very small positive values (top to bottom, left to right; colors denote select clades). UMAP parameters were fixed (number of neighbors = 150, minimum distance = 0.75). Genome clustering and large-scale structure are stable for sentinel values greater than 100 million base pairs (Mb), however the best phylogenetic clustering occurs at numbers much larger than the largest chromosome in the dataset. When *s* is reduced toward or below typical intrachromosomal distances (distances ≤ ~10–100 kb), different-chromosome pairs are treated as near neighbors and the phylogenetic structure progressively weakens. **(D)** UMAPs with negative sentinel values (physically meaningless) illustrate that nonsensical encodings have similar effects to positive *s* of the same absolute value. Collectively these results demonstrate that the choice of a large sentinel enhances, but does not dictate, phylogenetic clustering in MGT UMAP space.

Genome Properties for the Multi-genome topology (MGT) UMAP, Number of Neighbors = 250, Min Distance = 1.0

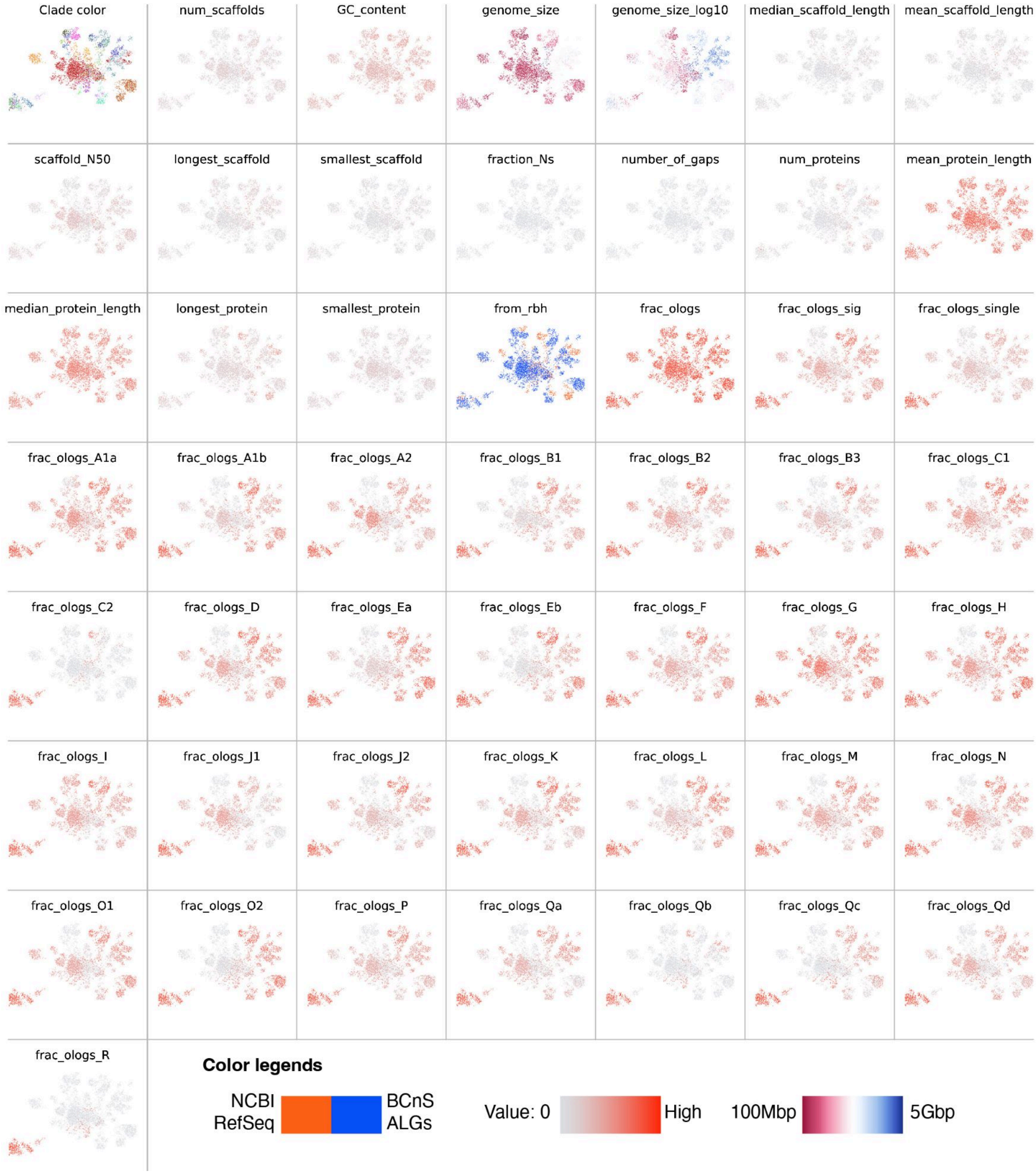

Fig. S10. Genomic property localization on UMAP species embedding. The UMAP visualization of genomes shows localized evolutionary history of basic genome properties and BCnS ALG changes. For each subplot, the scale is varied depending on the range of the values of all the genomes. The fields of the figure are: *clade_color*, each dot is colored by the clade code from **Fig. S7**; *num_scaffolds*, the number of scaffolds in the genome assembly fasta file; *GC_content*, the fraction of G or C nucleotides in the genome assembly fasta file; *genome_size*, the number of base pairs in the genome assembly fasta file; *genome_size_log10*, the same value as the genome_size column, but in log10; *median_scaffold_length*, the median length (base pairs) of all of the scaffolds in the genome assembly fasta file; *mean_scaffold_length*, same as the previous field, but the mean; *scaffold_N50*, the N50 of all the scaffolds in the genome assembly fasta file; *longest_scaffold*, the longest scaffold (base pairs) in the genome assembly fasta file; *fraction_Ns*, the number of Ns in the genome assembly fasta file divided by the total number of bases in the fasta file; *number_of_gaps*, the number of gaps, or continuous stretch of at least 10 N characters, in the genome assembly fasta file; *num_proteins*, the number of proteins present in the .chrom file for this genome; *mean_protein_length*, the mean length (amino acids) of the proteins present in the protein fasta file for this genome; *median_protein_length*, same as the previous field, but the median; *longest_protein*, the longest protein (amino acids) present in the protein fasta file for this genome; *smallest_protein*, the same as the last field, but the shortest protein; *from_rbh*, blue indicates that the genome was annotated with the 2361 BCnS ALG sequences (Materials and Methods), and orange indicates that the genome annotation was taken from NCBI; *frac_ologs*, the fraction of the 2361 BCnS ALG orthologs that were present on any scaffold in the genome; *frac_ologs_sig*, the fraction of the 2361 BCnS ALG orthologs that were present in a significant amount on any scaffold in the genome ($p \leq 0.05$, one-sided Bonferroni-corrected Fisher's exact test); *frac_ologs_single*, the fraction of the 2361 BCnS ALG orthologs significantly present on a single chromosome ($p \leq 0.05$, one-sided Bonferroni-corrected Fisher's exact test); *frac_ologs_{ALG name}*, similar to the previous two fields, this is the fraction of a single BCnS ALG that is significantly present ($p \leq 0.05$, one-sided Bonferroni-corrected Fisher's exact test) on one or more scaffolds in the genome.

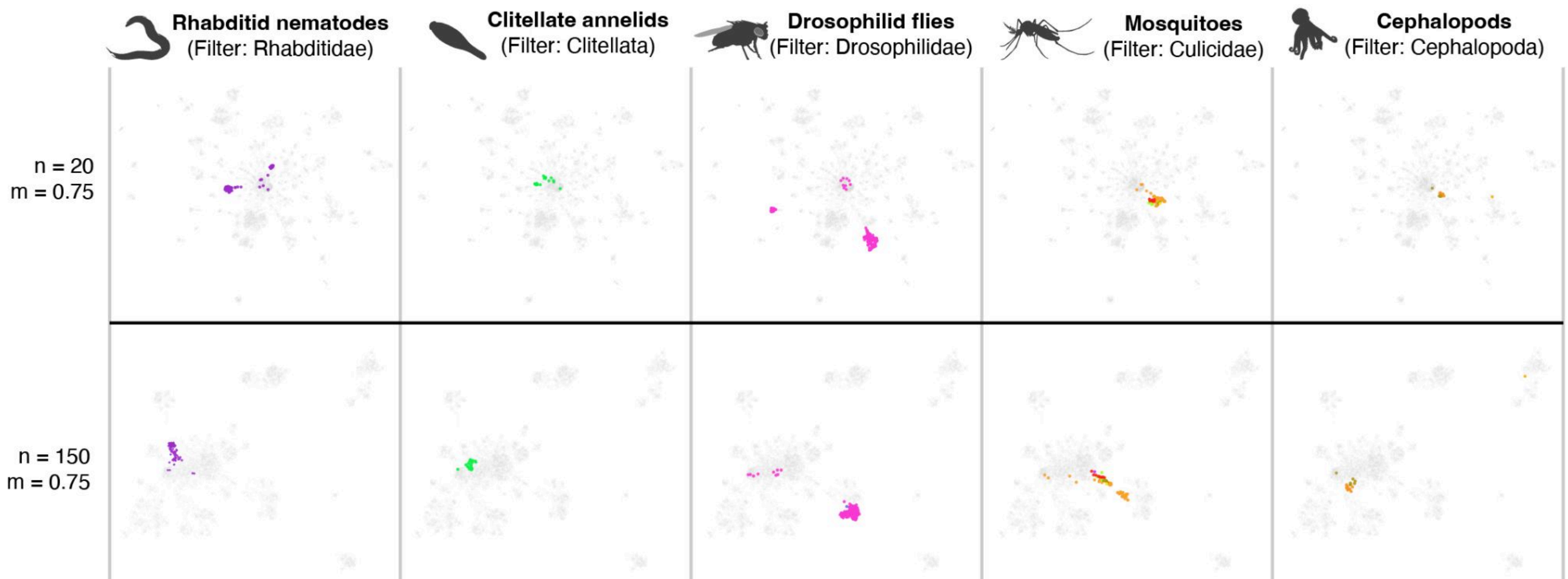

Fig. S11. **No evidence for a general "long-branch" attractor in multi-genome topology (MGT) space.** UMAP projections of the pan-animal MGT embedding shown with five derived clades highlighted (colored points) against the full dataset (gray points). Columns show, left to right: rhabditid nematodes (filter: Rhabditidae), clitellate annelids (filter: Clitellata), drosophilid flies (filter: Drosophilidae), mosquitoes (filter: Culicidae), and cephalopods (filter: Cephalopoda). The same underlying MGT distances are embedded with two substantially different UMAP parameterizations. Across both settings, each highlighted clade forms one or more discrete, clade-specific regions and does not collapse into a single shared "scrambled genome" cluster, arguing against a general long-branch-attraction-like, homoplasy-driven effect in MGT space. Some clades may appear adjacent under particular parameter choices (e.g., nematodes and clitellates at higher number of neighbors), but their clusters remain nonoverlapping and these configurations are not stable across parameters.

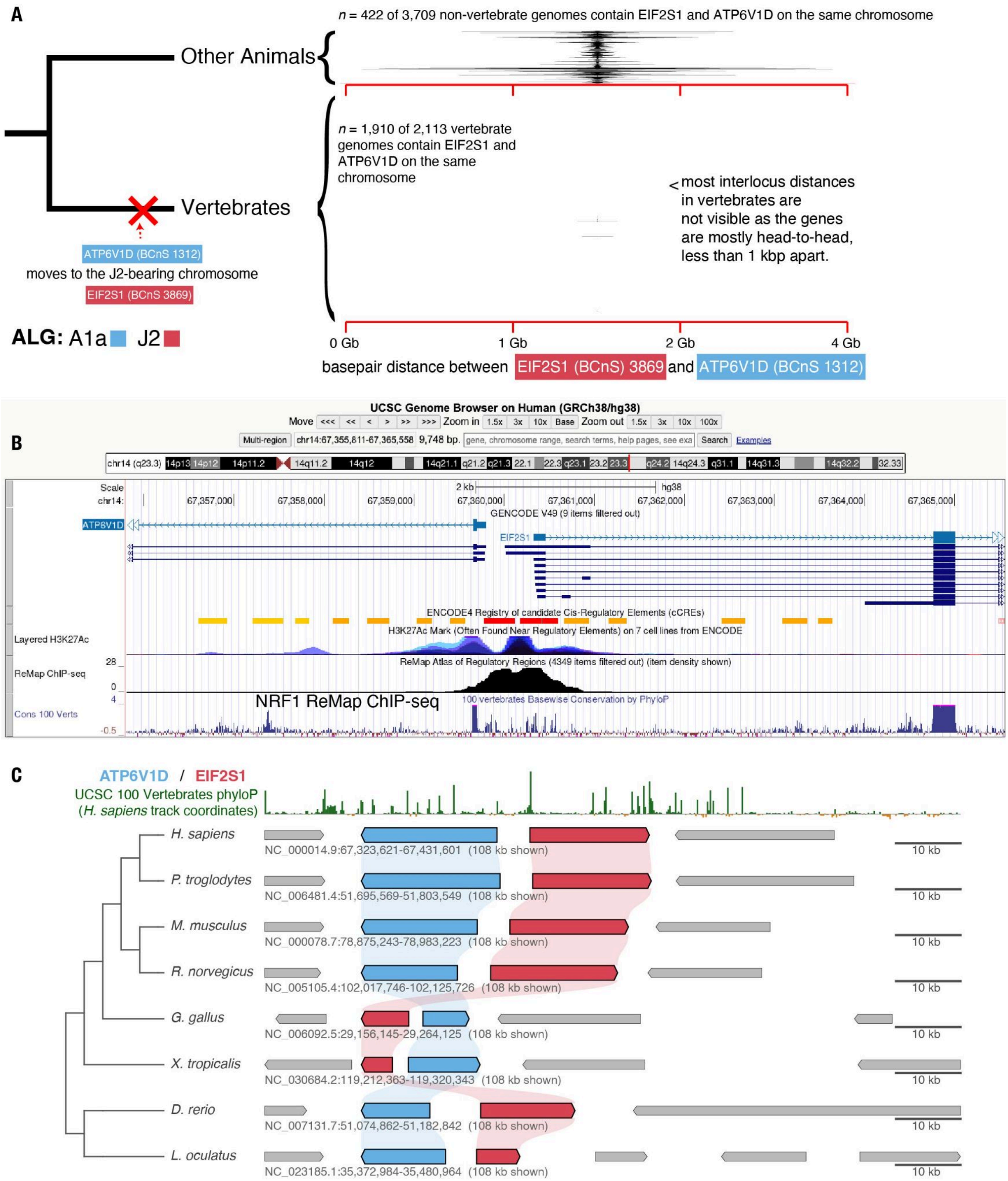

A
n = 422 of 3,709 non-vertebrate genomes contain EIF2S1 and ATP6V1D on the same chromosome
Other Animals
n = 1,910 of 2,113 vertebrate genomes contain EIF2S1 and ATP6V1D on the same chromosome
Vertebrates
< most interlocus distances in vertebrates are not visible as the genes are mostly head-to-head, less than 1 kbp apart.
ATP6V1D (BCnS 1312)
moves to the J2-bearing chromosome
EIF2S1 (BCnS 3869)
ALG: A1a J2
0 Gb
1 Gb
2 Gb
4 Gb
basepair distance between EIF2S1 (BCnS) 3869 and ATP6V1D (BCnS 1312)
B
UCSC Genome Browser on Human (GRCh38/hg38)
Move <<< << < > >> >>> Zoom in 1.5x 3x 10x Base Zoom out 1.5x 3x 10x 100x
Multi-region chr14:67,355,811-67,365,558 9,748 bp. gene, chromosome range, search terms, help pages, see exa Search Examples
chr14 (q23.3)
Scale
chr14:
2 kb
hg38
67,357,000
67,358,000
67,359,000
67,360,000
67,361,000
67,362,000
67,363,000
67,364,000
67,365,000
GENCODE V49 (9 items filtered out)
ATP6V1D
EIF2S1
ENCODE4 Registry of candidate Cis-Regulatory Elements (cCREs)
Layered H3K27Ac
H3K27Ac Mark (Often Found Near Regulatory Elements) on 7 cell lines from ENCODE
ReMap ChIP-seq
ReMap Atlas of Regulatory Regions (4349 items filtered out) (item density shown)
28
0
4
-0.5
Cons 100 Verts
NRF1 ReMap ChIP-seq
100 vertebrates Basewise Conservation by PhyloP
C
ATP6V1D / EIF2S1
UCSC 100 Vertebrates phyloP
(H. sapiens track coordinates)
H. sapiens
NC_000014.9:67,323,621-67,431,601 (108 kb shown)
P. troglodytes
NC_006481.4:51,695,569-51,803,549 (108 kb shown)
M. musculus
NC_000078.7:78,875,243-78,983,223 (108 kb shown)
R. norvegicus
NC_005105.4:102,017,746-102,125,726 (108 kb shown)
G. gallus
NC_006092.5:29,156,145-29,264,125 (108 kb shown)
X. tropicalis
NC_030684.2:119,212,363-119,320,343 (108 kb shown)
D. rerio
NC_007131.7:51,074,862-51,182,842 (108 kb shown)
L. oculatus
NC_023185.1:35,372,984-35,480,964 (108 kb shown)
10 kb

Fig. S12. **A vertebrate-specific close and stable locus pair.** (**A**) The genes ATP6V1D and EIF2S1 are extremely close and stable within Vertebrata. 90.9% of vertebrate genomes in our dataset had these genes on the same chromosome (*n* = 1,921 of 2,113), and were conserved head-to-head with one another (*n* = 1,910 of 2,113), often fewer than 1,000 base pairs apart. Comparatively, only 11% of genomes of non-vertebrate species have these genes on the same chromosome, and the interlocus distance has a high variance. These genes are located on separate chromosomes (and ALGs A1a and J2) in other animal clades outside Vertebrata, including in Ambulacraria (echinoderms and hemichordates) and cephalochordates (lancelets). (**B**) As an example species among vertebrates, ATP6V1D and EIF2S1 are head-to-head genes on chromosome 14 in the human genome, lying on opposite strands with their transcription start sites (TSS) less than 500 bp apart. The DNA around the TSS often has the active enhancer mark H3K27Ac in various human cell lines. Aggregate ChIP-seq data from experiments targeting the nuclear respiratory factor 1 (NRF1) transcription factor show binding in the noncoding sequence between ATP6V1D and EIF2S1. (**C**) Genome annotation tracks of eight vertebrate species that contain orthologs of ATP6V1D (blue) and EIF2S1 (red), as well as nearby annotated protein-coding genes (gray). Gene diagrams show the full annotation span from the transcription start site (TSS) to transcript end. 108 thousand base pairs (kb) are shown per species. The green genome track above the *H. sapiens* row shows the UCSC 100 Vertebrates phyloP conservation, peaking near the TSSs.

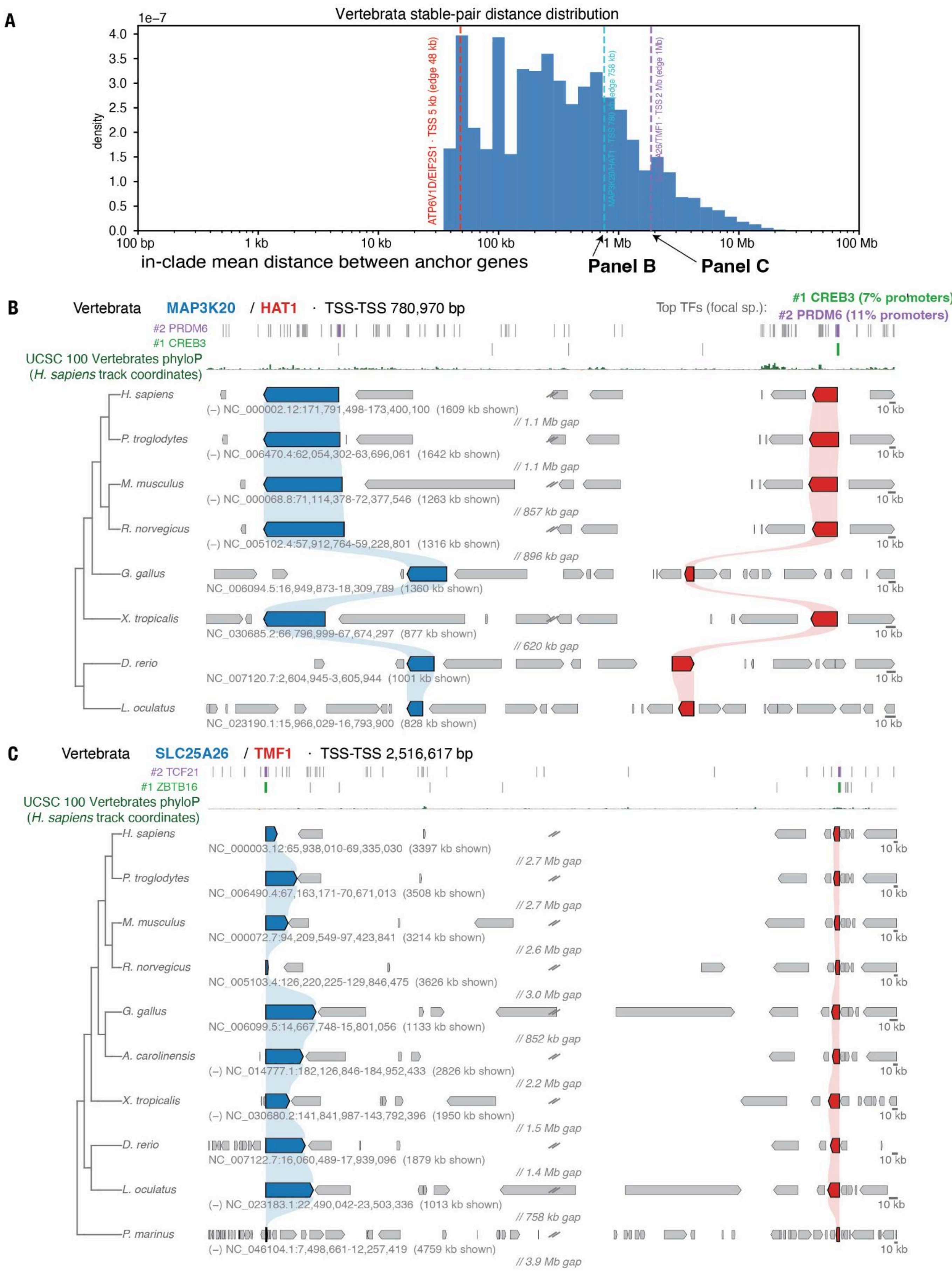

A
Vertebrata stable-pair distance distribution
1e−7
density
4.0
3.5
3.0
2.5
2.0
1.5
1.0
0.5
0.0
100 bp
1 kb
10 kb
100 kb
1 Mb
10 Mb
100 Mb
in-clade mean distance between anchor genes
ATP6V1D/EIF2S1 · TSS 5 kb (edge 48 kb)
Panel B
Panel C
B
Vertebrata MAP3K20 / HAT1 · TSS-TSS 780,970 bp
Top TFs (focal sp.):
#1 CREB3 (7% promoters)
#2 PRDM6 (11% promoters)
#2 PRDM6
#1 CREB3
UCSC 100 Vertebrates phyloP
(H. sapiens track coordinates)
H. sapiens
(−) NC_000002.12:171,791,498-173,400,100 (1609 kb shown)
// 1.1 Mb gap
P. troglodytes
(−) NC_006470.4:62,054,302-63,696,061 (1642 kb shown)
// 1.1 Mb gap
M. musculus
(−) NC_000068.8:71,114,378-72,377,546 (1263 kb shown)
// 857 kb gap
R. norvegicus
(−) NC_005102.4:57,912,764-59,228,801 (1316 kb shown)
// 896 kb gap
G. gallus
NC_006094.5:16,949,873-18,309,789 (1360 kb shown)
X. tropicalis
NC_030685.2:66,796,999-67,674,297 (877 kb shown)
// 620 kb gap
D. rerio
NC_007120.7:2,604,945-3,605,944 (1001 kb shown)
L. oculatus
NC_023190.1:15,966,029-16,793,900 (828 kb shown)
10 kb
C
Vertebrata SLC25A26 / TMF1 · TSS-TSS 2,516,617 bp
#2 TCF21
#1 ZBTB16
UCSC 100 Vertebrates phyloP
(H. sapiens track coordinates)
H. sapiens
NC_000003.12:65,938,010-69,335,030 (3397 kb shown)
// 2.7 Mb gap
P. troglodytes
NC_006490.4:67,163,171-70,671,013 (3508 kb shown)
// 2.7 Mb gap
M. musculus
NC_000072.7:94,209,549-97,423,841 (3214 kb shown)
// 2.6 Mb gap
R. norvegicus
NC_005103.4:126,220,225-129,846,475 (3626 kb shown)
// 3.0 Mb gap
G. gallus
NC_006099.5:14,667,748-15,801,056 (1133 kb shown)
// 852 kb gap
A. carolinensis
(−) NC_014777.1:182,126,846-184,952,433 (2826 kb shown)
// 2.2 Mb gap
X. tropicalis
(−) NC_030680.2:141,841,987-143,792,396 (1950 kb shown)
// 1.5 Mb gap
D. rerio
NC_007122.7:16,060,489-17,939,096 (1879 kb shown)
// 1.4 Mb gap
L. oculatus
(−) NC_023183.1:22,490,042-23,503,336 (1013 kb shown)
// 758 kb gap
P. marinus
(−) NC_046104.1:7,498,661-12,257,419 (4759 kb shown)
// 3.9 Mb gap
10 kb

Fig. S13. **Long-range vertebrate-specific stable locus pairs.** (**A**) Distribution of in-clade mean interlocus distances (mean_in) for 651 qualifying stable gene pairs within Vertebrata, plotted on a log10 base-pair axis from 100 bp to 100 Mb. While a small minority of pairs are tightly head-to-head (≤10 kb; including the EIF2S1 / ATP6V1D pair from **Fig. S12**), the bulk of vertebrate-stable pairs are separated by hundreds of kilobases to multiple megabases, with the modal distance near 1 Mb. Vertical dashed lines mark the focal pairs in this figure (e.g. MAP3K20 / HAT1 and SLC25A26 / TMF1). (**B**) The genes MAP3K20 and HAT1 are stably colocated within Vertebrata. They are usually arranged in a tandem same-strand orientation with a median transcription-start-site to transcription-start-site (TSS-TSS) distance of 781 kb across the eight vertebrate species shown here. In the human genome they sit on chromosome 2q31. Genome annotation tracks for eight vertebrates with orthologs of MAP3K20 (blue) and HAT1 (red); adjacent protein-coding genes in gray, lanes prefixed "(−)" shown in reverse-complement orientation. Above the H. sapiens row: the UCSC 100 Vertebrates phyloP conservation (green) and the two transcription factors with the strongest enrichment for binding both anchor promoters in human ChIP-seq data: CREB3 (#1; binds 7.0% of human promoters genome-wide) and PRDM6 (#2; 11.0%) — as colored peak ticks; ticks within 2 kb upstream of either anchor's TSS are highlighted to indicate promoter-proximal binding. (**C**) The genes SLC25A26 and TMF1 are stably colocated within Vertebrata at an even longer range (median TSS-TSS distance 2.52 Mb across all 10 vertebrate species sampled). They are arranged convergently on chromosome 3p14 in the human genome. Because the inter-anchor span exceeds 600 kb, each species lane displays two narrow flank windows (centered on the two anchor genes) joined by a "//" marker annotated with the per-species TSS-TSS distance; intervening genes between the two flanks are not shown. The phyloP conservation track and the top two transcription factors (#1 and #2) are shown as in panel B.

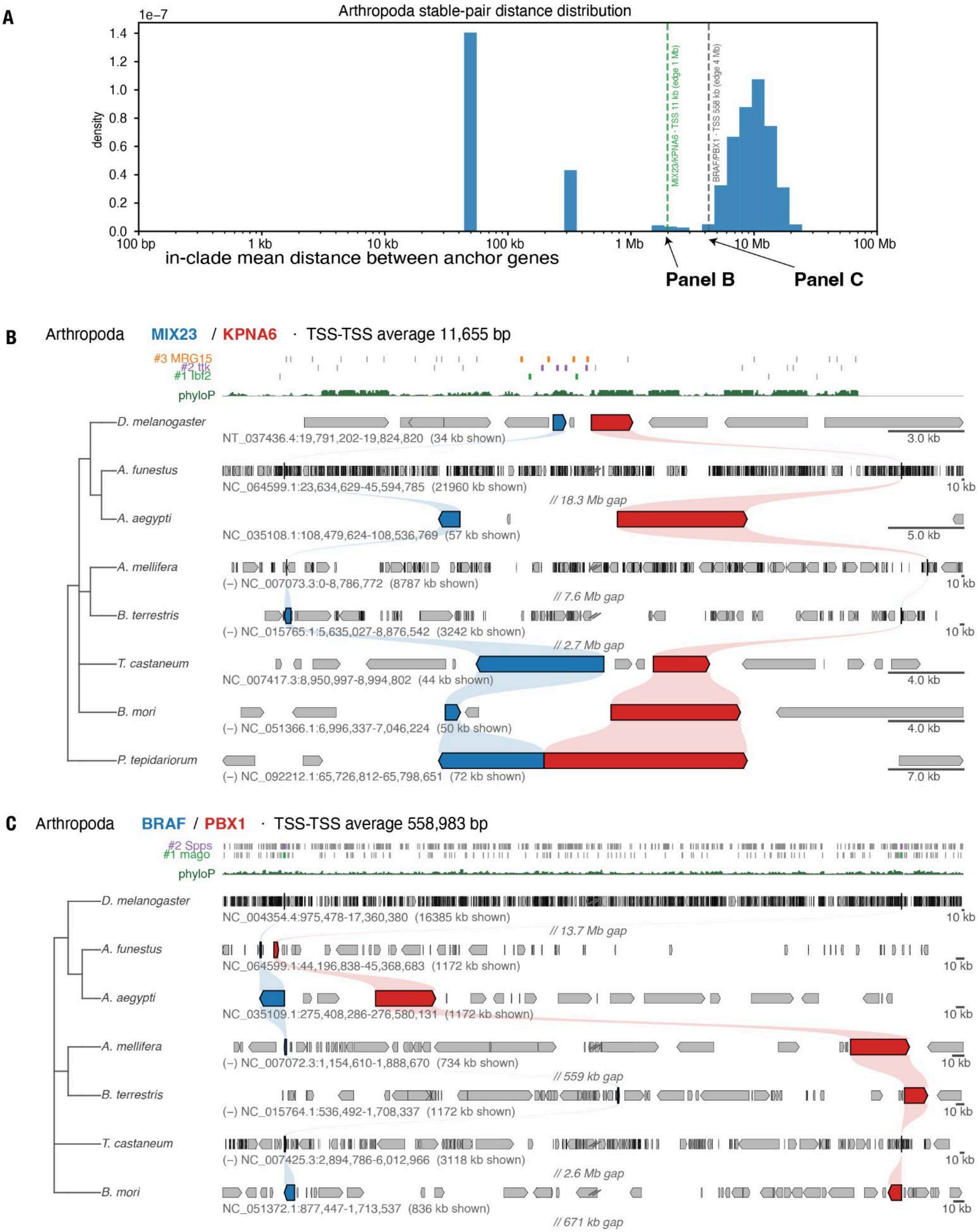
A
Arthropoda stable-pair distance distribution
1e−7
density
1.4
1.2
1.0
0.8
0.6
0.4
0.2
0.0
100 bp
1 kb
10 kb
100 kb
1 Mb
10 Mb
100 Mb
in-clade mean distance between anchor genes
MIX23/KPNA6 · TSS 11 kb (edge 1 Mb)
BRAF/PBX1 · TSS 558 kb (edge 4 Mb)
Panel B
Panel C
B
Arthropoda MIX23 / KPNA6 · TSS-TSS average 11,655 bp
#3 MRG15
#2 ttk
#1 Ibf2
phyloP
D. melanogaster
NT_037436.4:19,791,202-19,824,820 (34 kb shown)
3.0 kb
A. funestus
NC_064599.1:23,634,629-45,594,785 (21960 kb shown)
10 kb
// 18.3 Mb gap
A. aegypti
NC_035108.1:108,479,624-108,536,769 (57 kb shown)
5.0 kb
A. mellifera
(–) NC_007073.3:0-8,786,772 (8787 kb shown)
10 kb
// 7.6 Mb gap
B. terrestris
(–) NC_015765.1:5,635,027-8,876,542 (3242 kb shown)
10 kb
// 2.7 Mb gap
T. castaneum
NC_007417.3:8,950,997-8,994,802 (44 kb shown)
4.0 kb
B. mori
(–) NC_051366.1:6,996,337-7,046,224 (50 kb shown)
4.0 kb
P. tepidariorum
(–) NC_092212.1:65,726,812-65,798,651 (72 kb shown)
7.0 kb
C
Arthropoda BRAF / PBX1 · TSS-TSS average 558,983 bp
#2 Spps
#1 mago
phyloP
D. melanogaster
NC_004354.4:975,478-17,360,380 (16385 kb shown)
10 kb
// 13.7 Mb gap
A. funestus
NC_064599.1:44,196,838-45,368,683 (1172 kb shown)
10 kb
A. aegypti
NC_035109.1:275,408,286-276,580,131 (1172 kb shown)
10 kb
A. mellifera
(–) NC_007072.3:1,154,610-1,888,670 (734 kb shown)
10 kb
// 559 kb gap
B. terrestris
(–) NC_015764.1:536,492-1,708,337 (1172 kb shown)
10 kb
T. castaneum
(–) NC_007425.3:2,894,786-6,012,966 (3118 kb shown)
10 kb
// 2.6 Mb gap
B. mori
NC_051372.1:877,447-1,713,537 (836 kb shown)
10 kb
// 671 kb gap

Fig. S14. **Long-range arthropod-specific stable locus pairs.** (**A**) Distribution of in-clade mean interlocus distances (mean_in) for 612 qualifying stable gene pairs within Arthropoda. The bulk of arthropod-stable pairs are separated by hundreds of kilobases to tens of megabases. Vertical dashed lines mark the focal pairs in this figure (e.g., MIX23 / KPNA6 and BRAF / PBX1). (**B**) The genes MIX23 and KPNA6 are stably colocated within Arthropoda. They are usually arranged in a tandem same-strand orientation with a median transcription-start-site to transcription-start-site (TSS-TSS) distance of 11.7 kb across the 8 arthropod species shown here. In the *Drosophila melanogaster* genome they sit on chromosome 3L. Genome annotation tracks for eight arthropods with orthologs of MIX23 (blue) and KPNA6 (red); adjacent protein-coding genes in gray, lanes prefixed "(−)" shown in reverse-complement orientation. Above the *D. melanogaster* row: the UCSC 124 Insects phyloP conservation (green) and the three transcription factors with the strongest enrichment for binding both anchor promoters in *Drosophila* ChIP-seq data: Ibf2 (#1; binds 31.7% of fly promoters genome-wide), ttk (#2; 34.3%), and MRG15 (#3; 37.2%) as colored peak ticks; ticks within 2 kb upstream of either anchor's TSS are highlighted to indicate promoter-proximal binding. (**C**) The genes BRAF and PBX1 are stably colocated within Arthropoda at a longer range (median TSS-TSS distance 559 kb across seven species shown). They are arranged divergently on chromosome X in *D. melanogaster*. Genome annotation tracks are shown as in panel B; the phyloP conservation track and the top two transcription factors (#1 mago binding 9.2% of fly promoters; #2 Spps 21.6%).

Supplementary Table Captions, Tables S1-S17

Table S1. Sample information for the genomes used in this study. This table contains information relevant to the genomes used in this study. The information in each column is self-evident, with the exception of the following columns: **Column B** (sample): the '-'-delimited three-field string used to mark files for this genome in analysis. The first field is the genus and species name, with special characters and space characters removed. The second field is the NCBI taxid. The third field is the NCBI accession number, or other accession source, with the underscore '_' character removed. **Column C** (color): is the color used to plot the dot. **Column D** (taxid): the NCBI taxid. **Column E** (taxname): the name associated with the NBCI taxid. **Column F** (taxid_list): the NCBI taxid taxonomy path for this genome. **Column G** (taxid_list_str): the NCBI taxid taxonomy path for this genome, joined with ';' characters. **Column V** (UMAP1): the UMAP 1 index for the MGT approach with parameters 150 nearest neighbors, 0.9 minimum distance. **Column X** (frac_ologs): the fraction of the 2361 BCnS orthologs that are conserved on any chromosome in this genome. **Column Y** (frac_ologs_sig): the fraction of the 2361 BCnS orthologs that are significantly conserved ($p \leq 0.05$, one-sided Bonferroni-corrected Fisher's exact test), on any chromosome in the genome. **Column Z** (frac_ologs_single): the fraction of the 2361 BCnS orthologs whose ALGs are significantly present on a single chromosome in this genome ($p \leq 0.05$, one-sided Bonferroni-corrected Fisher's exact test). **Column AA** (frac_ologs_A1a): The fraction of BCnS orthologs from ALG A1a that are significantly present ($p \leq 0.05$, one-sided Bonferroni-corrected Fisher's exact test) on any chromosome in this genome. **Column BS** (from_rbh): TRUE if the genome was annotated using BCnS ALG ortholog protein sequences, FALSE if the annotation was retrieved from NCBI.

Table S2. **ALG presence, absence, and colocalization information.** This table contains the contents of `perspchrom.tsv`. Notable column contents are: **Column A** (sample): the three-field string representing the sample — also seen in **table S1**. **Columns C**-**AE** (A1a, D, …): the integer value of these columns is the number of chromosomes on which the BCnS ALG in the column name was significantly detected ($p \leq 0.05$, one-sided Bonferroni-corrected Fisher's exact test). **Columns AF**-**PU**: These columns contain a 1 if the two BCnS ALGs in the column header are present on the same chromosome, or 0 otherwise.

Table S3. **ALG presence, absence, plus changes inferred along the evolutionary tree.** This is the file `per_node_ALG_presence_fusions.tsv`, which contains the same information as **table S2** at the species level. However, there are rows that contain those values summed for every node in the phylogenetic tree. The additional column, 'changestrings', contains the inferred evolutionary history of chromosomal changes leading up to that node.

Table S4. **ALG dispersal quantification.** This contains the contents of the file called `allsamples.rbhstats.collated.df`. The float values shown in the cells are the fraction of BCnS orthologs belonging to each ALG that are significantly present ($p \leq 0.05$, one-sided Bonferroni-corrected Fisher's exact test) on any chromosome in the genome.

Table S5. **Inferred positions of chromosomal changes.** This is the `statsdf.tsv` file that contains the inferred positions of where chromosomal changes happened along the tree of

life. The column contents are as follows: **Column A** (target_taxid): the NCBI taxid is the node to which the branch leads. **Column B** (change): BCnS ALG names indicate that the BCnS ALG was dispersed on this branch. Tuples of two BCnS ALG names indicate that the two ALGs became colocalized on the same chromosome on this branch. **Column C** (counts): the number of species from which we inferred this change. **Column D** (number_of_samples_in_taxid): the total number of species present in this clade. **Column E** (change_frac_total): of all of the identical changes seen across the animal tree, what percent of them occur on this branch. **Column F** (frac_samples_w_this_taxid_w_this_change): the value of the 'counts' column divided by the value of the 'number_of_samples_in_taxid' column. **Column G** (target_taxid_name): The name of the NCBI taxid from the 'target_taxid' column.

Table S6. **Inferred rates of change per node.** For each clade, this table contains information detailing the inferred rates of chromosomal fusion and dispersal events, as well as the statistical correlation information with the changes only in the clade represented in that row. The phylogenetic tree is encoded in the 'parent', 'taxid', and 'children' nodes.

Table S7. **Inferred rates of change per evolutionary branch.** Each row contains the counts of evolutionary changes that occurred on each evolutionary branch.

Table S8. **Inferred rates of changes in the Metazoa per million years.** Rate units are changes per million years. Count units are integers. The underlying data are stored in each row in lists of float values. The 'age' column is the number of million years before present. There are columns that encode the previously reported species extinction and species origination rates (*31*).

Table S9. **Inferred rates of changes in the Panarthropoda per million years.** Uses the same column information as Table S8.

Table S10. **Inferred rates of changes in the Protostomia per million years.** Uses the same column information as Table S8.

Table S11. **Inferred rates of changes in the Vertebrata per million years.** Uses the same column information as Table S8.

Table S12. **Fourier transform of chromosomal change rates.** Key for the column 'Clade + Change': M=Metazoa, P=Protostomia, V=Vertebrata, F=ALG fusion, L=ALG loss through dispersal. For the column 'Observed or Expected (exponential)', the 'observed' means that the counts from the simulations were used to measure the significance, while 'expected' means that the estimated value from 'the assumption of the exponential form' was used to measure the significance. The values in the columns from 'Minimum support' to 'Median support' are 1 minus the false discovery rate. In other words, these values are the percent of simulations that have Fourier power spectrum magnitudes at the period of interest *less than* the magnitude of the peak from the real data. The column 'polynomial' is the degree of the polynomial that was fit to the original data for detrending.

Table S13. **Chromosome fusion with mixing simulation results.** Contains simulation results of fusion with mixing results with various chromosome sizes.

Table S14. **Table of simulated FWM events from real data.** The column 'Compositions' refers to the BCnS ALGs that are present on the chromosome in the 'Chromosome' column. The 'Synapomorphy of' column refers to the end terminus node (clade) of the evolutionary branch on which the two ALGs from the 'Composition' clade became fused and mixed. The column 'm' is the mixing value of those two BCnS ALGs on this chromosome. The 'number of cycles to reach m' column is the number of cycles in our mixing simulations that were required to reach the observed mixing value, *m*.

Table S15. **Summarized counts of unique pairs that define select clades.** Key for columns: **Column C** (num_genomes_in): the number of genomes from species in this clade in our dataset. **Column D** (num_genomes_out): the number of genomes in species outside of this clade in our dataset. **Column E** (num_pairs_unique_to_clade): the number of ortholog pairs that are only found in this clade, and not outside, in our dataset. This pair could be present in only one genome to be counted as present in this clade. **Column F** (num_pairs_highocc_all_types): the number of pairs that occur in at least 50% of the genomes in our dataset that are either stable in the clade, unstable in the clade, especially close in the clade, especially distant in the clade, or unique to the clade. **Column G** (num_pairs_unique_to_clade_highocc): the number of ortholog pairs that are unique to this clade and occur in at least 50% of the genomes in the dataset. **Column H** (num_pairs_close_in_clade_highocc): number of pairs that are present in at least 50% of the genomes in this dataset, and have a z-score of less than -2 for the log ratio of distances for this pair within this clade versus outside of this clade.

Table S16. Individual pairs that define each clade.

Note: Table S16 is available in the Dryad repository for this manuscript https://doi.org/10.5061/dryad.m905qfvbv.

Key for columns: **Column A** (nodename): The name of the clade for which this pair of loci is being considered. **Column B** (taxid): The NCBI taxid for the clade listed in column A. **Column C** (ortholog1): One of the orthologs from the BCnS ALGs in the pair. **Column D** (rbh1): The ALG to which ortholog1 belongs. **Column E** (ortholog2): The other ortholog from the pair. **Column F** (rbh2): The ALG to which ortholog2 belongs. **Column G** (close_in_clade): 1 if the pair is especially close in this clade, defined by having an occupancy_in of >50% and a z-score of less than -2 for mean_in_out_ratio_log_sigma. **Column H** (distant_in_clade): Same as close_in_clade, but the z-score must be greater than 2 in mean_in_out_ratio_log_sigma. **Column I** (stable_in_clade): Same as close_in_clade, but the z-score must be less than -2 in the column sd_in_out_ratio_log_sigma. **Column J** (unstable_in_clade): The z-score of this pair must be greater than 2 in the column sd_in_out_ratio_log_sigma. **Column K** (unique_to_clade): 1 if this pair only appears in this clade in the dataset, and not outside of the clade. **Column L** (pair): An index that uniquely describes the pair also recorded in columns D to F. **Column M** (notna_in): The number of genomes in this clade that had this pair present on the same chromosome. **Column N** (notna_out): The number of genomes outside of the clade that had this pair present on the same chromosome. **Column O** (mean_in): The mean distance between the two loci in this pair in all of the genomes in this clade. **Column P** (sd_in): The standard deviation of the distances between these loci in all the genomes in the clade. **Column Q** (mean_out): The

mean distance between the two loci in this pair in all of the genomes outside the clade. **Column R** (sd_out): The standard deviation of the distances between these loci in all the genomes outside the clade. **Column S** (occupancy_in): The fraction of genomes in this clade in the dataset that have this pair of loci on the same chromosome. **Column T** (occupancy_out): The fraction of genomes outside this clade that have this pair of loci on the same chromosome. **Column U** (num_species_in): The number of genomes in this clade in this dataset. **Column V** (num_species_out): The number of genomes outside of this clade in this dataset. **Column W** (sd_in_out_ratio): The ratio of standard deviations of distances inside this clade versus outside the clade. **Column X** (sd_in_out_ratio_log): The log column W, sd_in_out_ratio. **Column Y** (mean_in_out_ratio): The ratio of the mean distances of the loci in genomes in the clade versus outside the clade. **Column Z** (mean_in_out_ratio_log): The log of column Y, mean_in_out_ratio. **Column AA** (sd_in_out_ratio_log_sigma): The z-score of column X when considered against the distribution of column X values for the same clade. **Column AB** (mean_in_out_ratio_log_sigma): The z-score of column Z when considered against the distribution of column Z values for the same clade.

Table S17. Clade-specific Gene Ontology enrichment. Clade-specific Gene Ontology enrichment of the closest, most stable, and jointly close-and-stable unique pairs per clade. One GO term row per (clade × axis × N-threshold × sweep_namespace × GO term) reaching $q \leq 0.25$ (BH-FDR). *Columns are as follows*. **clade**: focal clade in the metazoan phylogeny; **axis:** ranking used to pick the top-N pairs feeding the enrichment; **foreground**: closeness (lowest clade-mean pair distance), stability (lowest within-clade pair-distance variance), or intersection (pairs in both top-N sets); **N_threshold:** N value of the top-N cut; **sweep_namespace:** GO sub-ontology over which the hypergeometric test was swept (all = union; BP/MF/CC = restricted to one branch); **go_id**, **go_name**, **go_namespace:** term identifier, name, and authoritative namespace from go-basic.obo. Hypergeometric parameters (one contiguous block): **foreground_hits_[k]**, **foreground_size_[n]**, **background_hits_[K]**, **background_size_[N]**, **fold_enrichment** = (k/n)/(K/N), **q_value** = BH-adjusted p. Supporting test columns: **ratio_in_study_[k/n]**, **ratio_in_pop_[K/N]** (verbatim string ratios), **p_value** (raw upper-tail hypergeometric), **correction_method** (fdr_bh, constant across rows). Contributing genes: **gene_ids:** Entrez GeneIDs of the k foreground genes annotated with this term (semicolon-joined). **gene_symbols:** HGNC symbols in the same order. **bcns_families:** BCnS ALG family IDs (*2*) of the foreground families that contributed each hit gene, in the same order; pipe-joined when a GeneID is represented in more than one BCnS family.

Supplementary File 1 Caption

Supplementary File 1, also called Metazoa.neighbors_250.mind_1.0.missing_large.bokeh.html, contains an interactive version of the multi-genome topology (MGT) plot. This is the same MGT plot depicted in **Fig. 4**, **Fig. 5**, and **fig. S10**. The UMAP parameters were *n_neighbors* = 250, *min_dist* = 1.0. This contains the full dataset of genomes used in this manuscript.

The initial view of the page displays the UMAP with some tools for searching for specific clades. Mousing over individual dots displays information about that genome.

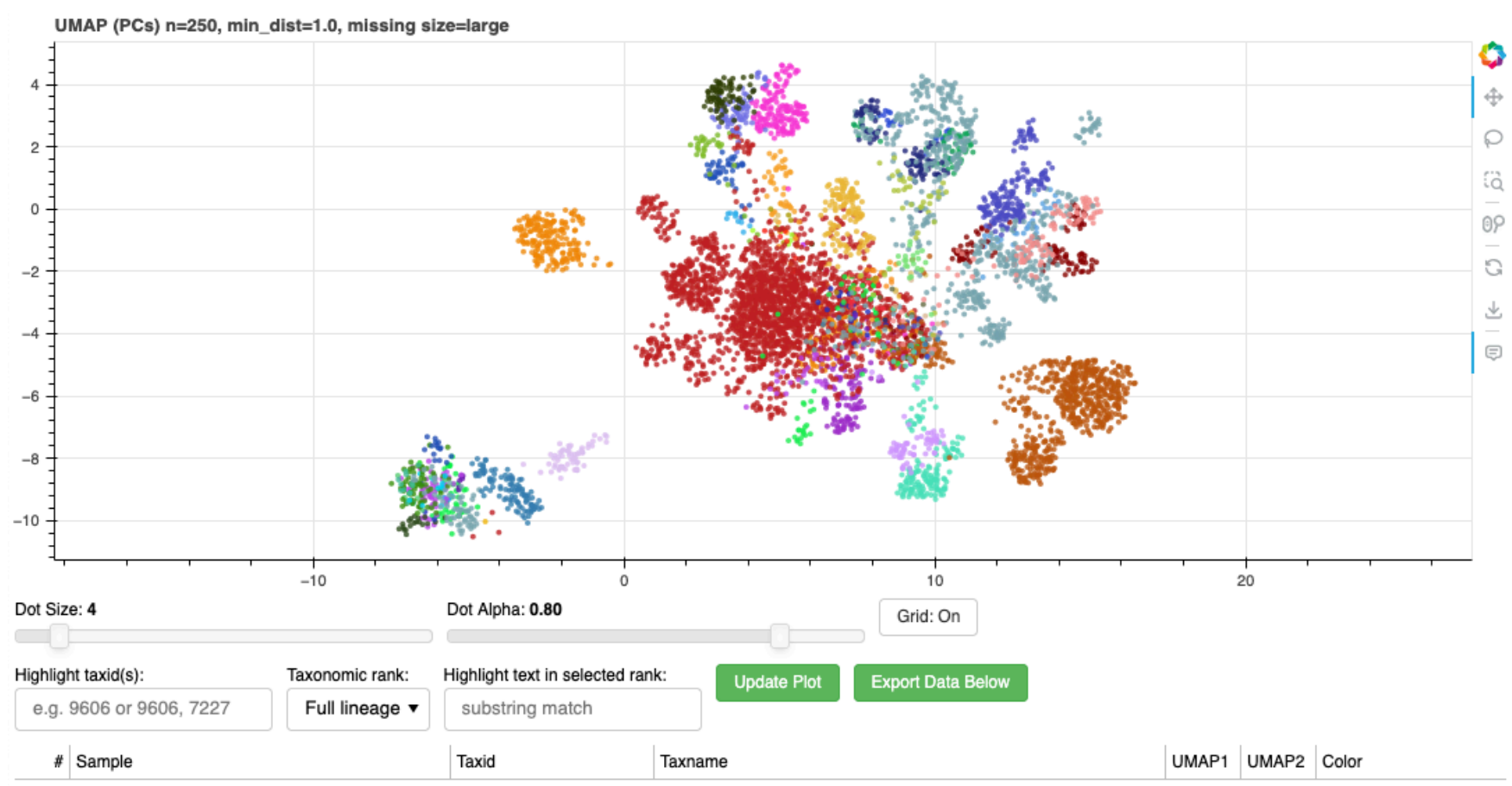

Typing in an NCBI taxonomy taxid, or the English name of a clade, will identify and highlight those dots and produce an interactive list of those genomes. These highlighted rows can be exported to a tab-delimited file.

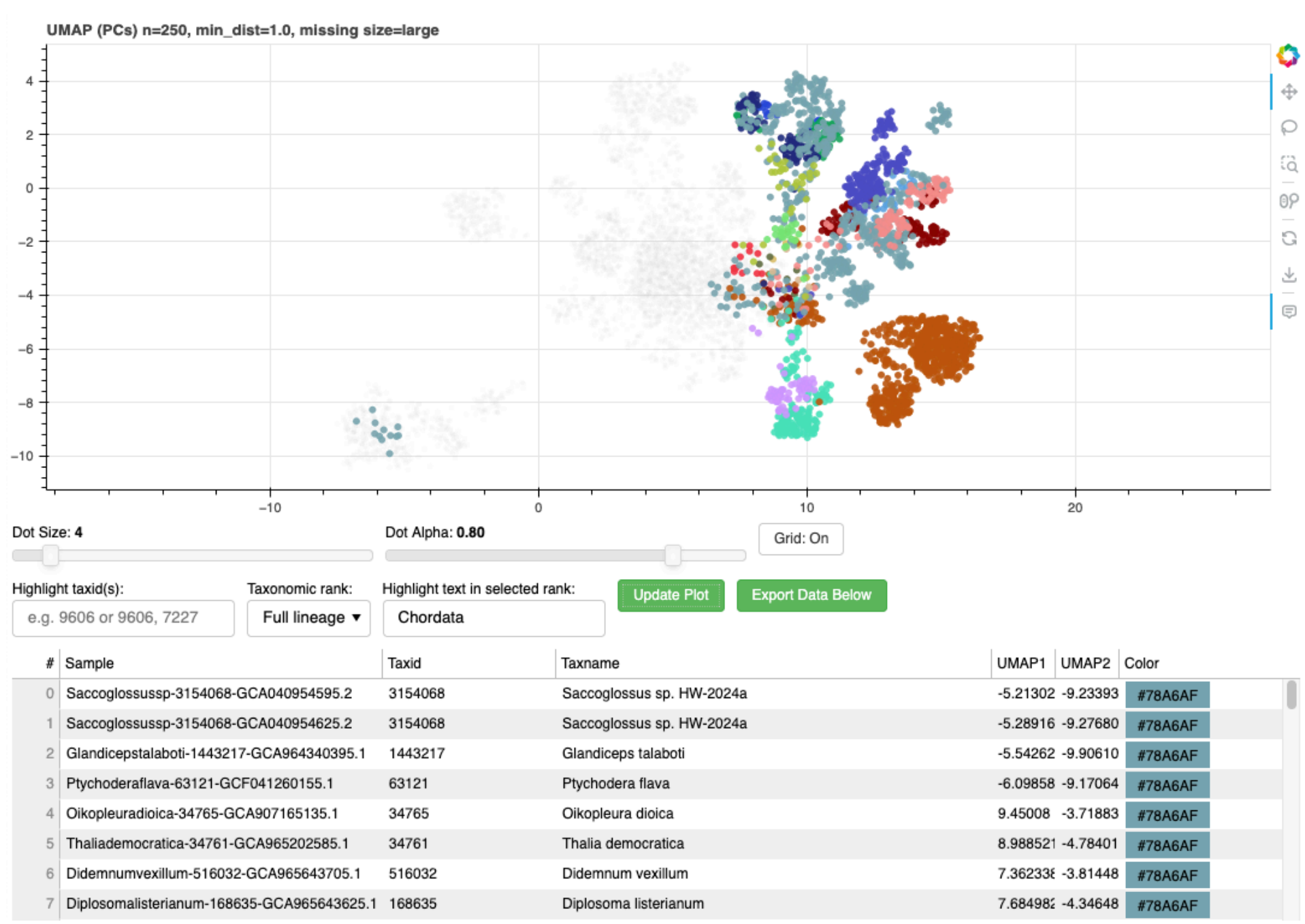

Supplementary Files Dryad Repository Content Guide

This contains additional analysis, including per-clade figures, that were not included in the main manuscript presentation because of space limitations.

Note: These files are available in the Dryad repository for this manuscript [*https://doi.org/10.5061/dryad.m905qfvbv*](https://doi.org/10.5061/dryad.m905qfvbv).

Directory structure:

- Events_over_time
    - Includes per-clade analyses of the fusions per million years and ALG dispersals per million years.
    - Files are named as follows: <clade_name>_<NCBI_taxid>_<plottype>.<ending>
    - The included files and their contents are:
        - *_changes_vs_intensity.pdf - plots of clade-specific ALG fusion or dispersal rates versus species origination or extinction rates.

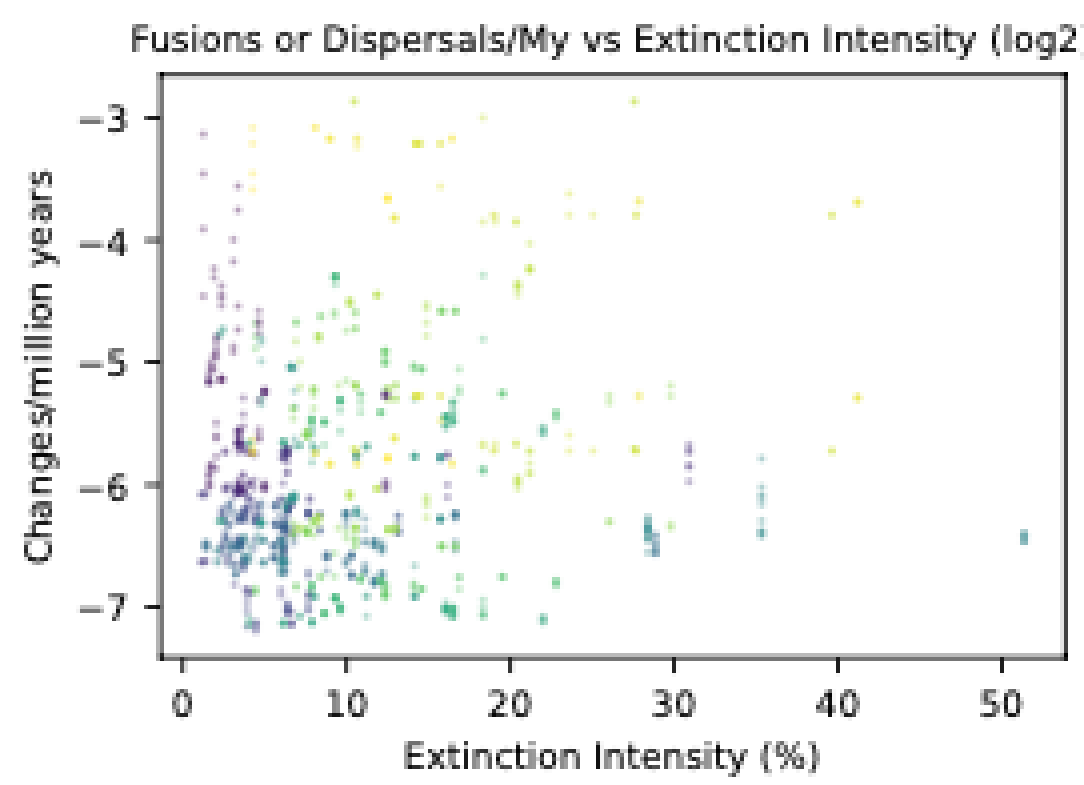

        - *_changes_versus_time.pdf - clade-specific plots of the ALG fusions or ALG dispersals over time.

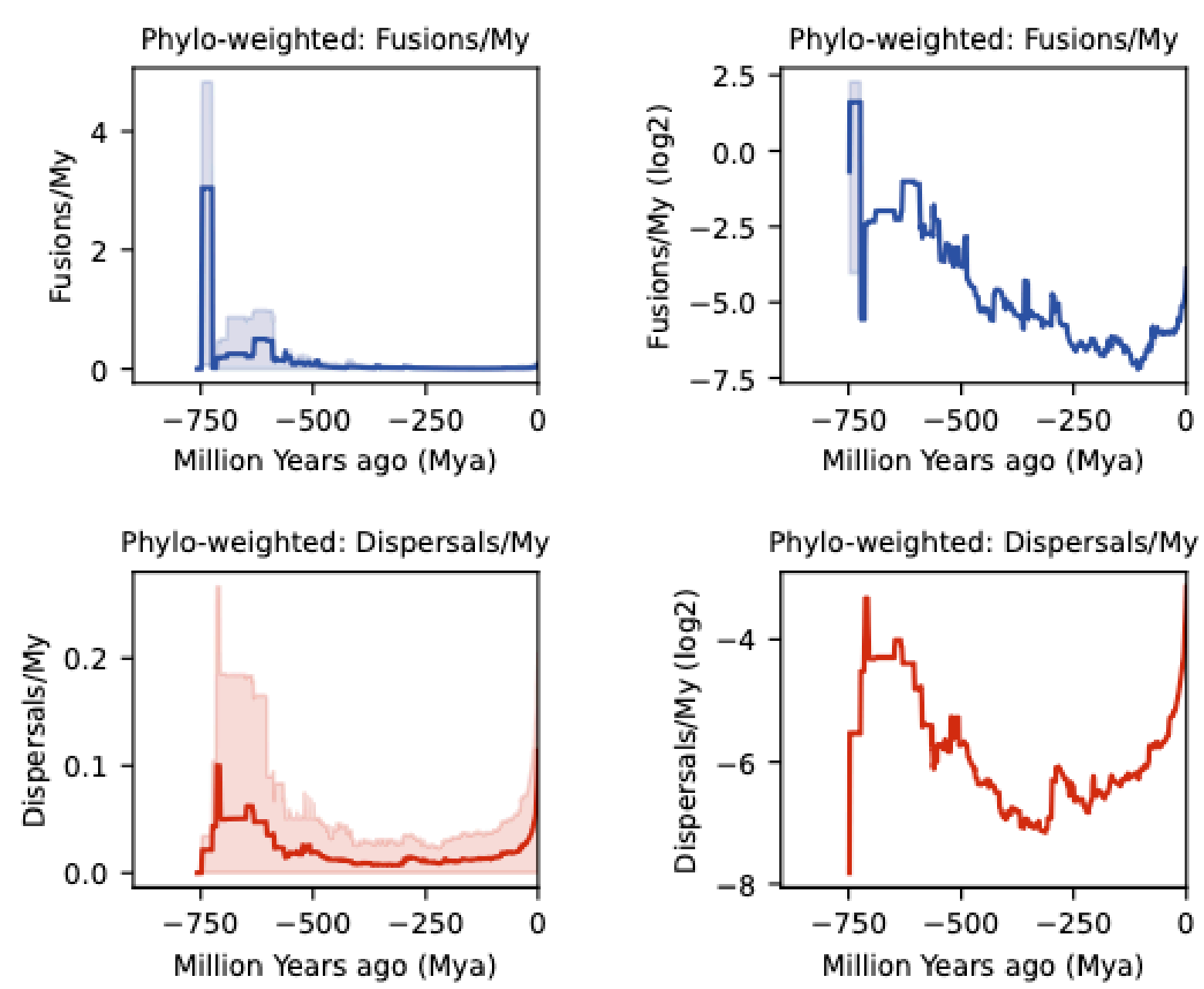

- *_event_count_conservation.pdf - the number of total events observed before and after phylogenetic weighting

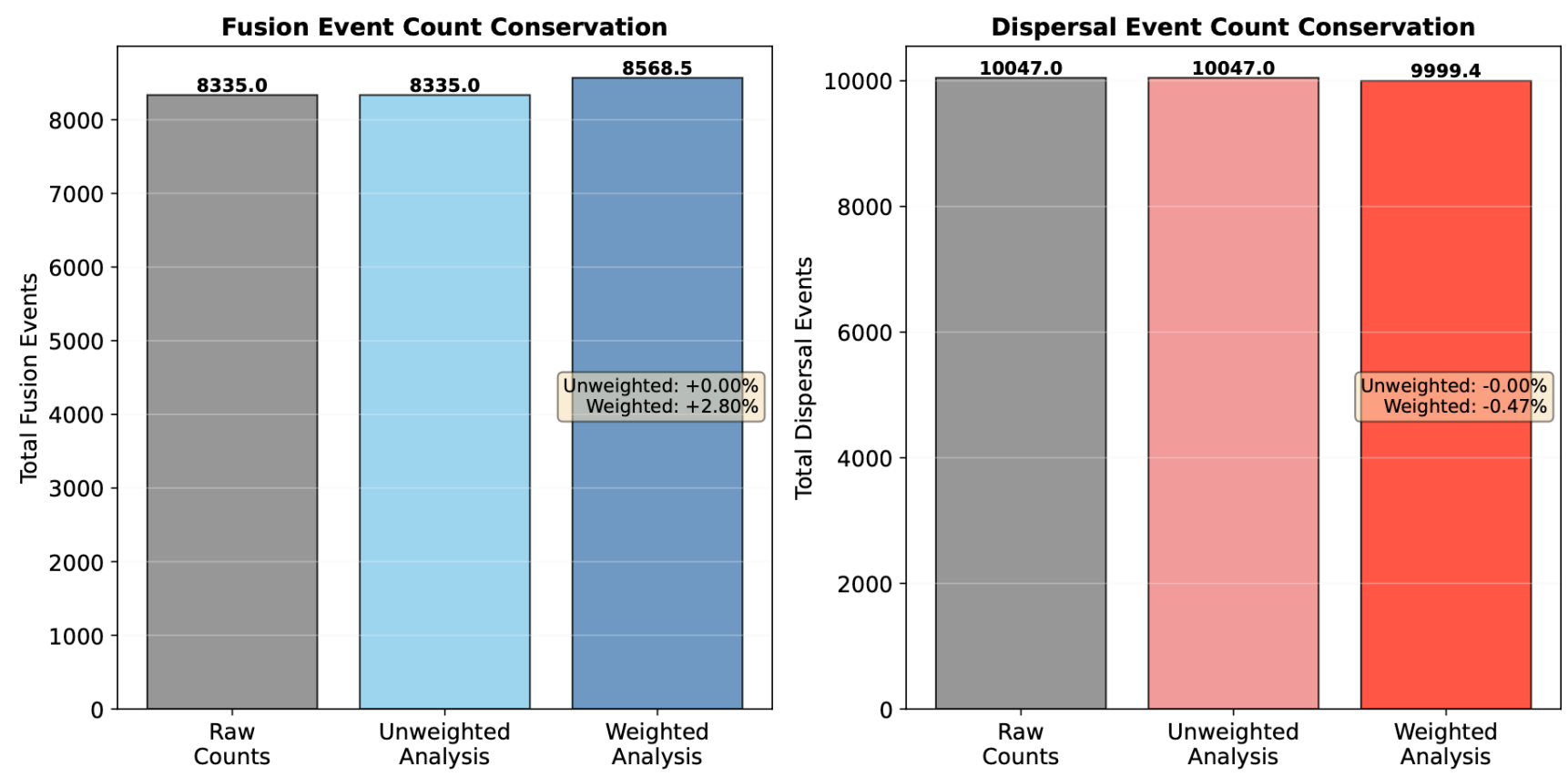

- *_phylogenetic_weighting_verification.pdf - shows the effects of phylogenetic weighting on the average rates.

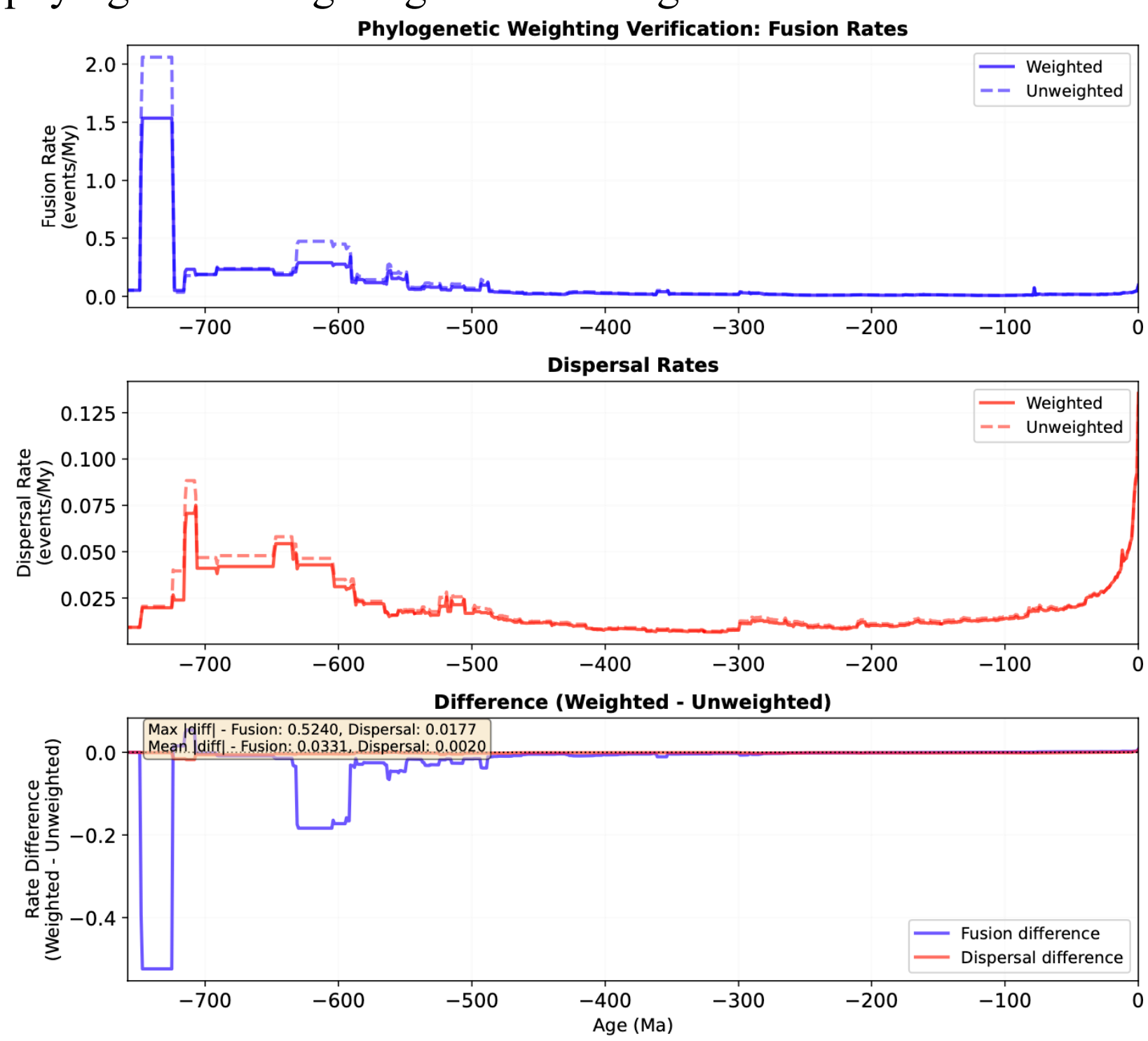

- *_phylogenetic_weights_diagnostic.pdf - shows the distribution of patristic-distance-summed phylogenetic weights of the whole tree for that

clade.

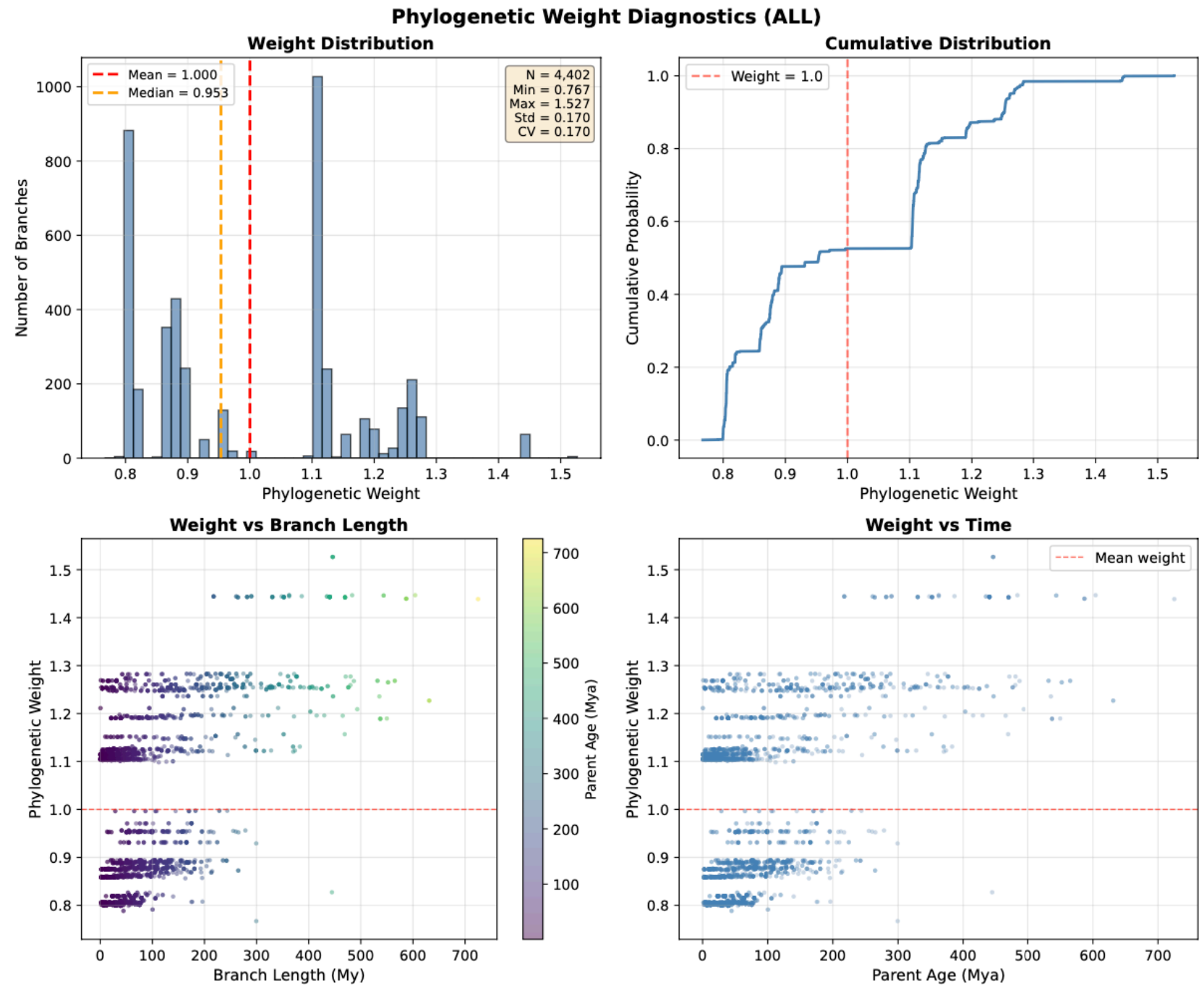

*_temporal_heatmap.pdf - a heatmap of the distribution of phylogenetic weights at time slices of million-year points along the tree for the clade under analysis.

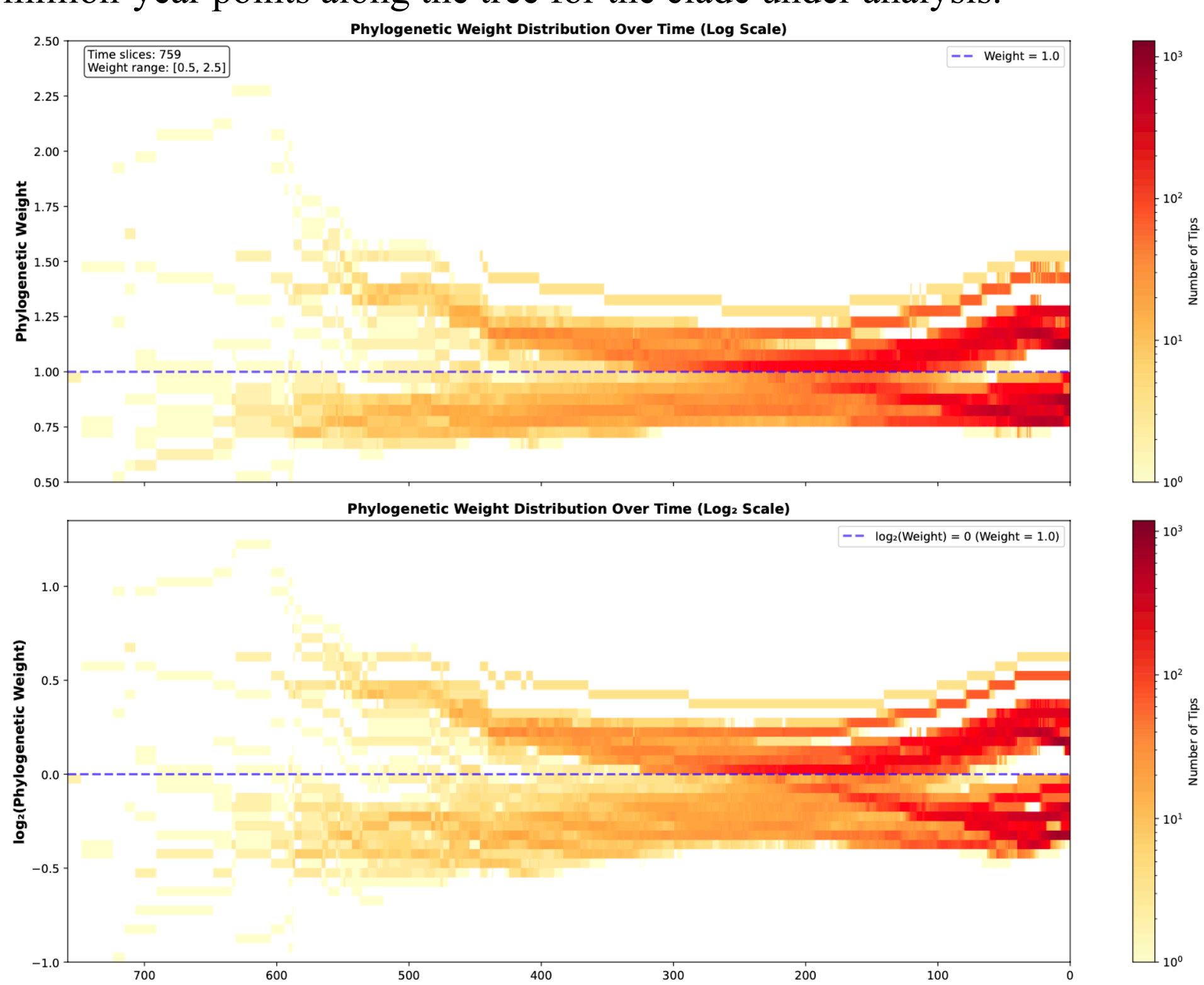

REFERENCES

1. J. Lv, P. Havlak, N. H. Putnam, Constraints on genes shape long-term conservation of macro-synteny in metazoan genomes. *BMC Bioinformatics* **12** (Suppl. 9), S11 (2011).

2. O. Simakov, J. Bredeson, K. Berkoff, F. Marletaz, T. Mitros, D. T. Schultz, B. L. O'Connell, P. Dear, D. E. Martinez, R. E. Steele, R. E. Green, C. N. David, D. S. Rokhsar, Deeply conserved synteny and the evolution of metazoan chromosomes. *Sci. Adv.* **8**, eabi5884 (2022).

3. D. T. Schultz, S. H. D. Haddock, J. V. Bredeson, R. E. Green, O. Simakov, D. S. Rokhsar, Ancient gene linkages support ctenophores as sister to other animals. *Nature* **618**, 110–117 (2023).

4. J. F. H. Strassert, I. Irisarri, T. A. Williams, F. Burki, A molecular timescale for eukaryote evolution with implications for the origin of red algal-derived plastids. *Nat. Commun.* **12**, 1879 (2021).

5. N. H. Putnam, T. Butts, D. E. K. Ferrier, R. F. Furlong, U. Hellsten, T. Kawashima, M. Robinson-Rechavi, E. Shoguchi, A. Terry, J.-K. Yu, E. Benito-Gutiérrez, I. Dubchak, J. Garcia-Fernàndez, J. J. Gibson-Brown, I. V. Grigoriev, A. C. Horton, P. J. de Jong, J. Jurka, V. V. Kapitonov, Y. Kohara, Y. Kuroki, E. Lindquist, S. Lucas, K. Osoegawa, L. A. Pennacchio, A. A. Salamov, Y. Satou, T. Sauka-Spengler, J. Schmutz, T. Shin-I, A. Toyoda, M. Bronner-Fraser, A. Fujiyama, L. Z. Holland, P. W. H. Holland, N. Satoh, D. S. Rokhsar, The amphioxus genome and the evolution of the chordate karyotype. *Nature* **453**, 1064–1071 (2008).

6. A. H. Sturtevant, E. Novitski, The homologies of the chromosome elements in the genus *Drosophila*. *Genetics* **26**, 517–541 (1941).

7. O. Simakov, F. Marlétaz, J.-X. Yue, B. O'Connell, J. Jenkins, A. Brandt, R. Calef, C.-H. Tung, T.-K. Huang, J. Schmutz, N. Satoh, J.-K. Yu, N. H. Putnam, R. E. Green, D. S. Rokhsar, Deeply conserved synteny resolves early events in vertebrate evolution. *Nat. Ecol. Evol.* **4**, 820–830 (2020).

8. S. Wright, On the probability of fixation of reciprocal translocations. *Am. Nat.* **75**, 513–522 (1941).

9. C. B. Albertin, S. Medina-Ruiz, T. Mitros, H. Schmidbaur, G. Sanchez, Z. Y. Wang, J. Grimwood, J. J. C. Rosenthal, C. W. Ragsdale, O. Simakov, D. S. Rokhsar, Genome and transcriptome mechanisms driving cephalopod evolution. *Nat. Commun.* **13**, 2427 (2022).

10. C. J. Wright, L. Stevens, A. Mackintosh, M. Lawniczak, M. Blaxter, Comparative genomics reveals the dynamics of chromosome evolution in Lepidoptera. *Nat. Ecol. Evol.* **8**, 777–790 (2024).

11. S. Tandonnet, G. D. Koutsovoulos, S. Adams, D. Cloarec, M. Parihar, M. L. Blaxter, A. Pires-daSilva, Chromosome-wide evolution and sex determination in the three-sexed nematode *Auanema rhodensis*. *G3* **9**, 1211–1230 (2019).

12. Drosophila 12 Genomes Consortium, Evolution of genes and genomes on the *Drosophila* phylogeny. *Nature* **450**, 203–218 (2007).

13. D. T. Schultz, E. A. C. Heath-Heckman, C. J. Winchell, D.-H. T. Kuo, Y.-S. Yu, F. Oberauer, K. Kocot, S.-J. Cho, O. Simakov, D. A. Weisblat, Acceleration of genome rearrangement in clitellate annelids. bioRxiv 2024.05.12.593736 [Preprint] (2024). https://doi.org/10.1101/2024.05.12.593736.

14. T. D. Lewin, I. J.-Y. Liao, Y.-J. Luo, Annelid comparative genomics and the evolution of massive lineage-specific genome rearrangement in bilaterians. *Mol. Biol. Evol.* **41**, msae172 (2024).

15. C. Vargas-Chávez, L. Benítez-Álvarez, G. I. Martínez-Redondo, L. Álvarez-González, J. Salces-Ortiz, K. Eleftheriadi, N. Escudero, N. Guiglielmoni, J.-F. Flot, M. Novo, A. Ruiz-Herrera, A. McLysaght, R. Fernández, An episodic burst of massive genomic rearrangements and the origin of non-marine annelids. *Nat. Ecol. Evol.* **9**, 1263–1279 (2025).

16. M. Belcaid, G. Casaburi, S. J. McAnulty, H. Schmidbaur, A. M. Suria, S. Moriano-Gutierrez, M. S. Pankey, T. H. Oakley, N. Kremer, E. J. Koch, A. J. Collins, H. Nguyen, S. Lek, I. Goncharenko-Foster, P. Minx, E. Sodergren, G. Weinstock, D. S. Rokhsar, M. McFall-Ngai, O. Simakov, J. S. Foster, S. V. Nyholm, Symbiotic organs shaped by distinct modes of genome evolution in cephalopods. *Proc. Natl. Acad. Sci. U.S.A.* **116**, 3030–3035 (2019).

17. H. Schmidbaur, A. Kawaguchi, T. Clarence, X. Fu, O. P. Hoang, B. Zimmermann, E. A. Ritschard, A. Weissenbacher, J. S. Foster, S. V. Nyholm, P. A. Bates, C. B. Albertin, E. Tanaka, O. Simakov, Emergence of novel cephalopod gene regulation and expression through large-scale genome reorganization. *Nat. Commun.* **13**, 2172 (2022).

18. L. D. Hurst, E. J. B. Williams, C. Pál, Natural selection promotes the conservation of linkage of co-expressed genes. *Trends Genet.* **18**, 604–606 (2002).

19. M. Irimia, J. J. Tena, M. S. Alexis, A. Fernandez-Miñan, I. Maeso, O. Bogdanovic, E. de la Calle-Mustienes, S. W. Roy, J. L. Gómez-Skarmeta, H. B. Fraser, Extensive conservation of ancient microsynteny across metazoans due to cis-regulatory constraints. *Genome Res.* **22**, 2356–2367 (2012).

20. Y. Yin, H. Fan, B. Zhou, Y. Hu, G. Fan, J. Wang, F. Zhou, W. Nie, C. Zhang, L. Liu, Z. Zhong, W. Zhu, G. Liu, Z. Lin, C. Liu, J. Zhou, G. Huang, Z. Li, J. Yu, Y. Zhang, Y. Yang, B. Zhuo, B. Zhang, J. Chang, H. Qian, Y. Peng, X. Chen, L. Chen, Z. Li, Q. Zhou, W. Wang, F. Wei, Molecular mechanisms and topological consequences of drastic chromosomal rearrangements of muntjac deer. *Nat. Commun.* **12**, 6858 (2021).

21. D. Duboule, The rise and fall of Hox gene clusters. *Development* **134**, 2549–2560 (2007).

22. B. Zimmermann, N. S. M. Robert, U. Technau, O. Simakov, Ancient animal genome architecture reflects cell type identities. *Nat. Ecol. Evol.* **3**, 1289–1293 (2019).

23. F. M. Real, S. A. Haas, P. Franchini, P. Xiong, O. Simakov, H. Kuhl, R. Schöpflin, D. Heller, M.-H. Moeinzadeh, V. Heinrich, T. Krannich, A. Bressin, M. F. Hartmann, S. A. Wudy, D. K. N. Dechmann, A. Hurtado, F. J. Barrionuevo, M. Schindler, I. Harabula, M. Osterwalder, M. Hiller, L. Wittler, A. Visel, B. Timmermann, A. Meyer, M. Vingron, R. Jiménez, S. Mundlos, D. G. Lupiáñez, The mole genome reveals regulatory rearrangements associated with adaptive intersexuality. *Science* **370**, 208–214 (2020).

24. F. Marlétaz, E. de la Calle-Mustienes, R. D. Acemel, C. Paliou, S. Naranjo, P. M. Martínez-García, I. Cases, V. A. Sleight, C. Hirschberger, M. Marcet-Houben, D. Navon, A. Andrescavage, K. Skvortsova, P. E. Duckett, Á. González-Rajal, O. Bogdanovic, J. H.

Gibcus, L. Yang, L. Gallardo-Fuentes, I. Sospedra, J. Lopez-Rios, F. Darbellay, A. Visel, J. Dekker, N. Shubin, T. Gabaldón, T. Nakamura, J. J. Tena, D. G. Lupiáñez, D. S. Rokhsar, J. L. Gómez-Skarmeta, The little skate genome and the evolutionary emergence of wing-like fins. *Nature* **616**, 495–503 (2023).

25. S. E. Gilbertson, H. C. Walter, K. Gardner, S. N. Wren, G. Vahedi, A. S. Weinmann, Topologically associating domains are disrupted by evolutionary genome rearrangements forming species-specific enhancer connections in mice and humans. *Cell Rep.* **39**, 110769 (2022).

26. Darwin Tree of Life Project Consortium, Sequence locally, think globally: The Darwin Tree of Life Project. *Proc. Natl. Acad. Sci. U.S.A.* **119**, e2115642118 (2022).

27. H. A. Lewin, G. E. Robinson, W. J. Kress, W. J. Baker, J. Coddington, K. A. Crandall, R. Durbin, S. V. Edwards, F. Forest, M. T. P. Gilbert, M. M. Goldstein, I. V. Grigoriev, K. J. Hackett, D. Haussler, E. D. Jarvis, W. E. Johnson, A. Patrinos, S. Richards, J. C. Castilla-Rubio, M.-A. van Sluys, P. S. Soltis, X. Xu, H. Yang, G. Zhang, Earth BioGenome Project: Sequencing life for the future of life. *Proc. Natl. Acad. Sci. U.S.A.* **115**, 4325–4333 (2018).

28. O. Simakov, G. P. Wagner, The application of irreversible genomic states to define and trace ancient cell type homologies. *Evodevo* **16**, 5 (2025).

29. J. Armstrong, G. Hickey, M. Diekhans, I. T. Fiddes, A. M. Novak, A. Deran, Q. Fang, D. Xie, S. Feng, J. Stiller, D. Genereux, J. Johnson, V. D. Marinescu, J. Alföldi, R. S. Harris, K. Lindblad-Toh, D. Haussler, E. Karlsson, E. D. Jarvis, G. Zhang, B. Paten, Progressive Cactus is a multiple-genome aligner for the thousand-genome era. *Nature* **587**, 246–251 (2020).

30. O. Rota-Stabelli, A. C. Daley, D. Pisani, Molecular timetrees reveal a Cambrian colonization of land and a new scenario for ecdysozoan evolution. *Curr. Biol.* **23**, 392–398 (2013).

31. R. A. Rohde, R. A. Muller, Cycles in fossil diversity. *Nature* **434**, 208–210 (2005).

32. J. K. Hane, T. Rouxel, B. J. Howlett, G. H. J. Kema, S. B. Goodwin, R. P. Oliver, A novel mode of chromosomal evolution peculiar to filamentous Ascomycete fungi. *Genome Biol.* **12**, R45 (2011).

33. O. Simakov, T. Kawashima, Independent evolution of genomic characters during major metazoan transitions. *Dev. Biol.* **427**, 179–192 (2017).

34. A. M. Aguinaldo, J. M. Turbeville, L. S. Linford, M. C. Rivera, J. R. Garey, R. A. Raff, J. A. Lake, Evidence for a clade of nematodes, arthropods and other moulting animals. *Nature* **387**, 489–493 (1997).

35. N. Lartillot, H. Philippe, Improvement of molecular phylogenetic inference and the phylogeny of Bilateria. *Philos. Trans. R. Soc. Lond. B Biol. Sci.* **363**, 1463–1472 (2008).

36. Y. Wang, R. B. Gasser, D. Charlesworth, Q. Zhou, Evolution of sexual systems, sex chromosomes and sex-linked gene transcription in flatworms and roundworms. *Nat. Commun.* **13**, 3239 (2022).

37. S. Ohno, *Evolution by Gene Duplication* (Springer Science & Business Media, 1970).

38. P. Dehal, J. L. Boore, Two rounds of whole genome duplication in the ancestral vertebrate. *PLOS Biol.* **3**, e314 (2005).

39. V. A. Lukhtanov, V. Dincă, M. Friberg, J. Šíchová, M. Olofsson, R. Vila, F. Marec, C. Wiklund, Versatility of multivalent orientation, inverted meiosis, and rescued fitness in holocentric chromosomal hybrids. *Proc. Natl. Acad. Sci. U.S.A.* **115**, E9610–E9619 (2018).

40. M. L. Leibowitz, C.-Z. Zhang, D. Pellman, Chromothripsis: A new mechanism for rapid karyotype evolution. *Annu. Rev. Genet.* **49**, 183–211 (2015).

41. P. J. Stephens, C. D. Greenman, B. Fu, F. Yang, G. R. Bignell, L. J. Mudie, E. D. Pleasance, K. W. Lau, D. Beare, L. A. Stebbings, S. McLaren, M.-L. Lin, D. J. McBride, I. Varela, S. Nik-Zainal, C. Leroy, M. Jia, A. Menzies, A. P. Butler, J. W. Teague, M. A. Quail, J. Burton, H. Swerdlow, N. P. Carter, L. A. Morsberger, C. Iacobuzio-Donahue, G. A. Follows, A. R. Green, A. M. Flanagan, M.

R. Stratton, P. A. Futreal, P. J. Campbell, Massive genomic rearrangement acquired in a single catastrophic event during cancer development. *Cell* **144**, 27–40 (2011).

42. J. F. Ryan, M. E. Mazza, K. Pang, D. Q. Matus, A. D. Baxevanis, M. Q. Martindale, J. R. Finnerty, Pre-bilaterian origins of the Hox cluster and the Hox code: Evidence from the sea anemone, *Nematostella vectensis*. *PLOS ONE* **2**, e153 (2007).

43. N. S. M. Robert, F. Sarigol, B. Zimmermann, A. Meyer, C. R. Voolstra, O. Simakov, Emergence of distinct syntenic density regimes is associated with early metazoan genomic transitions. *BMC Genomics* **23**, 143 (2022).

44. D. Destanović, "Comparative structural genomics of Octopus," thesis, University of Vienna (2023).

45. R. D. Acemel, I. Maeso, J. L. Gómez-Skarmeta, Topologically associated domains: A successful scaffold for the evolution of gene regulation in animals. *Wiley Interdiscip. Rev. Dev. Biol.* **6**, 10.1002/wdev.265 (2017).

46. T. F. Rogers, O. Simakov, Emerging questions on the mechanisms and dynamics of 3D genome evolution in spiralians. *Brief. Funct. Genomics* **22**, 533–542 (2023).

47. J. Krefting, M. A. Andrade-Navarro, J. Ibn-Salem, Evolutionary stability of topologically associating domains is associated with conserved gene regulation. *BMC Biol.* **16**, 87 (2018).

48. N. H. Lazar, K. A. Nevonen, B. O'Connell, C. McCann, R. J. O'Neill, R. E. Green, T. J. Meyer, M. Okhovat, L. Carbone, Epigenetic maintenance of topological domains in the highly rearranged gibbon genome. *Genome Res.* **28**, 983–997 (2018).

49. Y. Liao, X. Zhang, M. Chakraborty, J. J. Emerson, Topologically associating domains and their role in the evolution of genome structure and function in *Drosophila*. *Genome Res.* **31**, 397–410 (2021).

50. B. Zimmermann, J. D. Montenegro, S. M. C. Robb, W. J. Fropf, L. Weilguny, S. He, S. Chen, J. Lovegrove-Walsh, E. M. Hill, C.-Y. Chen, K. Ragkousi, D. Praher, D. Fredman, D. Schultz, Y. Moran, O. Simakov, G. Genikhovich, M. C. Gibson, U. Technau, Topological

structures and syntenic conservation in sea anemone genomes. *Nat. Commun.* **14**, 8270 (2023).

51. P. Heger, B. Marin, M. Bartkuhn, E. Schierenberg, T. Wiehe, The chromatin insulator CTCF and the emergence of metazoan diversity. *Proc. Natl. Acad. Sci. U.S.A.* **109**, 17507–17512 (2012).

52. P. G. Engström, S. J. Ho Sui, O. Drivenes, T. S. Becker, B. Lenhard, Genomic regulatory blocks underlie extensive microsynteny conservation in insects. *Genome Res.* **17**, 1898–1908 (2007).

53. L. McInnes, J. Healy, J. Melville, UMAP: Uniform Manifold Approximation and Projection for dimension reduction. arXiv:1802.03426 [stat.ML] (2018).

54. L. van der Maaten, G. Hinton, Visualizing data using t-SNE. *J. Mach. Learn. Res.* **9**, 2579–2605 (2008).

55. E. Parey, A. Louis, J. Montfort, O. Bouchez, C. Roques, C. Iampietro, J. Lluch, A. Castinel, C. Donnadieu, T. Desvignes, C. Floi Bucao, E. Jouanno, M. Wen, S. Mejri, R. Dirks, H. Jansen, C. Henkel, W.-J. Chen, M. Zahm, C. Cabau, C. Klopp, A. W. Thompson, M. Robinson-Rechavi, I. Braasch, G. Lecointre, J. Bobe, J. H. Postlethwait, C. Berthelot, H. Roest Crollius, Y. Guiguen, Genome structures resolve the early diversification of teleost fishes. *Science* **379**, 572–575 (2023).

56. P. J. Batut, X. Y. Bing, Z. Sisco, J. Raimundo, M. Levo, M. S. Levine, Genome organization controls transcriptional dynamics during development. *Science* **375**, 566–570 (2022).

57. M. S. Magri, D. Voronov, S. Foley, P. M. Martínez-García, M. Franke, G. A. Cary, J. M. Santos-Pereira, C. Cuomo, M. Fernández-Moreno, M. Portela, A. Gil-Galvez, R. D. Acemel, P. Paganos, C. Ku, J. Ranđelović, M. L. Rusciano, P. N. Firbas, J. L. Gómez-Skarmeta, V. F. Hinman, M. I. Arnone, I. Maeso, Deep conservation of cis-regulatory elements and chromatin organization in echinoderms uncover ancestral regulatory features of animal genomes. *Nat. Ecol. Evol.* **10**, 355–371 (2026).

58. G. R. Cavalheiro, C. Girardot, R. R. Viales, T. Pollex, T. B. N. Cao, P. Lacour, S. Feng, A. Rabinowitz, E. E. M. Furlong, CTCF, BEAF-32, and CP190 are not required for the establishment of TADs in early *Drosophila* embryos but have locus-specific roles. *Sci. Adv.* **9**, eade1085 (2023).

59. I. V. Kim, C. Navarrete, X. Grau-Bové, M. Iglesias, A. Elek, G. Zolotarov, N. S. Bykov, S. A. Montgomery, E. Ksiezopolska, D. Cañas-Armenteros, J. J. Soto-Angel, S. P. Leys, P. Burkhardt, H. Suga, A. de Mendoza, M. Martí-Renom, A. Sebé-Pedrós, Chromatin loops are an ancestral hallmark of the animal regulatory genome. *Nature* **642**, 1097–1105 (2025).

60. O. Simakov, T. Kawashima, F. Marlétaz, J. Jenkins, R. Koyanagi, T. Mitros, K. Hisata, J. Bredeson, E. Shoguchi, F. Gyoja, J.-X. Yue, Y.-C. Chen, R. M. Freeman, A. Sasaki, T. Hikosaka-Katayama, A. Sato, M. Fujie, K. W. Baughman, J. Levine, P. Gonzalez, C. Cameron, J. H. Fritzenwanker, A. M. Pani, H. Goto, M. Kanda, N. Arakaki, S. Yamasaki, J. Qu, A. Cree, Y. Ding, H. H. Dinh, S. Dugan, M. Holder, S. N. Jhangiani, C. L. Kovar, S. L. Lee, L. R. Lewis, D. Morton, L. V. Nazareth, G. Okwuonu, J. Santibanez, R. Chen, S. Richards, D. M. Muzny, A. Gillis, L. Peshkin, M. Wu, T. Humphreys, Y.-H. Su, N. H. Putnam, J. Schmutz, A. Fujiyama, J.-K. Yu, K. Tagawa, K. C. Worley, R. A. Gibbs, M. W. Kirschner, C. J. Lowe, N. Satoh, D. S. Rokhsar, J. Gerhart, Hemichordate genomes and deuterostome origins. *Nature* **527**, 459–465 (2015).

61. R. D. Acemel, J. J. Tena, I. Irastorza-Azcarate, F. Marlétaz, C. Gómez-Marín, E. de la Calle-Mustienes, S. Bertrand, S. G. Diaz, D. Aldea, J.-M. Aury, S. Mangenot, P. W. H. Holland, D. P. Devos, I. Maeso, H. Escrivá, J. L. Gómez-Skarmeta, A single three-dimensional chromatin compartment in amphioxus indicates a stepwise evolution of vertebrate Hox bimodal regulation. *Nat. Genet.* **48**, 336–341 (2016).

62. D. H. Erwin, Macroevolution is more than repeated rounds of microevolution. *Evol. Dev.* **2**, 78–84 (2000).

63. D. H. Erwin, The topology of evolutionary novelty and innovation in macroevolution. *Philos. Trans. R. Soc. Lond. B Biol. Sci.* **372**, 20160422 (2017).

64. A. Rhie, S. A. McCarthy, O. Fedrigo, J. Damas, G. Formenti, S. Koren, M. Uliano-Silva, W. Chow, A. Fungtammasan, J. Kim, C. Lee, B. J. Ko, M. Chaisson, G. L. Gedman, L. J. Cantin, F. Thibaud-Nissen, L. Haggerty, I. Bista, M. Smith, B. Haase, J. Mountcastle, S. Winkler, S. Paez, J. Howard, S. C. Vernes, T. M. Lama, F. Grutzner, W. C. Warren, C. N. Balakrishnan, D. Burt, J. M. George, M. T. Biegler, D. Iorns, A. Digby, D. Eason, B. Robertson, T. Edwards, M. Wilkinson, G. Turner, A. Meyer, A. F. Kautt, P. Franchini, H. W. Detrich III, H. Svardal, M. Wagner, G. J. P. Naylor, M. Pippel, M. Malinsky, M. Mooney, M. Simbirsky, B. T. Hannigan, T. Pesout, M. Houck, A. Misuraca, S. B. Kingan, R. Hall, Z. Kronenberg, I. Sović, C. Dunn, Z. Ning, A. Hastie, J. Lee, S. Selvaraj, R. E. Green, N. H. Putnam, I. Gut, J. Ghurye, E. Garrison, Y. Sims, J. Collins, S. Pelan, J. Torrance, A. Tracey, J. Wood, R. E. Dagnew, D. Guan, S. E. London, D. F. Clayton, C. V. Mello, S. R. Friedrich, P. V. Lovell, E. Osipova, F. O. Al-Ajli, S. Secomandi, H. Kim, C. Theofanopoulou, M. Hiller, Y. Zhou, R. S. Harris, K. D. Makova, P. Medvedev, J. Hoffman, P. Masterson, K. Clark, F. Martin, K. Howe, P. Flicek, B. P. Walenz, W. Kwak, H. Clawson, M. Diekhans, L. Nassar, B. Paten, R. H. S. Kraus, A. J. Crawford, M. T. P. Gilbert, G. Zhang, B. Venkatesh, R. W. Murphy, K.-P. Koepfli, B. Shapiro, W. E. Johnson, F. Di Palma, T. Marques-Bonet, E. C. Teeling, T. Warnow, J. M. Graves, O. A. Ryder, D. Haussler, S. J. O'Brien, J. Korlach, H. A. Lewin, K. Howe, E. W. Myers, R. Durbin, A. M. Phillippy, E. D. Jarvis, Towards complete and error-free genome assemblies of all vertebrate species. *Nature* **592**, 737–746 (2021).

65. V. McKenna, J. M. Archibald, R. Beinart, M. N. Dawson, U. Hentschel, P. J. Keeling, J. V. Lopez, J. M. Martín-Durán, J. M. Petersen, J. D. Sigwart, O. Simakov, K. R. Sutherland, M. Sweet, N. Talbot, A. W. Thompson, S. Bender, P. W. Harrison, J. Rajan, G. Cochrane, M. Berriman, M. K. N. Lawniczak, M. Blaxter, The Aquatic Symbiosis Genomics Project: Probing the evolution of symbiosis across the tree of life. *Wellcome Open Res.* **6**, 254 (2021).

66. J. Köster, S. Rahmann, Snakemake—A scalable bioinformatics workflow engine. *Bioinformatics* **28**, 2520–2522 (2012).

67. J. Huerta-Cepas, F. Serra, P. Bork, ETE 3: Reconstruction, analysis, and visualization of phylogenomic data. *Mol. Biol. Evol.* **33**, 1635–1638 (2016).

68. N. A. O'Leary, E. Cox, J. B. Holmes, W. R. Anderson, R. Falk, V. Hem, M. T. N. Tsuchiya, G. D. Schuler, X. Zhang, J. Torcivia, A. Ketter, L. Breen, J. Cothran, H. Bajwa, J. Tinne, P. A. Meric, W. Hlavina, V. A. Schneider, Exploring and retrieving sequence and metadata for species across the tree of life with NCBI Datasets. *Sci. Data* **11**, 732 (2024).

69. A. Zielezinski, M. Michalczyk, K. Dudczak, Aziele/fastapy: Fastapy 1.0.3, Zenodo (2024); https://doi.org/10.5281/zenodo.10462111.

70. H. Li, Protein-to-genome alignment with miniprot. *Bioinformatics* **39**, btad014 (2023).

71. W. McKinney, Data structures for statistical computing in Python, in Proceedings of the 9th Python in Science Conference, https://doi.org/10.25080/Majora-92bf1922-00a (2010), pp. 56–61.

72. The Pandas Development Team, Pandas-Dev/pandas: Pandas, Zenodo (2023); https://zenodo.org/records/10426137.

73. C. L. Schoch, S. Ciufo, M. Domrachev, C. L. Hotton, S. Kannan, R. Khovanskaya, D. Leipe, R. McVeigh, K. O'Neill, B. Robbertse, S. Sharma, V. Soussov, J. P. Sullivan, L. Sun, S. Turner, I. Karsch-Mizrachi, NCBI Taxonomy: A comprehensive update on curation, resources and tools. Database 2020, *baaa062* (2020).

74. E. Cox, M. T. N. Tsuchiya, S. Ciufo, J. Torcivia, R. Falk, W. R. Anderson, J. B. Holmes, V. Hem, L. Breen, E. Davis, A. Ketter, P. Zhang, V. Soussov, C. L. Schoch, N. A. O'Leary, NCBI Taxonomy: Enhanced access via NCBI Datasets. *Nucleic Acids Res*. **53**, D1711–D1715 (2025).

75. M. A. Ruggiero, D. P. Gordon, T. M. Orrell, N. Bailly, T. Bourgoin, R. C. Brusca, T. Cavalier-Smith, M. D. Guiry, P. M. Kirk, A higher level classification of all living organisms. *PLOS ONE* **10**, e0119248 (2015).

76. G. Giribet, G. D. Edgecombe, *Perspectives in Animal Phylogeny and Evolution: A Decade Later* (University of Padova Press, 2019).

77. M. Drábková, K. M. Kocot, K. M. Halanych, T. H. Oakley, L. L. Moroz, J. T. Cannon, A. Kuris, A. E. Garcia-Vedrenne, M. S. Pankey, E. A. Ellis, R. Varney, J. Štefka, J. Zrzavý, Different phylogenomic methods support monophyly of enigmatic "Mesozoa" (Dicyemida + Orthonectida, Lophotrochozoa). *Proc. Biol. Sci.* **289**, 20220683 (2022).

78. B. Buchfink, K. Reuter, H.-G. Drost, Sensitive protein alignments at tree-of-life scale using DIAMOND. *Nat. Methods* **18**, 366–368 (2021).

79. P. Virtanen, R. Gommers, T. E. Oliphant, M. Haberland, T. Reddy, D. Cournapeau, E. Burovski, P. Peterson, W. Weckesser, J. Bright, S. J. van der Walt, M. Brett, J. Wilson, K. J. Millman, N. Mayorov, A. R. J. Nelson, E. Jones, R. Kern, E. Larson, C. J. Carey, İ. Polat, Y. Feng, E. W. Moore, J. VanderPlas, D. Laxalde, J. Perktold, R. Cimrman, I. Henriksen, E. A. Quintero, C. R. Harris, A. M. Archibald, A. H. Ribeiro, F. Pedregosa, P. van Mulbregt, SciPy 1.0 Contributors, SciPy 1.0: Fundamental algorithms for scientific computing in Python. *Nat. Methods* **17**, 261–272 (2020).

80. S. C. Potter, A. Luciani, S. R. Eddy, Y. Park, R. Lopez, R. D. Finn, HMMER web server: 2018 update. *Nucleic Acids Res.* **46**, W200–W204 (2018).

81. S. Kumar, M. Suleski, J. M. Craig, A. E. Kasprowicz, M. Sanderford, M. Li, G. Stecher, S. B. Hedges, TimeTree 5: An expanded resource for species divergence times. *Mol. Biol. Evol.* **39**, msac174 (2022).

82. J. D. Hunter, Matplotlib: A 2D graphics environment. *Comput. Sci. Eng.* **9**, 90–95 (2007).

83. C. R. Harris, K. J. Millman, S. J. van der Walt, R. Gommers, P. Virtanen, D. Cournapeau, E. Wieser, J. Taylor, S. Berg, N. J. Smith, R. Kern, M. Picus, S. Hoyer, M. H. van Kerkwijk, M. Brett, A. Haldane, J. F. Del Río, M. Wiebe, P. Peterson, P. Gérard-Marchant, K. Sheppard, T. Reddy, W. Weckesser, H. Abbasi, C. Gohlke, T. E. Oliphant, Array programming with NumPy. *Nature* **585**, 357–362 (2020).

84. D. Destanović, D. T. Schultz, R. Styfhals, F. Cruz, J. Gómez-Garrido, M. Gut, I. Gut, G. Fiorito, O. Simakov, T. S. Alioto, G. Ponte, E. Seuntjens, A chromosome-level reference genome for the common octopus, *Octopus vulgaris* (Cuvier, 1797). *G3* **13**, jkad220 (2023).

85. H. Li, Minimap2: Pairwise alignment for nucleotide sequences. *Bioinformatics* **34**, 3094–3100 (2018).

86. F. Cabanettes, C. Klopp, D-GENIES: Dot plot large genomes in an interactive, efficient and simple way. *PeerJ* **6**, e4958 (2018).

87. H. Zhang, L. Song, X. Wang, H. Cheng, C. Wang, C. A. Meyer, T. Liu, M. Tang, S. Aluru, F. Yue, X. S. Liu, H. Li, Fast alignment and preprocessing of chromatin profiles with Chromap. *Nat. Commun.* **12**, 6566 (2021).

88. S. Lee, C. R. Bakker, C. Vitzthum, B. H. Alver, P. J. Park, Pairs and Pairix: A file format and a tool for efficient storage and retrieval for Hi-C read pairs. *Bioinformatics* **38**, 1729–1731 (2022).

89. N. Abdennur, L. A. Mirny, Cooler: Scalable storage for Hi-C data and other genomically labeled arrays. *Bioinformatics* **36**, 311–316 (2020).

90. F. Ramírez, V. Bhardwaj, L. Arrigoni, K. C. Lam, B. A. Grüning, J. Villaveces, B. Habermann, A. Akhtar, T. Manke, High-resolution TADs reveal DNA sequences underlying genome organization in flies. *Nat. Commun.* **9**, 189 (2018).

91. K. Kruse, C. B. Hug, J. M. Vaquerizas, FAN-C: A feature-rich framework for the analysis and visualisation of chromosome conformation capture data. *Genome Biol.* **21**, 303 (2020).

92. F. Li, L. Bian, J. Ge, F. Han, Z. Liu, X. Li, Y. Liu, Z. Lin, H. Shi, C. Liu, Q. Chang, B. Lu, S. Zhang, J. Hu, D. Xu, C. Shao, S. Chen, Chromosome-level genome assembly of the East Asian common octopus (*Octopus sinensis*) using PacBio sequencing and Hi-C technology. *Mol. Ecol. Resour.* **20**, 1572–1582 (2020).

93. D. Jiang, Q. Liu, J. Sun, S. Liu, G. Fan, L. Wang, Y. Zhang, I. Seim, S. An, X. Liu, Q. Li, X. Zheng, The gold-ringed octopus (*Amphioctopus fangsiao*) genome and cerebral single-nucleus transcriptomes provide insights into the evolution of karyotype and neural novelties. *BMC Biol.* **20**, 289 (2022).

94. A. R. Quinlan, I. M. Hall, BEDTools: A flexible suite of utilities for comparing genomic features. *Bioinformatics* **26**, 841–842 (2010).

95. G. R. Brown, V. Hem, K. S. Katz, M. Ovetsky, C. Wallin, O. Ermolaeva, I. Tolstoy, T. Tatusova, K. D. Pruitt, D. R. Maglott, T. D. Murphy, Gene: A gene-centered information resource at NCBI. *Nucleic Acids Res.* **43**, D36–D42 (2015).

96. S. A. Aleksander, J. Balhoff, S. Carbon, J. M. Cherry, The Gene Ontology knowledgebase in 2023. *Genetics* 224, iyad031 (2023).

97. Y. Benjamini, Y. Hochberg, Controlling the false discovery rate: A practical and powerful approach to multiple testing. *J. R. Stat. Soc. Ser. B Methodol.* **57**, 289–300 (1995).

98. D. V. Klopfenstein, L. Zhang, B. S. Pedersen, F. Ramírez, A. Warwick Vesztrocy, A. Naldi, C. J. Mungall, J. M. Yunes, O. Botvinnik, M. Weigel, W. Dampier, C. Dessimoz, P. Flick, H. Tang, GOATOOLS: A Python library for Gene Ontology analyses. *Sci. Rep.* **8**, 10872 (2018).

99. D. Karolchik, G. P. Barber, J. Casper, H. Clawson, M. S. Cline, M. Diekhans, T. R. Dreszer, P. A. Fujita, L. Guruvadoo, M. Haeussler, R. A. Harte, S. Heitner, A. S. Hinrichs, K. Learned, B. T. Lee, C. H. Li, B. J. Raney, B. Rhead, K. R. Rosenbloom, C. A. Sloan, M. L. Speir, A. S. Zweig, D. Haussler, R. M. Kuhn, W. J. Kent, The UCSC genome browser database: 2014 update. *Nucleic Acids Res.* **42**, D764–D770 (2014).

100. K. S. Pollard, M. J. Hubisz, K. R. Rosenbloom, A. Siepel, Detection of nonneutral substitution rates on mammalian phylogenies. *Genome Res*. **20**, 110–121 (2010).

101. A. Siepel, G. Bejerano, J. S. Pedersen, A. S. Hinrichs, M. Hou, K. Rosenbloom, H. Clawson, J. Spieth, L. W. Hillier, S. Richards, G. M. Weinstock, R. K. Wilson, R. A. Gibbs, W. J. Kent, W. Miller, D. Haussler, Evolutionarily conserved elements in vertebrate, insect, worm, *and yeast genomes. Genome Res*. **15**, 1034–1050 (2005).

102. The ENCODE Project Consortium, An integrated encyclopedia of DNA elements in the human genome. *Nature* **489**, 57–74 (2012).

103. F. Hammal, P. de Langen, A. Bergon, F. Lopez, B. Ballester, ReMap 2022: A database of Human, Mouse, Drosophila and Arabidopsis regulatory regions from an integrative analysis of DNA-binding sequencing experiments. *Nucleic Acids Res.* **50**, D316–D325 (2022).

104. M. Srivastava, E. Begovic, J. Chapman, N. H. Putnam, U. Hellsten, T. Kawashima, A. Kuo, T. Mitros, A. Salamov, M. L. Carpenter, A. Y. Signorovitch, M. A. Moreno, K. Kamm, J. Grimwood, J. Schmutz, H. Shapiro, I. V. Grigoriev, L. W. Buss, B. Schierwater, S. L. Dellaporta, D. S. Rokhsar, The *Trichoplax* genome and the nature of placozoans. *Nature* **454**, 955–960 (2008).

105. M. Srivastava, O. Simakov, J. Chapman, B. Fahey, M. E. A. Gauthier, T. Mitros, G. S. Richards, C. Conaco, M. Dacre, U. Hellsten, C. Larroux, N. H. Putnam, M. Stanke, M. Adamska, A. Darling, S. M. Degnan, T. H. Oakley, D. C. Plachetzki, Y. Zhai, M. Adamski, A. Calcino, S. F. Cummins, D. M. Goodstein, C. Harris, D. J. Jackson, S. P. Leys, S. Shu, B. J. Woodcroft, M. Vervoort, K. S. Kosik, G. Manning, B. M. Degnan, D. S. Rokhsar, The *Amphimedon queenslandica* genome and the evolution of animal complexity. *Nature* **466**, 720–726 (2010).

106. B. McClintock, The relation of a particular chromosomal element to the development of the nucleoli in *Zea mays*. *Z. Zellforsch. Mikrosk. Anat.* **21**, 294–326 (1934).

107. N. H. Putnam, M. Srivastava, U. Hellsten, B. Dirks, J. Chapman, A. Salamov, A. Terry, H. Shapiro, E. Lindquist, V. V. Kapitonov, J. Jurka, G. Genikhovich, I. V. Grigoriev, S. M. Lucas, R. E. Steele, J. R. Finnerty, U. Technau, M. Q. Martindale, D. S. Rokhsar, Sea anemone genome reveals ancestral eumetazoan gene repertoire and genomic organization. *Science* **317**, 86–94 (2007).

108. G. N. Luke, L. F. C. Castro, K. McLay, C. Bird, A. Coulson, P. W. H. Holland, Dispersal of NK homeobox gene clusters in amphioxus and humans. *Proc. Natl. Acad. Sci. U.S.A.* **100**, 5292–5295 (2003).

109. W. F. Doolittle, C. Sapienza, Selfish genes, the phenotype paradigm and genome evolution. *Nature* **284**, 601–603 (1980).

110. L. E. Orgel, F. H. Crick, Selfish DNA: The ultimate parasite. *Nature* **284**, 604–607 (1980).

111. G. Dover, W. F. Doolittle, Modes of genome evolution. *Nature* **288**, 646–647 (1980).

112. R. Stanyon, B. Chiarelli, Mode and tempo in primate chromosome evolution: Implications for hylobatid phylogeny. *J. Hum. Evol.* **12**, 305–315 (1983).

113. M. W. Bonierbale, R. L. Plaisted, S. D. Tanksley, RFLP maps based on a common set of clones reveal modes of chromosomal evolution in potato and tomato. *Genetics* **120**, 1095–1103 (1988).

114. M. Höglund, D. Gisselsson, G. B. Hansen, T. Säll, F. Mitelman, Ovarian carcinoma develops through multiple modes of chromosomal evolution. *Cancer Res.* **63**, 3378–3385 (2003).

115. A. Erives, B. Fritzsch, A screen for gene paralogies delineating evolutionary branching order of Early Metazoa. *G3* **10**, 811–826 (2020).

116. T. Dobzhansky, A. H. Sturtevant, Inversions in the chromosomes of *Drosophila pseudoobscura*. *Genetics* **23**, 28–64 (1938).

117. A. H. Sturtevant, C. C. Tan, The comparative genetics of *Drosophila pseudoobscura* and *D. melanogaster*. *J. Genet.* **34**, 415–432 (1937).

118. H. J. Muller, "Bearing of the *Drosophila* work on systematics," in *The New Systematics*, J. Huxley, Ed. (Oxford Univ. Press, 1940), pp. 185–268.

119. J. H. Renwick, The mapping of human chromosomes. *Annu. Rev. Genet.* **5**, 81–120 (1971).

120. E. Passarge, B. Horsthemke, R. A. Farber, Incorrect use of the term synteny. *Nat. Genet.* **23**, 387 (1999).

121. D. Schlessinger, J. L. Mandel, A. P. Monaco, D. L. Nelson, H. F. Willard, Report and abstracts of the Fourth International Workshop on Human X Chromosome Mapping 1993. *Cytogenet. Cell Genet.* 64, 147–194 (1993).

122. K. S. Thomson, Explanation of large scale extinctions of lower vertebrates. *Nature* **261**, 578–580 (1976).

123. R. K. Bambach, Phanerozoic biodiversity mass extinctions. *Annu. Rev. Earth Planet. Sci.* **34**, 127–155 (2006).

124. S. E. Peters, Geologic constraints on the macroevolutionary history of marine animals. *Proc. Natl. Acad. Sci. U.S.A.* **102**, 12326–12331 (2005).

125. S. M. K. Glasauer, S. C. F. Neuhauss, Whole-genome duplication in teleost fishes and its evolutionary consequences. *Mol. Genet. Genomics* **289**, 1045–1060 (2014).

126. P. N. Inglis, G. Ou, M. R. Leroux, J. M. Scholey, The sensory cilia of *Caenorhabditis elegans*. *WormBook* **1–22**, 10.1895/wormbook.1.126.2 (2007).

127. J. T. Orozco, K. P. Wedaman, D. Signor, H. Brown, L. Rose, J. M. Scholey, Movement of motor and cargo along cilia. *Nature* **398**, 674 (1999).

128. D. Scheuner, B. Song, E. McEwen, C. Liu, R. Laybutt, P. Gillespie, T. Saunders, S. Bonner-Weir, R. J. Kaufman, Translational control is required for the unfolded protein response and in vivo glucose homeostasis. *Mol. Cell* **7**, 1165–1176 (2001).

129. M. Forgac, Vacuolar ATPases: Rotary proton pumps in physiology and pathophysiology. *Nat. Rev. Mol. Cell Biol.* **8**, 917–929 (2007).

130. J.-I. Satoh, N. Kawana, Y. Yamamoto, Pathway analysis of ChIP-seq-based NRF1 target genes suggests a logical hypothesis of their involvement in the pathogenesis of neurodegenerative diseases. *Gene Regul. Syst. Bio.* **7**, 139–152 (2013).
