# Supplementary material for: Topological mixing and irreversibility in animal chromosome evolution
Source: Sci Adv. 2026 Aug 19;12(34):eadz5561. doi: 10.1126/sciadv.adz5561 (PMC13508932; doi:10.1126/sciadv.adz5561)
Supplement: Supplementary file 2 [file sciadv.adz5561_supplementary_file_1.zip › adz5561_supplementary_file_1.html]

Subsample allsamples nn=250 min\_dist=1.0
